# Incorporation of bridged nucleic acids into CRISPR RNAs improves Cas9 endonuclease specificity

**DOI:** 10.1038/s41467-018-03927-0

**Published:** 2018-04-13

**Authors:** Christopher R. Cromwell, Keewon Sung, Jinho Park, Amanda R. Krysler, Juan Jovel, Seong Keun Kim, Basil P. Hubbard

**Affiliations:** 1 Department of Pharmacology, University of Alberta, Edmonton, AB T6G 2R7 Canada; 2 Department of Chemistry, Seoul National University, Seoul, 08826 Republic of Korea; 3 The Applied Genomics Core, Office of Research, University of Alberta, Edmonton, AB T6G 2E1 Canada

## Abstract

Off-target DNA cleavage is a paramount concern when applying CRISPR-Cas9 gene-editing technology to functional genetics and human therapeutic applications. Here, we show that incorporation of next-generation bridged nucleic acids (2′,4′-BNA^NC^[N-Me]) as well as locked nucleic acids (LNA) at specific locations in CRISPR-RNAs (crRNAs) broadly reduces off-target DNA cleavage by Cas9 in vitro and in cells by several orders of magnitude. Using single-molecule FRET experiments we show that BNA^NC^ incorporation slows Cas9 kinetics and improves specificity by inducing a highly dynamic crRNA–DNA duplex for off-target sequences, which shortens dwell time in the cleavage-competent, “zipped” conformation. In addition to describing a robust technique for improving the precision of CRISPR/Cas9-based gene editing, this study illuminates an application of synthetic nucleic acids.

## Abstract (web summary)

Minimizing off-target effects is an important concern for therapeutic applications of CRISPR-Cas9. Here, the authors show that incorporating bridged or locked nucleic acids into crRNA improves editing kinetics and reduces off-target cleavage.

## Introduction

The Clustered Regularly Interspaced Short Palindromic Repeat (CRISPR)–Cas9 complex was originally characterized as a component of prokaryotic immune systems^1^, but has now become a widely used tool for genome editing applications^2^. CRISPR-Cas9 has been applied to the generation of knockout and knock-in organisms ranging from yeast to mice^3^, functional genomics^4^ and epigenetic^5^ screens, and proof-of-principle studies aimed at correcting genetic disease in mammals^6,7^. Two non-coding RNA elements direct sequence-specific DNA cleavage by the Cas9 system^8^. The CRISPR-RNA (crRNA) contains a 20-nucleotide (nt) RNA sequence complementary to the target DNA sequence, while the transactivating crRNA (tracrRNA) acts as a bridge between the crRNA and Cas9 enzyme^9^. Hybridization of these RNA elements together constitute a guide RNA (gRNA); they can also be covalently linked to produce a chimeric single-guide RNA (sgRNA)^9^. Recognition of a target sequence by Cas9 first involves the identification of an upstream protospacer adjacent motif (PAM) (5′-NGG-3′ in *S. pyogenes*) on the target DNA strand, followed by local DNA melting and hybridization of the first 10–12 bp of the 3′ end of the crRNA sequence (seed pairing), and formation an R-loop structure^2,9,10^. Complete hybridization between the guide segment and target DNA drives conformational changes in the HNH and RuvC nuclease domains on Cas9 that result in DNA cleavage 3-bp upstream of the PAM^8,11,12^. While mutations within the PAM sequence effectively abolish Cas9 cleavage activity^2,13^, mutations within the target sequence may be permitted^14^, resulting in cleavage of off-target DNA sequences.

Cas9 DNA cleavage specificity is highly dependent on the crRNA sequence and correlates with target-crRNA folding stability^15^. A number of approaches have been deployed to improve Cas9 specificity^16^, including the use of algorithms to computationally design gRNAs with minimal off-target activity^16^, a paired Cas9 nickase system that employs two gRNAs for target recognition^17^, and new delivery strategies displaying burst kinetics, such as Cas9 ribonucleoprotein (RNP) delivery^18^. In addition, several groups have engineered highly specific variants of Cas9, such as eSpCas9^19^, SpCas9-HF1^20^, and HypaCas9^21^, by mutating residues on Cas9 involved in the formation of non-specific DNA interactions. Despite these advances, off-target cutting and generation of accessory mutations remains a significant barrier for Cas9-based gene editing^16^.

While numerous studies have focused on engineering or otherwise modifying the Cas9 enzyme^17,19–22^, few have investigated the possibility of altering the sequence or structure of its crRNA to improve specificity^16^. Reducing the number of nucleotides in the spacer sequence from 20 to 17–18 bp (tru-guides) improves Cas9 specificity, but reduces on-target cleavage efficiency^23^. Interestingly, bridged nucleic acids (BNAs) have previously been shown to improve mismatch discrimination in nucleic acid duplexes^24^. We hypothesized that incorporation of these synthetic nucleotides into crRNAs could improve Cas9 DNA cleavage specificity. Moreover, previous studies have demonstrated that chemical modification of crRNAs with 2′-fluoro-ribose^25^, 2′-*O*-methyl 3′ phosphorothioate (MS)^26^, and other moieties^27^ increases gene editing efficiency by improving gRNA stability in cells and in vivo^28^, suggesting another potential benefit of this technology.

**Fig. 1:**
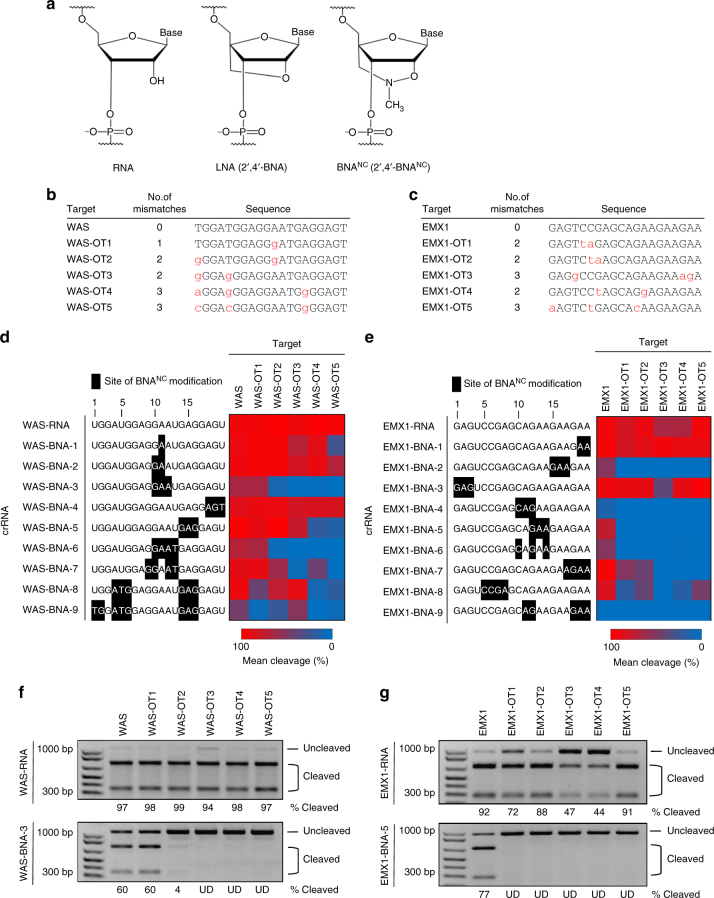
BNA^NC^ incorporation reduces off-target cleavage in vitro. **a** Chemical structures of RNA, LNA (2′,4′-BNA), and BNA^NC^ (2′,4′-BNA^NC^ [NMe]) nucleotides. **b** WAS and **c** EMX1 on-target and off-target sequences used for in vitro and cellular cleavage assays. Mismatches are indicated by red lowercase lettering. Heat map showing in vitro cleavage specificity for the unmodified crRNA and 9 BNA^NC^-modified crRNAs toward either **d** WAS or **e** EMX1 on-target and off-target sequences (as listed in Fig. 1b, c); mean shown (*n* = 2). crRNA and BNA^NC^-modified sequences are shown to the left of the corresponding heat map. BNA^NC^ modifications are indicated in black. Targets that were highly cleaved in vitro are indicated in red, while targets that were not cleaved are indicated in blue. Gel showing relative cleavage efficiencies of the unmodified and most specific BNA^NC^-modified crRNAs on a 1-kb DNA fragment containing either the **f** WAS or **g** EMX1 on-target and off-target sequences. The two bottom bands are cleavage products, while the top band is full-length substrate. The molar ratio of Cas9 RNP complex to target DNA was 30:1 for these experiments. Quantification of cleavage percentages was determined using densitometry (ImageJ), and are shown below each lane. Lanes in which no cleavage products were observed are marked as undetected (UD). Values used to generate heatmaps are presented in Supplementary Table 1

First-generation BNAs, or locked nucleic acids (LNAs) (Fig. 1a), are conformationally restricted RNA nucleotides in which the 2′ oxygen in the ribose forms a covalent bond to the 4′ carbon, inducing N-type (C3′-endo) sugar puckering and a preference for an A-form helix^24^. LNAs display improved base stacking and thermal stability compared to RNA, resulting in highly efficient binding to complementary nucleic acids and improved mismatch discrimination^24,29^. They also display enhanced nuclease resistance^29^. LNAs have been successfully used in numerous applications ranging from single-nucleotide polymorphism (SNP) detection assays^29^ to siRNA^30^. However, LNAs do have several limitations^31^. For example, oligonucleotides with multiple consecutive LNAs are unable to form nucleic acid triplex structures due to their rigidity^32,33^. Furthermore, LNA-modified antisense oligonucleotides induce hepatotoxicity in mice^34^. Next-generation N-methyl substituted BNAs (2′,4′-BNA^NC^[N-Me]) (Fig. 1a) were designed to circumvent some of these issues^31^. The six-membered bridged structure in BNA^NC^s provides more conformational flexibility for nucleic acid binding and greater nuclease resistance due to steric bulk^31^. Recent work also suggests that BNA^NC^ nucleotides are less toxic than LNA nucleotides when delivered to cultured cells^35^. Here, we show that incorporation of BNA^NC^s and LNAs at specific positions within crRNAs broadly improves Cas9 DNA cleavage specificity.

## Results

### BNA^NC^ incorporation reduces off-target cleavage in vitro

To test the hypothesis that incorporation of BNA^NC^-modified nucleotides into crRNAs improves Cas9 cleavage specificity, we selected two previously characterized crRNAs directed toward the *WAS*^36^ and *EMX1* genes^37^, for which in vitro and cellular off-target sites had previously been identified (Fig. 1b, c), and designed variants with BNA^NC^ substitutions. Previous work has demonstrated that local mismatch discrimination can be improved in DNA–DNA hybrids when LNAs are incorporated in the vicinity of mismatched bases, with an LNA triplet centered on the mismatch yielding the best results^24^. Therefore, we generated a series of 9 crRNAs with sequential or alternating substitutions of 1, 2, 3 or 4 BNA^NC^s (or in pairs) corresponding to the key mismatch positions of the five most abundant cellular off-target sites of the WAS and EMX1 crRNAs, respectively (Fig. 1b–e). Using an in vitro cleavage assay, we screened the ability of Cas9 to cleave the on-target sequence and off-target sequences using the original crRNAs and these BNA^NC^-substituted crRNAs (WAS/EMX1-BNA-1-9) at two different concentrations (Fig. 1d–g, Supplementary Fig. 1, Supplementary Tables 1 and 2). Surprisingly, we found that substitution of the centrally located GAA triplet in positions 10–12 of the WAS crRNA with BNA^NC^ (WAS-BNA-3) abolished off-target cleavage on all but one of the off-target sequences (Fig. 1d, f, Supplementary Fig. 1a, c). Interestingly, the off-target sequence that was unaffected contained a single A–G mismatch located within the substituted triplet. crRNAs containing substitutions of only 1 or 2 BNA^NC^s, and substitutions in flanking positions within the crRNA demonstrated more heterogeneous effects, but still generally improved Cas9 specificity (Fig. 1d, Supplementary Fig. 1a). A similar trend was observed for the EMX1 crRNAs, at both low and high concentrations of RNP complex (Fig. 1e, g, Supplementary Fig. 1b, d, Supplementary Tables 1 and 2). Remarkably, BNA^NC^ substitutions at positions 12–14 of the EMX1 crRNA (EMX1-BNA-5) abolished cleavage on all five off-target sites tested, while only marginally decreasing on-target cleavage activity (Fig. 1e, g, Supplementary Fig. 1b, d). Discontinuous BNA^NC^ substitutions at positions 10, 12, and 14 (EMX1-BNA-6) yielded similar improvements but with lower on-target activity, while EMX1-BNA-4 and EMX1-BNA-9 showed very low activity, possibly due to mis-folding of the crRNA (Fig. 1e).

### BNA^NC^ incorporation broadly improves specificity in vitro

CRISPR-Cas9 is typically not effective in resolving SNPs^38^ or single-nucleotide mismatches residing within the PAM-distal portion of the guide sequence^39^. Based on our finding that BNA^NC^-substituted crRNAs improve specificity, we speculated that they might improve discrimination of single mismatch off-target sequences. To test this hypothesis, we generated a series of target sequences corresponding to the WAS and EMX1 sites bearing individual mutations at 2 bp intervals and assayed their ability to be cleaved in vitro by Cas9 using either the unmodified crRNAs, or their most specific BNA^NC^-modified counterparts. For the WAS sequence, we found that WAS-BNA-3 dramatically improved single mismatch discrimination at both PAM-proximal and PAM-distal regions of the target sequence, relative to the control, but had little effect on mismatches located in regions directly overlapping BNA^NC^ substitutions (Supplementary Fig. 2a, Supplementary Table 3). Similarly, for the EMX1 sequence, EMX1-BNA-5 displayed improved specificity toward off-target sequences bearing single mutations in the PAM-proximal and PAM–distal regions, compared to the unmodified crRNA (Supplementary Fig. 2b, Supplementary Table 3).

**Fig. 2:**
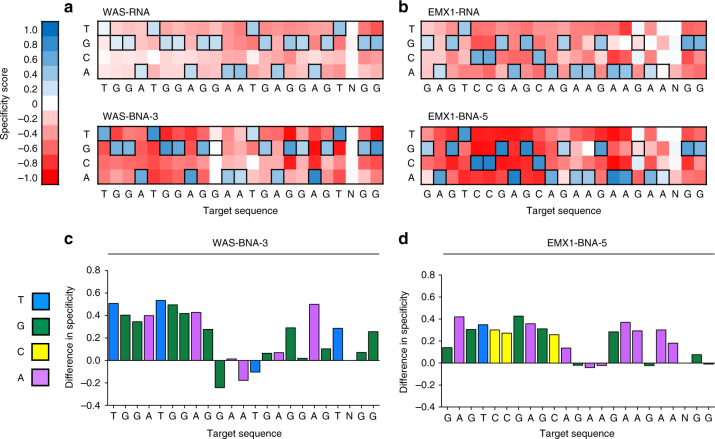
BNA^NC^ incorporation broadly improves specificity in vitro. Heat maps showing DNA cleavage specificity scores across >10^12^ off-target sequences for either unmodified (top) or BNA^NC^-modified crRNAs (bottom) targeting **a** WAS or **b** EMX1. Specificity scores of 1.0 (dark blue) correspond to 100% enrichment for, while scores of −1.0 (dark red) correspond to 100% enrichment against a specific base-pair at a specific position. Black boxes denote the intended target nucleotides. Bar graph showing the quantitative difference in specificity score at each position in the 20 base-pair target site and 2 base-pair PAM (N of NGG excluded), between the unmodified and BNA^NC^-modified crRNA for **c** WAS or **d** EMX1 target sequences. A score of zero indicates no change in specificity. Difference in specificity was calculated as specificity score_BNA_^NC^−specificity score_RNA_. Experiments were performed with 200 nM pre-selection library and 1000 nM Cas9 RNP complex

To globally assess how BNA^NC^ modification of crRNAs influences Cas9 specificity, we employed a previously described in vitro high-throughput specificity profiling assay^40–42^. Briefly, this technique selects DNA sequences that have undergone cleavage from a library of >10^12^ off-target sequences, containing a 10-fold coverage of all sequences with ≤8 mutations relative to the on-target sequence^40–42^. We performed this cleavage assay at both high and low concentrations of Cas9 RNP complex. We observed that WAS-BNA-3 and EMX1-BNA-5 broadly reduced the frequency of off-target cleavage on sequences containing 3–8 mutations relative to the on-target sequence, compared to the unmodified crRNAs (Supplementary Fig. 3). Moreover, we saw a significant reduction in the mean number of mutations in each selected sequence for the BNA^NC^-modified crRNAs, relative to their unmodified counterparts (Supplementary Table 4). Using this dataset, we next calculated enrichment scores for each base at each position within the target sequence for the crRNAs (Fig. 2a, b). We found that specificity was dramatically improved for both the WAS-BNA-3 and EMX1-BNA-5 crRNAs at nearly all positions in their respective target sequences (relative to the unmodified crRNAs), except those in the vicinity of the BNA^NC^ substitutions (Fig. 2a–d, Supplementary Fig. 4). Collectively, these results establish that incorporation of BNA^NC^ nucleotides into central positions of crRNAs broadly improves Cas9 specificity in vitro.

### BNA^NC^-modified crRNAs are compatible with Cas9 variants

Recently, variants of Cas9 with improved specificity have been engineered^19–21^. To examine if BNA^NC^-substituted crRNAs could be used in conjunction with these variants to further boost specificity, we tested the activity of eSpCas9, a Cas9 variant with substitutions that reduce non-specific interactions with the non-complementary DNA strand^19^, in vitro using our WAS and EMX1 on-target and off-target sequences and the corresponding unmodified crRNAs. For the WAS sequence, we found that eSpCas9 reduced off-target cleavage of WAS-OT3 and WAS-OT5, but had little effect on WAS-OT1, WAS-OT2, and WAS-OT4, compared to SpCas9 (Supplementary Fig. 5). Next, we repeated the cleavage assay of the WAS on-target and off-target sequences using eSpCas9 in combination with WAS-BNA-5. We selected this modified crRNA since its specificity improvements were complementary to those of eSpCas9. Strikingly, we found that the combination was additive, resulting in elimination of nearly all off-target activity (Supplementary Fig. 5a, c). These results demonstrate that BNA^NC^-modified crRNAs can complement the specificity enhancements of next-generation Cas9 variants.

### BNA^NC^ incorporation improves Cas9 specificity in cells

**Fig. 3:**
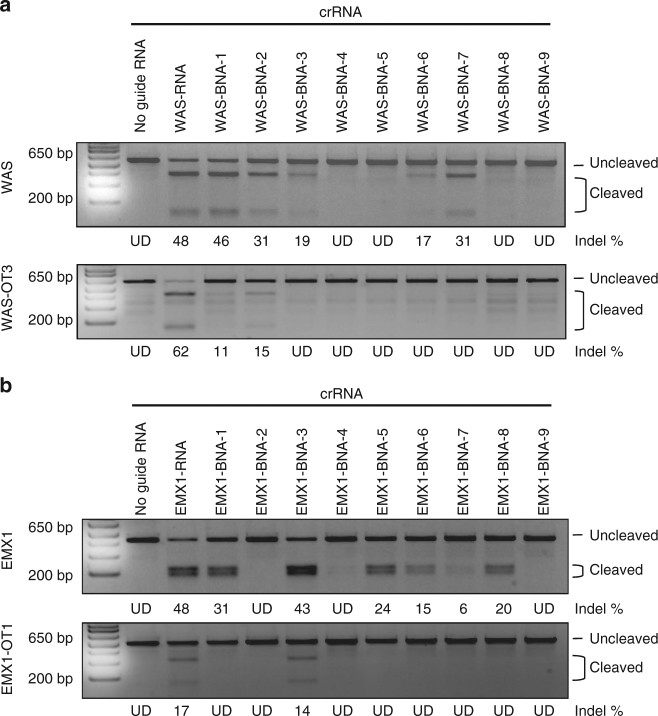
BNA^NC^ incorporation increases Cas9 specificity in cells. Gel showing relative cellular cleavage efficiencies of the unmodified, and 9 BNA^NC^-modified crRNAs targeting **a** *WAS* or **b** *EMX1* on-target (top) or off-target (bottom) sequences, as determined by T7 endonuclease I digestion. Mock transfections lacking guide RNA were used as controls. Modification frequencies were determined using densitometry (ImageJ) and are indicated below each lane. Lanes in which no cleavage products were observed are marked as undetected (UD)

To test if the improved specificity of the BNA^NC^-modified crRNAs observed in vitro translates into reduced off-target cleavage in cells, we transfected U2OS and HeLa cells stably expressing Cas9 with either unmodified or each of the 9 BNA^NC^-modified gRNAs corresponding to either the *WAS* or *EMX1* loci. Initially, we examined the activity of these gRNAs in cells by measuring cleavage of the on-target site and one off-target site using the T7 endonuclease I assay^43^. Consistent with our in vitro findings, we found that BNA^NC^-modified crRNAs generally induced lower cleavage rates at the off-target site (Fig. 3). We also observed that on-target activity was reduced in several instances. To quantitatively measure the on-target and off-target cleavage rates of WAS-BNA-3 and EMX1-BNA-5 and their unmodified counterparts in cells, we performed high-throughput sequencing. As shown in Table 1, while cleavage of the on-target site and OT1 was reduced by a factor of ~2–3-fold in HeLa cells, cleavage of OT2, OT3, OT4, and OT5 was reduced by >17,000-fold, >24,000-fold, >11,000-fold, and >24,000-fold, respectively (estimating the lower limit of detection to be 0.003%) using WAS-BNA-3. We observed similar specificity improvements using EMX1-BNA-5, compared to the unmodified EMX1 crRNA, in both cell types (Table 2, statistics and *P*-values in Supplementary Table 5). Finally, to determine if BNA^NC^-modified crRNAs alter how Cas9 cuts DNA, or its subsequent repair, we compared both the size and location of insertions/deletions generated using WAS-BNA-3 or EMX1-BNA-5 to those generated using their unmodified crRNA counterparts. We found that the pattern of indel formation was highly similar in both cases (Supplementary Figs. 6–8). These data establish BNA^NC^-modification of crRNAs as a new strategy to improve Cas9 DNA cleavage specificity in cells.

**Table 1:** Cellular modification rates using *WAS*-targeting crRNAs

	U2OS-Cas9	U2OS-Cas9	U2OS-Cas9	HeLa-Cas9	HeLa-Cas9	HeLa-Cas9
	No gRNA	WAS-RNA	WAS-BNA-3	No gRNA	WAS-RNA	WAS-BNA-3
WAS	<0.003	58.392	14.572	<0.003	56.020	19.475
WAS-OT1	0.003	17.548	9.400	0.004	18.135	8.726
WAS-OT2	0.092	59.553	0.034	0.120	53.400	<0.003
WAS-OT3	<0.003	73.142	<0.003	<0.003	70.859	<0.003
WAS-OT4	<0.003	36.506	<0.003	<0.003	33.151	<0.003
WAS-OT5	<0.003	75.239	<0.003	<0.003	71.119	<0.003

Table summarizing modification frequencies of on-target and off-target sequences in U2OS-Cas9 and HeLa-Cas9 cells using either unmodified or BNA^NC^-modified crRNAs targeting *WAS*, as determined by high-throughput sequencing. Modification frequencies were calculated by dividing the number of sequences bearing insertions or deletions (indels) in the target site by the total number of sequences and multiplied by 100 to give a percent. Mock transfections lacking gRNA were used as controls (see Supplementary Table 5 for additional data)

**Table 2:** Cellular modification rates using *EMX1*-targeting crRNAs

	U2OS-Cas9	U2OS-Cas9	U2OS-Cas9	HeLa-Cas9	HeLa-Cas9	HeLa-Cas9
	No gRNA	EMX1-RNA	EMX1-BNA-5	No gRNA	EMX1-RNA	EMX1-BNA-5
EMX1	0.004	69.181	27.393	<0.003	69.118	25.649
EMX1-OT1	0.005	12.233	<0.003	0.009	11.677	<0.003
EMX1-OT2	0.031	1.007	0.020	0.025	0.607	0.020
EMX1-OT3	0.068	<0.003	0.047	0.005	<0.003	<0.003
EMX1-OT4	0.073	<0.003	<0.003	0.042	0.016	0.051
EMX1-OT5	<0.003	0.113	0.002	0.060	0.141	<0.003

Table summarizing modification frequencies of on-target and off-target sequences in U2OS-Cas9 and HeLa-Cas9 cells using either unmodified or BNA^NC^-modified crRNAs targeting *EMX1*, as determined by high-throughput sequencing. Modification frequencies were calculated by dividing the number of sequences bearing insertions or deletions (indels) in the target site by the total number of sequences and multiplied by 100 to give a percent. Mock transfections lacking gRNA were used as controls (see Supplementary Table 5 for additional data)

### BNA^NC^s improve specificity via a conformational mechanism

We next investigated the mechanism underlying the specificity improvements of BNA^NC^-modified crRNAs. At least five distinct stages in the Cas9 cleavage reaction have been resolved: tracrRNA/crRNA loading onto Cas9, binding of the RNP complex to target DNA, DNA melting and PAM-proximal hybridization (open conformation), complete R-loop formation (zipped conformation), and structural rearrangement of the nuclease domains leading to cleavage^44^. To identify which of these stages is altered by BNA^NC^-modified crRNAs, we performed a variety of biochemical and biophysical experiments. We found that altering the tracrRNA:crRNA ratio in on-target and off-target cleavage reactions using either WAS-RNA or WAS-BNA-3 yielded similar effects (WAS-OT3 was not cleaved when WAS-BNA-3 crRNA was used), suggesting that BNA^NC^ incorporation does not alter tracrRNA hybridization (Supplementary Fig. 9a, b). In addition, titrations using different concentrations of annealed gRNA, whole RNP complex, and target DNA produced similar results between WAS-RNA and WAS-BNA-3 cleavage reactions with the on-target WAS sequence, while cleavage of WAS-OT3 was abolished in all cases when WAS-BNA-3 crRNA was used (Supplementary Fig. 9c–h). Using an electrophoretic mobility shift assay (EMSA), we observed that BNA^NC^ incorporation did not alter the ability of nuclease-deficient Cas9 (dCas9) to bind to DNA containing both on-target and off-target sequences (Supplementary Fig. 10).

**Fig. 4:**
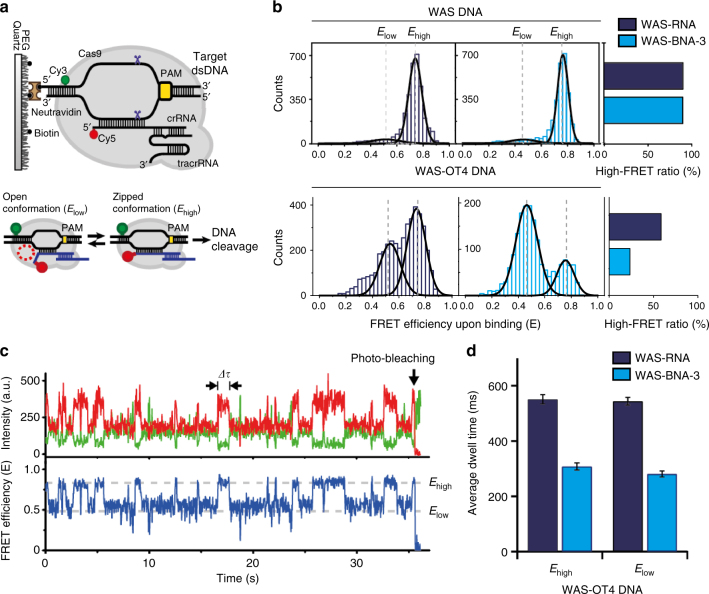
BNA^NC^ incorporation influences conformational transitions. **a** Schematic diagram for smFRET experiments showing a Cas9 RNP complex consisting of Cy5-labeled crRNA, tracrRNA, and Cas9, bound to a Cy3-labeled dsDNA immobilized on a quartz surface. **b** Histograms showing FRET efficiency after equilibration for the WAS DNA (upper) or WAS-OT4 DNA (lower) target sequences using WAS-RNA (dark blue) or WAS-BNA-3 (light blue) crRNAs; black curves represent Gaussian fits. **c** Time trace for Cas9 on WAS-OT4 DNA using WAS-BNA-3 crRNA indicating repetitive transitions between the open and zipped conformations. Dwell time in each conformation is indicated as ∆*τ*. **d** Comparison of Cas9 dwell times between WAS-RNA and WAS-BNA-3 crRNA using the WAS-OT4 DNA template; mean ± SD shown

To study changes in crRNA-DNA hybridization, we first performed a melting temperature (*T*_m_) analysis of WAS-RNA and WAS-BNA-3 annealed to single-strand on-target and off-target DNA templates, in the absence of Cas9. This experiment revealed slight differences in duplex stability, as *T*_m_ values for WAS-BNA-3 duplexes were higher than those of WAS-RNA (Supplementary Fig. 11). Since the differences in *T*_m_ values between WAS-RNA and WAS-BNA-3 crRNA-DNA heteroduplexes were nearly equivalent for on-target and off-target sequences, we employed a previously described single-molecule fluorescence resonance energy transfer (smFRET) assay to monitor changes in hybridization between the crRNA and target DNA in the presence of Cas9. This assay employs a Cy5-labeled crRNA complexed to Cas9 and a Cy3-labeled DNA substrate immobilized on a quartz surface^44^. Briefly, changes in FRET occurring between the Cy3-Cy5 dye pair during R-loop formation represent the transitional dynamics between the partially zipped (“open”; low-FRET) and the high fully zipped (“zipped”; high-FRET) sub-conformations of the Cas9 complex, which correspond to an intermediate and cleavage-competent state, respectively (Fig. 4a)^44^. We used this technology to measure the relative ratio of Cas9 complexes in each state using either WAS-RNA or WAS-BNA-3 in conjunction with the on-target sequence (WAS), or an off-target sequence (WAS-OT4). For the on-target sequence, we found that most molecules were populated in the zipped conformation for both WAS-RNA and WAS-BNA-3 crRNAs, while a drastic decrease in the proportion of molecules in the zipped conformation was observed for WAS-BNA-3, relative to WAS-RNA, when the off-target sequence was used (Fig. 4b). This result is consistent with the specificity improvements observed using WAS-BNA-3 in vitro and in cells. To examine differences in population kinetics between complexes containing either WAS-RNA or WAS-BNA-3, we analyzed single-molecule time trajectories (Fig. 4c). In accordance with the FRET histograms, trajectories for the on-target substrate were mainly docked in the zipped conformation regardless of BNA^NC^ incorporation (Supplementary Fig. 12a). For the off-target DNA, however, the majority of molecules exhibited repetitive transitions between the two sub-conformations (Supplementary Fig. 12b). We measured the dwell time in each state and found that the time spent in both the open and the zipped conformation was reduced by half using WAS-BNA-3 crRNA, compared to the unmodified crRNA (Fig. 4d). This implies that BNA^NC^-modified crRNAs accelerate the dynamic transitions on the off-target substrate, leading to a shortened dwell-time in the cleavage-competent zipped conformation and a reduction of off-target cleavage. Because these findings suggest that BNA^NC^-modified crRNAs may alter Cas9 enzyme kinetics, we performed several time course experiments using an in vitro assay to compare rates of cleavage between WAS-BNA-3, WAS-BNA-2, and EMX1-BNA-5, and their unmodified crRNA counterparts. We found that on-target DNA cleavage was slower using BNA^NC^-modified crRNAs compared to the unmodified crRNAs in all instances where specificity was improved (Supplementary Figs. 13–15). Taken together, these results suggest that the specificity improvements imparted by BNA^NC^-modified crRNAs likely stem from delayed reaction kinetics coupled to an impaired ability to form a productive zipped conformation, a prerequisite for DNA cleavage^44^, on off-target sequences.

### Incorporation of LNAs into crRNAs improves Cas9 specificity

To examine if the Cas9 specificity improvements imparted by BNA^NC^-modified crRNAs are conferred by a general property of BNAs, or if they are unique to these particular nucleotides, we substituted BNA^NC^ modifications in three of the WAS and EMX1 crRNAs (WAS-BNA-3/5/6 and EMX1-BNA-5/6/7) with LNA modifications. Like BNA^NC^-modified crRNAs, WAS, and EMX1-directed LNA-modified crRNAs (WAS-LNA-3/5/6 and EMX1-LNA-5/6/7) induced less off-target cleavage by Cas9 compared to unmodified crRNAs in vitro (Supplementary Fig. 16, Supplementary Table 6). In addition, high-throughput specificity profiling revealed that Cas9 specificity is broadly improved using WAS-LNA-3 and EMX1-LNA-5, compared to the corresponding unmodified crRNAs (Supplementary Figs. 17, 18 and Supplementary Table 4). However, while specificity enhancements between EMX1-BNA-5 and EMX1-LNA-5 were comparable, in vitro DNA cleavage by Cas9 was substantially more promiscuous with WAS-LNA-3 than with WAS-BNA-3 (Supplementary Figs. 16–18). These results were mirrored in HeLa and U2OS cells. For example, while WAS-LNA-3 dramatically reduced cleavage of WAS-OT5 by >23,000-fold vs. WAS-RNA, it had lesser or no effect on WAS-OT2, WAS-OT3, and WAS-OT4, in stark contrast to WAS-BNA-3 (Table 1, Supplementary Table 5, and Supplementary Table 7). The distribution of indel size and location produced by Cas9 DNA cleavage was unaffected by LNA incorporation (Supplementary Figs. 19–21). Moreover, both Cas9 activity and enzyme kinetics were reasonably similar (activity was slightly reduced) using either WAS-LNA-3 or WAS-RNA (Supplementary Fig. 22). Overall, these data establish that LNA substitutions at specific locations in crRNAs improve Cas9 specificity in vitro and in cells, although to a lesser extent than the corresponding BNA^NC^ substitutions.

## Discussion

Crystal structures have revealed that Cas9 forms seven hydrogen bonds with ribose 2′ hydroxyl groups in crRNA nucleotides, four of which occur within the guide sequence^27,45^. Remarkably, RNA nucleotides at all locations within the guide segment can be recoded as DNA nucleotides, with the exception of position 16^27^. Moreover, up to 70% of ribose sugars in sgRNAs can be chemically modified with 2′ methoxy or 2′ fluoro moieties without disruption of Cas9 activity^28^. Consistent with these findings, our results demonstrate that Cas9 is highly tolerant toward incorporation of small stretches of 1–4 BNA^NC^ and LNA modifications throughout the crRNA guide segment. While a few of the modified crRNAs we generated showed low activity in vitro and in cells, such as EMX1-BNA-2 and EMX1-BNA-9 (Figs. 1, 3 and Supplementary Fig. 1), we speculate that this was caused by mis-folding due to stabilization of secondary structures by BNA^NC^ modifications^24^.

Surprisingly, our results indicate that incorporation of BNA^NC^ or LNA nucleotides within the central region (positions 10–14) of crRNAs substantially increases specificity in the PAM-proximal and PAM-distal regions (Figs. 1, 2 and Supplementary Figs. 1–4, 16–18). This is distinct from previous work showing that inclusion of LNAs in isolated DNA–DNA duplexes enhances mismatch discrimination locally^24^. Examination of these central nucleotides in the Cas9 complex crystal structure reveals that they do not make robust contacts with the Cas9 enzyme (Supplementary Fig. 23)^46^. Therefore, we speculate that BNA^NC^ incorporation alters how crRNAs hybridize with the target DNA sequence. A conformational mechanism is supported by our biophysical data showing that BNA^NC^ incorporation facilitates repetitive transitions of the Cas9 complex between the open and zipped conformations on off-target sequences (Fig. 4 and Supplementary Fig. 12), possible due to the destabilization of either states. Based on our *T*_m_ experiments (Supplementary Fig. 11), we theorize that BNA^NC^ or LNA substitutions stabilize stacking to their corresponding DNA bases (increased relative to RNA), but destabilize hybridization in adjacent positions, in the context of Cas9. Structural studies have shown that the crRNA “seed” region, positions ~9–20 in the crRNA^10^, is bound by Cas9 as a pre-ordered A-form helix, while the PAM-distal portion of the crRNA is maintained in a disordered state^10^. Since our most specific BNA^NC^ and LNA-modified crRNAs contain substitutions in positions 10–14, which overlap with the terminal bases in the seed sequence, it is possible that they promote an extended A-form structure throughout the entirety of the crRNA that alters DNA target hybridization. This is consistent with our observations that BNA^NC^ or LNA substitutions occurring early in the seed sequence (near the PAM), or distal to it, have less effect on overall specificity (Figs. 1, 2 and Supplementary Figs. 1–4, 16–18).

Our data indicates that incorporation of BNA^NC^ nucleotides into crRNAs is generally more effective than incorporation of LNA nucleotides at the same positions for improving Cas9 cleavage specificity (Figs. 1–3, Tables 1–2, Supplementary Figs. 16–21, and Supplementary Table 7). Since LNA bases are in fact more conformationally restricted then BNA^NC^ bases^31^, it is unlikely that the specificity improvements rely solely on the degree of conformational constraint of the nucleic acid. Rather, our results suggest that other features of BNA^NC^ bases, such as their large steric bulk or chemical substituents likely contribute to improving mismatch discrimination. Previous work has reported that the nitrogen atom within BNA^NC^ bases can directly influence repulsion between negatively charged phosphate backbones on opposing DNA strands^31^. Furthermore, the increased steric bulk of BNA^NC^ substitutions vs. LNA substitutions in crRNAs could magnify the relevant conformational changes that improve specificity. Subsequent crystallographic studies will be needed to elucidate these details.

Tight target engagement is required for Cas9 DNA cleavage in cells^27^. In agreement with our in vitro data, we found that incorporation of BNA^NC^ and LNA nucleotides at central positions in crRNAs dramatically improves Cas9 cleavage specificity in cells. However, we also noted that the Cas9 on-target modification frequency of BNA^NC^-modified crRNAs in cells was ~2–3-fold lower than their unmodified counterparts (Tables 1–2 and Supplementary Table 5), in contrast to the more equal activity observed in vitro (Fig. 1 and Supplementary Fig. 1). This discrepancy is likely due to differences in reaction kinetics that manifest more apparently in a cellular context. For example, because Cas9 on-target cleavage is significantly delayed using BNA^NC^-modified crRNAs (Supplementary Fig. 13) the probability of the Cas9 complex being ejected from DNA by cellular factors prior to inducing cleavage would be increased. This assertion is supported by our observation that LNA-modified crRNAs, whose kinetics are less altered in vitro (Supplementary Fig. 22), induce on-target modification rates in cells closer to their unmodified crRNA counterparts (Supplementary Tables 5 and 7). A recent study proposed that crRNA/DNA target hybridization kinetics plays a key role in Cas9 off-target discrimination^47^, which could explain the enhanced specificity of BNA^NC^ substitutions compared to LNA substitutions. This assertion could be evaluated in future studies by using a protein evolution platform such as DNA-binding phage-assisted continuous evolution^41^ to evolve Cas9 variants that bypass the BNA^NC^-induced kinetic block.

Here, we show that incorporation of BNA^NC^ and LNA bases at specific positions within crRNAs broadly improves Cas9 DNA cleavage specificity in vitro and in cells. Furthermore, we show that these modified crRNAs enhance specificity by impairing the formation of the stable “zipped” conformation during hybridization to off-target sequences. Overall, these findings unveil a strategy for improving the specificity of the CRISPR-Cas9 system and illustrate the application of recently developed synthetic nucleic acid technologies to solving problems in enzyme specificity. We anticipate that these findings will directly contribute to the ongoing goal of improving the specificity and safety of genome-editing agents for a wide variety of experimental and clinical applications.

## Methods

### Chemical reagents and oligonucleotides

All chemicals were purchased from Sigma-Aldrich. DNA oligonucleotides and tracrRNA were purchased from Integrated DNA Technologies (IDT). Unmodified crRNAs and crRNAs containing BNAs (BNA^NC^[N-Me]) were obtained from BioSynthesis Inc., while crRNAs containing LNAs were purchased from Exiqon. eSpCas9 endonuclease was purchased from Sigma-Aldrich. Sequences of crRNAs and tracrRNAs used in this study are listed in Supplementary Table 8. Sequences of DNA oligonucleotide used in this study are listed in Supplementary Table 9.

### Cloning and plasmid construction

Plasmid templates for in vitro cleavage assays were generated through ligation of inserts into *HindIII* and *XbaI* double-digested pUC19 (ThermoFisher). DNA encoding Cas9 target sites was purchased either as a gBlock from IDT (for genomic on-targets and off-targets), or as ssDNA for off-target sequences with single-nucleotide mismatches, as listed in Supplementary Table 9. Forward and reverse ssDNA oligonucleotides containing single-nucleotide mismatch target sites were heated to 95 °C for 5 min, then cooled to 25 °C over the course of 1 h to assemble dsDNA prior to ligation. pET-NLS-Cas9-6xHis was purchased from Addgene (#62934) and used to express Cas9 protein for all in vitro cleavage assay experiments. pET-NLS-dCas9-6xHis (D10A/H840A) was generated using the Q5 Site Directed Mutagenesis Kit (NEB), according to the manufacturer’s instructions.

### Expression and purification of *S. pyogenes* Cas9

Recombinant Cas9 was prepared and purified as previously described^40^. Briefly, *E. coli* Rosetta 2 cells were transformed with a plasmid encoding the *S. pyogenes Cas9* gene fused to an N-terminal 6xHis-tag and NLS (Addgene #62934). Transformed bacteria were used to inoculate 5 mL of LB broth containing 50 µg mL^−1^ carbenicillin, and incubated at 37 °C overnight (~16 h). The next day, cells were diluted 1:100 into the same growth medium and grown at 37 °C until an OD_600_ of 0.6 was reached. The culture was incubated at 16 °C for 30 min after which point isopropyl-ß-D-1-thiogalactopyranoside was added to a final concentration of 0.5 mM to induce Cas9 expression. After 16 h, cells were collected by centrifugation for 15 min at 2700 × *g* and re-suspended in lysis buffer (20 mM Tris-Cl, pH 8.0, 250 mM NaCl, 5 mM imidazole, pH 8.0, 1 mM PMSF). The solution was incubated on ice for 30 min before proceeding. Cells were further lysed by sonication (30 s pulse-on and 60 s pulse-off for 7.5 min at 60% amplitude) with soluble lysate being obtained by centrifugation at 30,000 × *g* for 30 min. The cell lysate containing Cas9 was injected into a HisTrap FF Crude column (GE Healthcare) attached to an AKTA Start System (GE Healthcare) and washed with wash buffer (20 mM Tris-Cl, pH 8.0, 250 mM NaCl, 10 mM imidazole, pH 8.0) until UV absorbance reached a baseline. Cas9 was eluted in elution buffer (20 mM Tris-Cl, pH 8.0, 250 mM NaCl, 250 mM imidazole, pH 8.0) in a single step. Eluted Cas9 was exchanged into storage buffer (20 mM HEPES-KOH, pH 7.5, 500 mM NaCl, 1 mM DTT) during concentration using a 100 kDa centrifugal filter (Pall). Concentrated Cas9 was flash-frozen in liquid nitrogen and stored in aliquots at −80 °C. dCas9 was purified in a similar manner.

### In vitro cleavage of on-target and off-target DNA substrates

Primers pUC19_fwd and pUC19_rev (listed in Supplementary Table 9) were used to generate Cas9 substrate DNAs through PCR amplification of previously prepared plasmid templates and subsequently purified with the QIAquick PCR Purification Kit (Qiagen). Equimolar amounts of tracrRNA (IDT) and crRNA (BioSynthesis) were heated at 95 °C for 10 min, then cooled to 25 °C over the course of 1 h to prepare gRNAs. gRNAs containing BNA^NC^-modified and LNA-modified crRNAs were prepared as described above. For each cleavage reaction, 5 nM of substrate DNAs were incubated with 150 nM, or 15 nM pre-assembled Cas9 RNP complex for 1 h at 37 °C in Cas9 cleavage buffer (5% glycerol, 0.5 mM EDTA, 1 mM DTT, 2 mM MgCl_2_, 20 mM HEPES pH 7.5, 100 mM KCl). Reactions were halted by purifying the products using the MinElute PCR Purification Kit (Qiagen). Cleavage products were resolved on a 1% agarose gel, and imaged on an Amersham Imager 600 (GE Healthcare). Cleavage assays using eSpCas9 (Sigma-Aldrich) were performed as described above.

### Library for high-throughput specificity profiling

Generation of pre-selection libraries for in vitro high-throughput specificity profiling experiments were performed as previously described^40^. Briefly, 10 pmol of WAS or EMX1 lib oligonucleotides were circularized through incubation with 100 units of CircLigase II ssDNA Ligase (Epicenter) in a total reaction volume of 20 µL for 16 h at 60 °C in 1× CircLigase II Reaction Buffer. The reaction was heat inactivated by incubation at 85 °C for 10 min. 5 pmol of the crude circular ssDNA was converted into concatemeric pre-selection libraries with the illustra TempliPhi Amplification Kit (GE Healthcare) according to the manufacturer’s protocol. Concatemeric pre-selection libraries were quantified with the Qubit 2.0 Fluorometer. Sequences used to generate in vitro pre-selection libraries are listed in Supplementary Table 9.

### In vitro high-throughput specificity profiling

High-throughput specificity profiling of unmodified, BNA^NC^-modified and LNA-modified crRNAs was performed as previously described^40^. Briefly, 200 nM of concatemeric pre-selection libraries were incubated with 1000 nM Cas9 and 1000 nM gRNA or 100 nM Cas9 and 100 nM gRNA in Cas9 cleavage buffer (NEB) for 20 min at 37 °C. Pre-selection libraries were also separately incubated with 2 U of BspMI (NEB) in NEBuffer 3.1 for 1 h at 37 °C. Cas9-digested and BspMI-digested library members were purified with the QiaQuick PCR Purification Kit (Qiagen) and ligated to 10 pmol adaptor1/2(#) (post-selection) or lib adapter 1/lib adapter 2 (pre-selection) (sequences in Supplementary Table 9) with 1000 U of T4 DNA Ligase (NEB) in NEB T4 DNA Ligase Reaction Buffer for 16 h at room temperature. Adapter ligated DNA was purified using the QiaQuick PCR Purification Kit (Qiagen) and PCR amplified for 19–24 cycles with Q5 Hot Start High-Fidelity DNA Polymerase (NEB) in Q5 Reaction Buffer using primers PE2 short/sel PCR (post-selection) or primers lib seq PCR/lib fwd PCR (pre-selection) (sequences in Supplementary Table 9). PCR products were gel purified and quantified using a Qubit 2.0 Fluorometer (ThermoFisher) and subject to single-read sequencing on an Illumina MiSeq. Pre-selection and post-selection sequencing data were analyzed as previously described^40^.

### Electrophoretic mobility shift assay

EMSAs were performed as previously described^48^, with minor modifications to the protocol. To prepare the 6-FAM-labeled dsDNA substrate, EMSA fwd and rev oligonucleotides (listed in Supplementary Table 9) were mixed in a 1.5:1 molar ratio, incubated at 95 °C for 5 min, then cooled to 25 °C over the course of 1 h. DNA substrates were diluted to a working concentration of 200 nM in binding buffer (20 mM HEPES, pH 7.5, 250 mM KCl, 2 mM MgCl_2_, 0.01% Triton X-100, 0.1 mg mL^−1^ bovine serum albumin, 10% glycerol). gRNAs were prepared as described for in vitro cleavage assays. Next, nuclease-deficient Cas9 (dCas9) was incubated with previously annealed gRNA in a 1:1 molar ratio for 10 min at 25 °C in binding buffer to form the RNP complex. 50 nM DNA substrate was incubated with 0, 10, 50, 100, 250, and 500 nM RNP for 10 min at 37 °C in binding buffer. Reactions were resolved on a 10% TBE polyacrylamide gel supplemented with 2 mM MgCl_2_ in 1× TBE buffer supplemented with 2 mM MgCl_2,_ and imaged on a Typhoon laser gel scanner (GE Healthcare). EMSAs using BNA^NC^-modified crRNAs were performed in a similar manner.

### Determination of crRNA–DNA heteroduplex melting temperature

Equimolar amounts of crRNA and complementary ssDNA oligonucleotide (listed in Supplementary Table 9) were mixed in Duplex Buffer (30 mM HEPES, pH 7.5, 100 mM Potassium Acetate) (IDT) to a final concentration of 2 µM. SYBR Green I was added to a final concentration of 1×. The solution was moved to a CFX96 Real-Time System (BioRad) and incubated for 5 min at 95 °C, then cooled to 25 °C at 0.1 °C s^−1^ to anneal the RNA/DNA heteroduplex. The heteroduplex was then heated at a rate of 0.1 °C s^−1^ to 95 °C and the corresponding fluorescent signal was used to generate a melt curve.

### Generation of Cas9 stable cells

lentiCas9-Blast viral particles were purchased from Addgene (#52962) and used to infect U2OS and HeLa cells according to the manufacturer’s protocol. Briefly, on the day of infection, cells were trypsinized, counted, and diluted to a working concentration of 50,000 cells mL^−1^ in DMEM media supplemented with 10% FBS/1× pen-strep/1× glutamine (Gibco) (DMEM complete) and 10 µg mL^−1^ polybrene. Viral particles were serially diluted down to 1:500 from the original stock (2.5 × 10^5^ TU mL^−1^), with 500 µL of each dilution added to the corresponding wells of a 6-well plate. 1 mL of cell suspension was added to each well and incubated at 37 °C and 5% CO_2_. 48 h after infection, selection was performed in DMEM-complete media supplemented with 10 µg mL^−1^ Blasticidin S HCl (Gibco). After selection, cells stably expressing Cas9 were maintained in DMEM-complete media with 5 µg mL^−1^ Blasticidin S HCl.

### Cell culture

All cells were cultured at 37 °C in a 5% CO_2_ atmosphere. U2OS-Cas9 and HeLa-Cas9 cells were cultured in high glucose DMEM media with pyruvate (Gibco) supplemented with 10% FBS/1× pen-strep/1× glutamine (Gibco) and 5 µg mL^−1^ Blasticidin S HCl (Gibco), where applicable. The U2OS and HeLa cells were previously authenticated and shown to be negative for mycoplasma at the time of purchase (ATCC).

### Cationic lipid transfection of gRNA into stable cell lines

Cells stably expressing Cas9 were transfected with RNAiMAX and annealed gRNA according to the manufacturers’ instructions to a final concentration of 30 nM. Experiments involving BNA^NC^-modified and LNA-modified crRNAs were performed in a similar fashion.

### Cellular cleavage assay

Genomic DNA (gDNA) from transfected cells was extracted using a DNeasy Kit (Qiagen) 48 h after transfection, according to the manufacturer’s instructions, and quantified using a NanoPhotometer NP80 (Implen) spectrophotometer. Amplicon specific primer pairs (listed in Supplementary Table 9) and 100 ng of gDNA was used to PCR amplify the desired target site, which was then purified with the QIAquick PCR Purification Kit (Qiagen). T7 endonuclease I (T7E1) digestion of the PCR products was performed as described in the manufacturer’s protocol (NEB). Cleavage products were resolved on a 2.5% agarose gel.

### High-throughput sequencing

100 ng gDNA isolated from cells from each transfection was amplified by PCR for 35 cycles with primers PCR1_fwd and PCR1_rev (listed in Supplementary Table 9) and 2× Q5 Hot Start High Fidelity Master Mix according to the manufacturer’s instructions (NEB). PCR products were purified via GeneRead Size Selection Kit (Qiagen). Purified PCR products were then amplified by PCR with Nextera XT Illumina primers for 7 cycles with 2× Q5 Hot Start High Fidelity Master Mix in Q5 Reaction Buffer (NEB), to add unique barcodes. Amplified control and treated DNA pools were subsequently purified with the GeneRead Size Selection Kit (Qiagen), quantified using the Qubit 2.0 Fluorometer (ThermoFisher), and pooled in a 1:1 ratio. The final sample underwent paired-end sequencing using an Illumina MiSeq instrument, according to the manufacturer’s protocol. Statistical analysis and determination of modification frequency was performed using CRISPR-DAV^49^ and Cas-Analyzer^50^.

### Single-molecule FRET measurements

Single-molecule FRET experiments were set up and performed using a previously described protocol^38^. Briefly, to prevent non-specific binding of samples to the glass surface, all coverslips and quartz glasses were passivated using polyethylene glycol. A single-molecule flow chamber consisting of a microscope slide and coverslip sealed with epoxy and double-sided tape was assembled using rounded holes on either side of the slide as inlets and outlets for solution exchange. All imaging was performed at room temperature with the following buffer composition: 100 mM NaCl, 50 mM Tris-HCl, pH 7.9, 10 mM MgCl_2_, 1 mM DTT, 5% glycerol, and 0.1 mg mL^−1^ BSA. For experiments used to generate FRET histograms, the oxygen scavenger (1 mg mL^−1^ of glucose oxidase (Sigma-Aldrich), 0.04 mg mL^−1^ of catalase (Sigma-Aldrich), and 0.8% (w/v) of b-D-glucose), and the triplet quencher (~4 mM Trolox) were applied to the buffer to prevent photo-fatigue of the fluorophores. FRET histograms were obtained from the images 30 min after incubation of Cas9 RNP complex (30 nM Cas9, 10 nM gRNAs) with target containing DNA sequences (listed in Supplementary Table 9). Single-molecule time trace imaging was performed as described above, with the exception of the oxygen scavenging system as well as an addition of 5% (v/v) glycerol. Time traces were acquired throughout the duration of the incubation (from 0 to 15 min) of Cas9 RNP complex with target DNA. The acquisition time for smFRET histograms and time traces were 100 and 30 ms, respectively.

### Statistical analysis

Indel percentages shown in Fig. 3 were calculated as indel (%) = 100 × (1−(1−fraction_cut_)^0.5^). *P-*values in Supplementary Table 4 were calculated through the comparison of 150,000 randomly sampled sequences from the pre-selection library (WAS or EMX1) with an equal number of sequences from the corresponding post-selection library using a Mann–Whitney test. *P*-values in Supplementary Table 5 were calculated using a Fisher exact test comparing crRNA transfected samples with the un-transfected control.

### Code availability

Python scripts used for deep sequencing data processing are available upon request.

### Data availability

Data generated for this work are included in this published article and its associated Supplementary Information files. High-throughput sequencing data files have been deposited in the NCBI SRA database and are available under accession number: SRP125574.

## Electronic supplementary material

Supplementary Information
