# Supplementary material for: Incorporation of bridged nucleic acids into CRISPR RNAs improves Cas9 endonuclease specificity
Source: Nat Commun. 2018 Apr 13;9:1448. doi: 10.1038/s41467-018-03927-0 (PMC5899152; doi:10.1038/s41467-018-03927-0)
Supplement: Supplementary file 1 — Supplementary Information [file 41467_2018_3927_MOESM1_ESM.pdf]

Incorporation of bridged nucleic acids into CRISPR RNAs improves Cas9 endonuclease specificity

Christopher R. Cromwell, Keewon Sung, Jinho Park, Amanda R. Krysler, Juan Jovel, Seong Keun Kim, Basil P. Hubbard

SUPPLEMENTARY INFORMATION

**Supplementary Figure 1**	Incorporation of BNA^{NC} into crRNAs improves Cas9 cleavage specificity *in vitro*.
**Supplementary Figure 2**	Incorporation of BNA^{NC} into crRNAs improves Cas9 cleavage specificity towards off-target sequences with single-nucleotide mismatches *in vitro.*
**Supplementary Figure 3**	Distribution of mutations in pre- and post-selection libraries following *in vitro* library selection with unmodified or BNA^{NC}-modified crRNAs.
**Supplementary Figure 4**	*In vitro* specificity profiling results for unmodified and BNA^{NC}-modified crRNAs.
**Supplementary Figure 5**	BNA^{NC}-modified crRNAs show additive specificity when used in combination with eSpCas9 *in vitro*.
**Supplementary Figure 6**	Comparison of indel size resulting from Cas9 DNA cleavage with unmodified or BNA^{NC}-modified crRNAs.
**Supplementary Figure 7**	Distribution of indel location using unmodified or BNA^{NC}-modified crRNAs on WAS target.
**Supplementary Figure 8**	Distribution of indel location using unmodified or BNA^{NC}-modified crRNAs on EMX1 target.
**Supplementary Figure 9**	Effect of BNA^{NC}-modified crRNAs on affinity, activity, and kinetics of Cas9 *in vitro*.
**Supplementary Figure 10**	Effect of BNA^{NC}-modified crRNAs on the ability of nuclease-deficient Cas9 (dCas9) to bind DNA target sequences.
**Supplementary Figure 11**	Effect of BNA^{NC} modifications on crRNA/DNA melting temperature.
**Supplementary Figure 12**	Kinetic analysis of time spent in open to zipped transition and docked states using single-molecule FRET.
**Supplementary Figure 13**	Cas9 *in vitro* cleavage kinetics using either WAS-RNA or WAS-BNA-3 crRNAs on several target sequences.
**Supplementary Figure 14**	Cas9 *in vitro* cleavage kinetics using either WAS-RNA or WAS-BNA-2 crRNAs on several target sequences.

**Supplementary Figure 15**	Cas9 *in vitro* cleavage kinetics using either EMX1-RNA or EMX1-BNA-5 crRNAs on several target sequences.
**Supplementary Figure 16**	Incorporation of LNA into crRNAs improves Cas9 cleavage specificity *in vitro*.
**Supplementary Figure 17**	Distribution of mutations in pre- and post-selection libraries following *in vitro* library selection with unmodified or LNA-modified crRNAs.
**Supplementary Figure 18**	*In vitro* specificity profiling results for LNA-modified crRNAs.
**Supplementary Figure 19**	Comparison of indel size resulting from Cas9 DNA cleavage with unmodified or LNA-modified crRNAs.
**Supplementary Figure 20**	Distribution of indel location using unmodified or LNA-modified crRNAs on WAS target.
**Supplementary Figure 21**	Distribution of indel location using unmodified or LNA-modified crRNAs on EMX1 target.
**Supplementary Figure 22**	Effect of LNA-modified crRNAs on activity and kinetics of Cas9 *in vitro*.
**Supplementary Figure 23**	Model of Cas9 structure highlighting interactions between crRNA nucleotides 10-14 and Cas9.
**Supplementary Table 1**	*In vitro* cleavage assay data used to generate Fig. 1.
**Supplementary Table 2**	*In vitro* cleavage assay data used to generate Supplementary Fig. 1.
**Supplementary Table 3**	*In vitro* cleavage assay data used to generate Supplementary Fig. 2.
**Supplementary Table 4**	Statistics of sequences from *in vitro* high-throughput library selection experiment.
**Supplementary Table 5**	Statistics of cellular modification frequencies, sample size and *P*-values for high-throughput sequencing of Cas9:gRNA cleavage in U2OS-Cas9 and HeLa-Cas9 cells using no guide RNA, unmodified, BNA^{NC}- or LNA-modified crRNAs.
**Supplementary Table 6**	*In vitro* cleavage assay data used to generate Supplementary Fig. 16.
**Supplementary Table 7**	Cellular modification rates induced by unmodified or LNA-modified crRNAs targeting WAS or EMX1.
**Supplementary Table 8**	Unmodified, BNA^{NC}- and LNA-modified crRNA and tracrRNA sequences used.
**Supplementary Table 9**	Oligonucleotides used in this study.
**Supplementary References**	

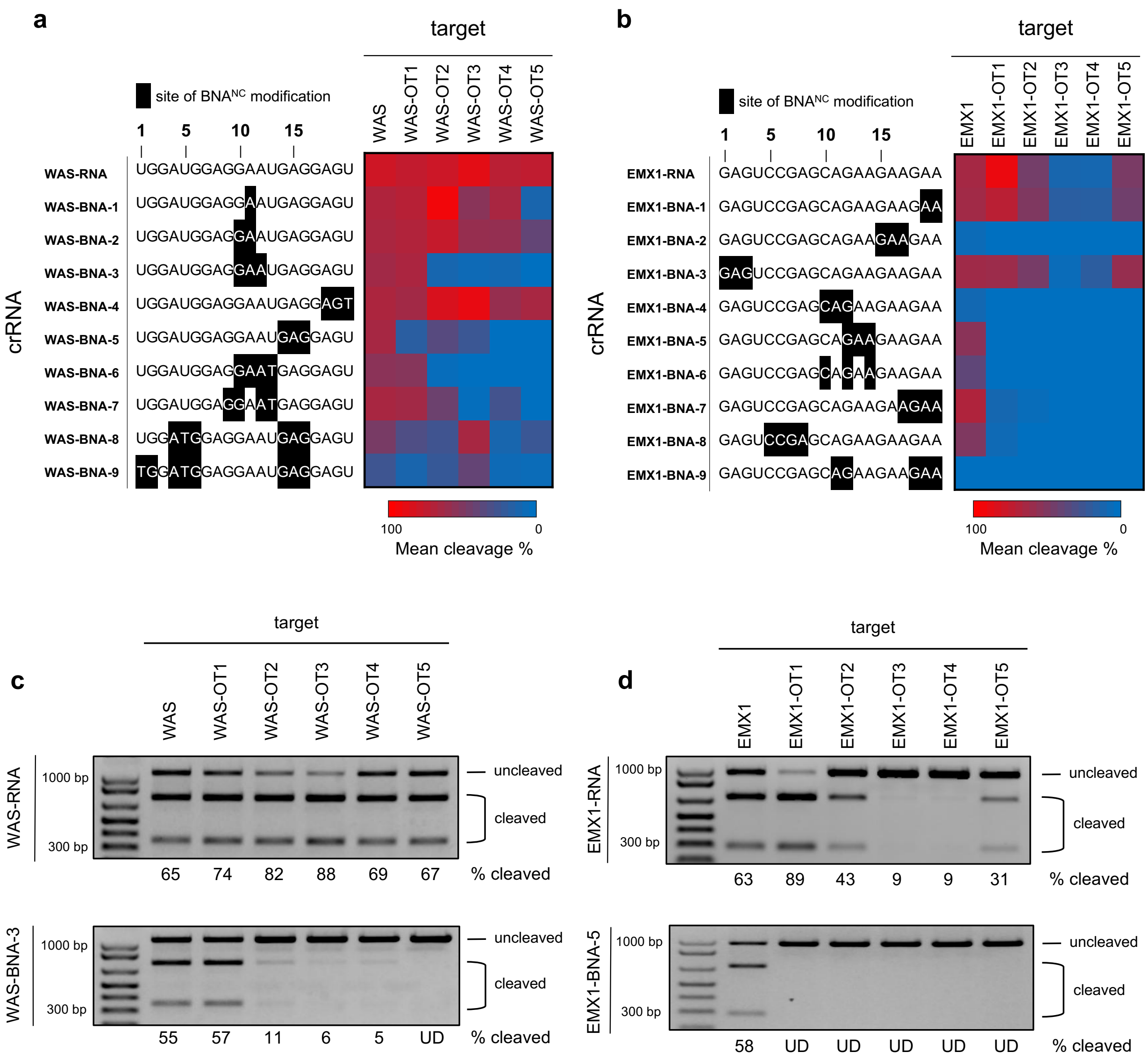

Supplementary Figure 1: Incorporation of BNA^{NC} into crRNAs improves Cas9 cleavage specificity *in vitro*. Heat map showing *in vitro* cleavage specificity for unmodified crRNA and 9 BNA^{NC}-modified crRNAs towards either (**a**) WAS or (**b**) EMX1 on- and off-target sequences (as listed **Fig. 1b**, **c**); Mean shown (n = 2). crRNA and BNA^{NC}-modified sequences are shown to the left of the corresponding heat map. BNA^{NC} modifications are indicated in black. Targets that were highly cleaved *in vitro* are indicated in red, while targets that were not cleaved are indicated in blue. Gel showing relative

cleavage efficiencies of the unmodified and most specific BNA^{NC}-modified crRNAs on a linear 1-kb DNA fragment containing either the (**c**) WAS or (**d**) EMX1 on- and off-target sequences. The two bottom bands are cleavage products, while the top band is full-length substrate. The molar ratio of Cas9 RNP complex to target DNA was 3:1 for these experiments. Quantification of cleavage percentages was determined using densitometry (ImageJ), and are shown below each lane. Lanes in which no cleavage products were observed are marked as undetected (UD). Values used to generate heatmaps are shown in **Supplementary Table 2**.

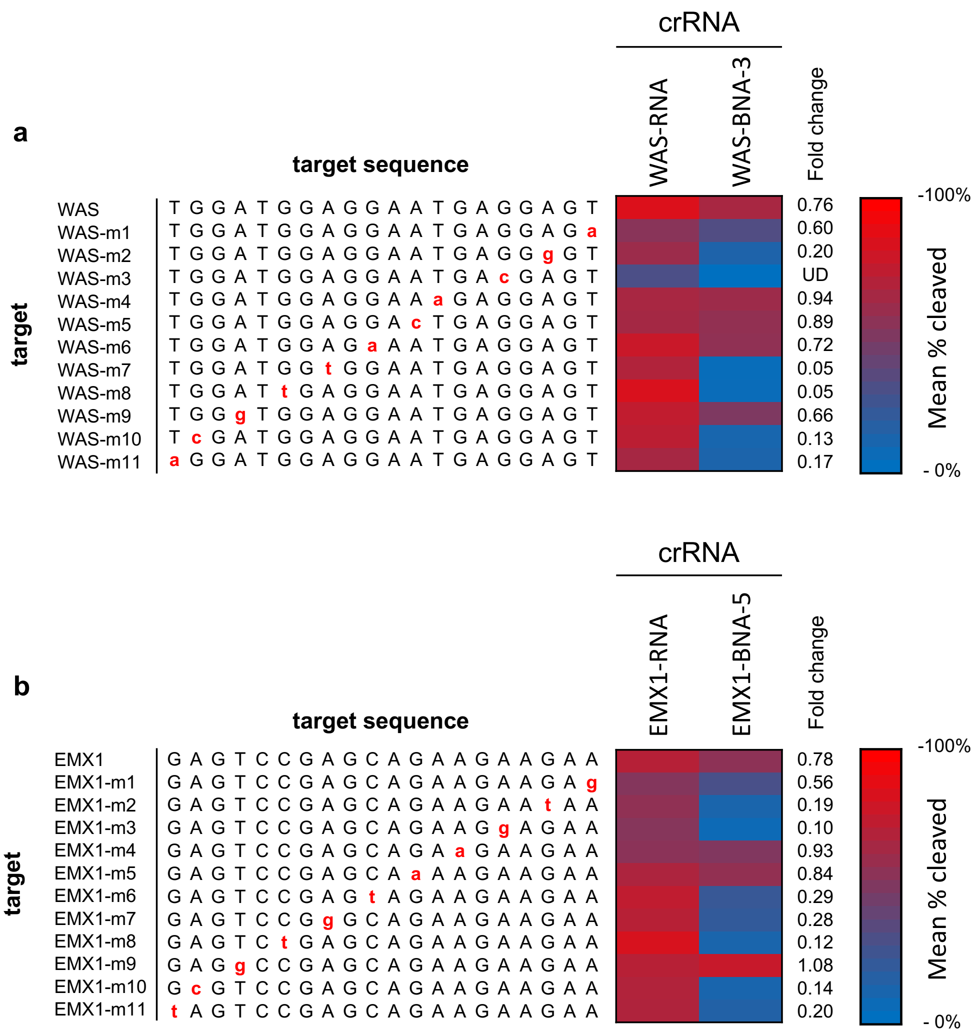

Supplementary Figure 2: Incorporation of BNA^{NC} into crRNAs improves Cas9 cleavage specificity towards off-target sequences with single-nucleotide mismatches *in vitro*. (**a**) Heat map showing cleavage specificity of WAS-RNA and WAS-BNA-3 crRNAs towards on- and off-target sequences containing single-nucleotide mismatches; Mean shown (n = 2). Fold change indicates percent cleavage of WAS-BNA-3/WAS-RNA for each target. (**b**) Heat map showing cleavage specificity of EMX1-RNA and EMX1-BNA-5 crRNAs towards on- and off-target sequences with single-nucleotide mismatches; Mean shown (n = 2). Nucleotide mismatches are indicated by red lowercase lettering. Targets which were highly cleaved *in vitro* are indicated in red, while targets

which were not cleaved are indicated in blue. Fold change indicates percent cleavage of EMX1-BNA-5/EMX1-RNA for each target. A designation of UD indicates no detectable cleavage of the target *in vitro*. Values used to generate the above heatmaps are shown in **Supplementary Table 3**.

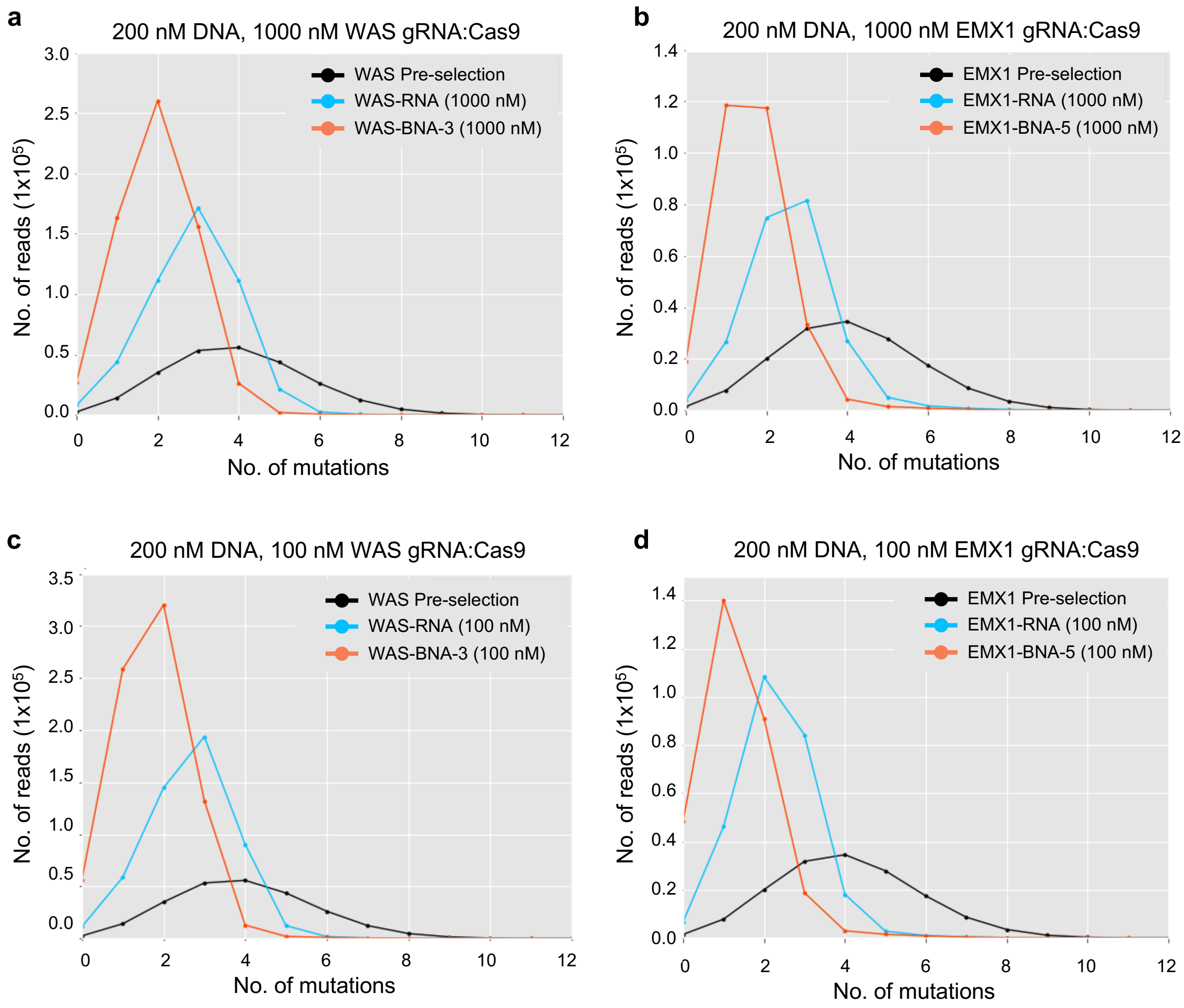

Supplementary Figure 3: Distribution of mutations in pre- and post-selection libraries following *in vitro* library selection with unmodified or BNA^{NC}-modified crRNAs. *In vitro* selections were performed using 200 nM pre-selection library with 1000 nM Cas9 RNP complex targeting either (**a**) WAS or (**b**) EMX1. *In vitro* selections were performed using 200 nM pre-selection library with 100 nM Cas9 RNP complex targeting either (**c**) WAS or (**d**) EMX1. Cas9 RNP complexes were pre-assembled using unmodified or BNA^{NC}-modified crRNAs prior to DNA digestion. Distributions of mutations within pre-selection (black) and post-selection (colored) libraries are shown. Mutations are counted

from the 20-base-pairs comprising the Cas9 target site, as well as the 2-base-pair PAM (N of NGG is excluded). Additional statistics are shown in **Supplementary Table 4**.

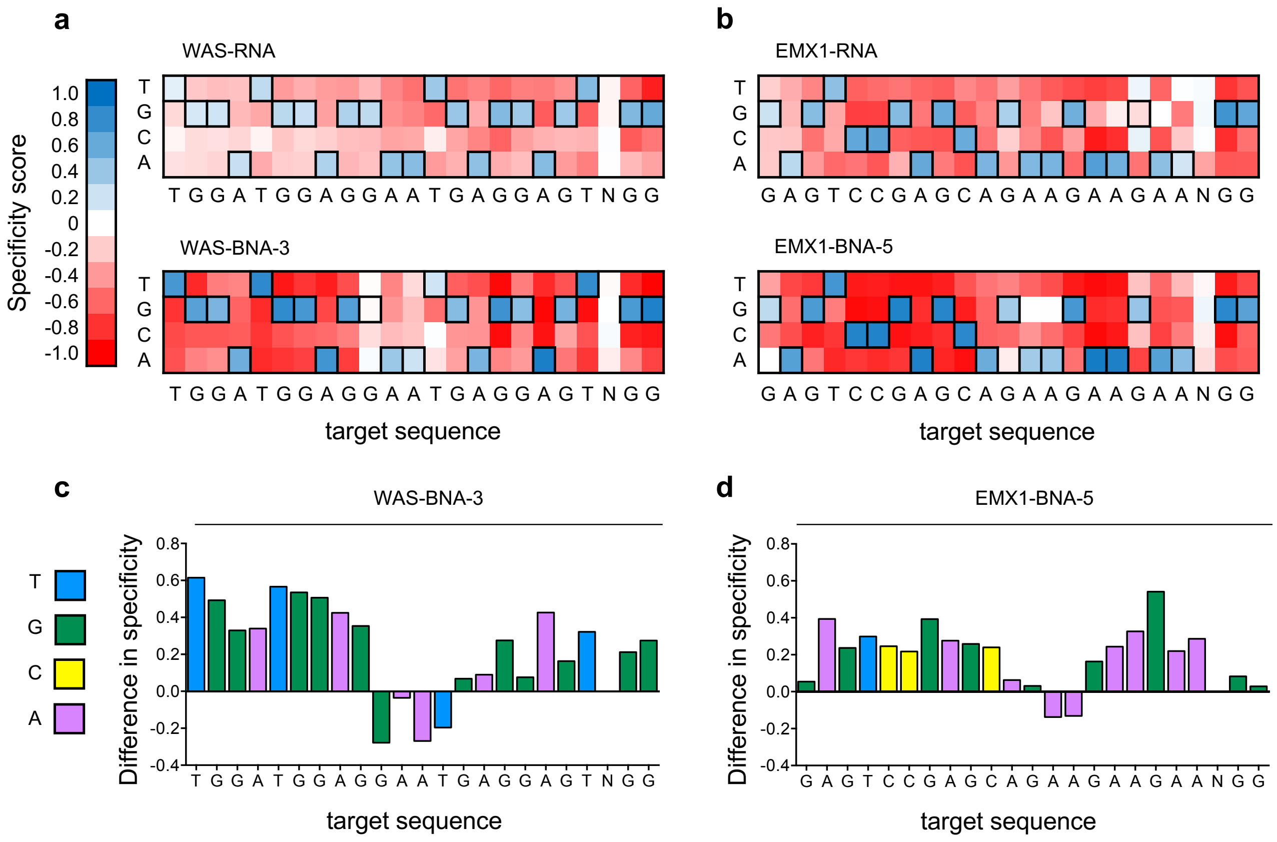

Supplementary Figure 4: *In vitro* specificity profiling results for unmodified and BNA^{NC}-modified crRNAs. Heat maps showing DNA cleavage specificity scores across >10^{12} off-target sequences for either unmodified (top) or BNA^{NC}-modified (bottom) crRNAs targeting (**a**) WAS or (**b**) EMX1. Specificity scores of 1.0 (dark blue) correspond to 100% enrichment for, while scores of -1.0 (dark red) correspond to 100% enrichment against a specific base-pair at a specific position. Black boxes denote the intended target nucleotides. Bar graph showing the quantitative difference in specificity score at each position in the 20 base-pair target site and 2 base-pair PAM (N of NGG excluded), between the unmodified and BNA^{NC}-modified crRNA for (**c**) WAS or (**d**) EMX1 target sequences. A score of zero indicates no change in specificity. Difference in specificity

was calculated as, specificity $score_{BNA}^{NC}$ – specificity $score_{RNA}$. Experiments were performed with 200 nM pre-selection library and 100 nM Cas9 RNP complex.

a

	SpCas9		eSpCas9	
	WAS-RNA	WAS-BNA-5	WAS-RNA	WAS-BNA-5
WAS	81 ± 15	66 ± 10	64 ± 22	61 ± 3
WAS-OT1	77 ± 5	17 ± 6	88 ± 13	8 ± 2
WAS-OT2	81 ± 5	34 ± 7	94 ± 2	UD
WAS-OT3	89 ± 10	23 ± 6	5 ± 2	UD
WAS-OT4	73 ± 6	UD	42 ± 15	UD
WAS-OT5	76 ± 11	UD	7 ± 3	UD

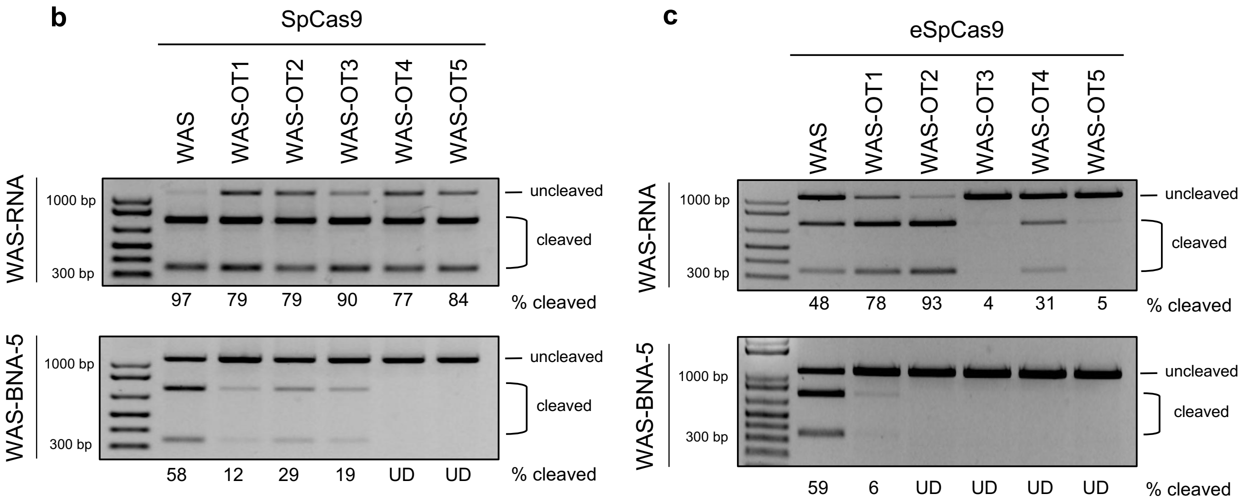

Supplementary Figure 5: BNA^{NC}-modified crRNAs show additive specificity when used in combination with eSpCas9 *in vitro*. (**a**) Table showing cleavage specificities of WAS-RNA and WAS-BNA-5 crRNA towards WAS on- and off-target sequences (shown in **Fig. 1b**) when complexed with either SpCas9 or eSpCas9; Mean ± SD shown (n = 2). Reactions in which no cleavage products were observed are marked as undetected (UD). Gel showing cleavage assay results for WAS-RNA and WAS-BNA-5 crRNA complexed with (**b**) SpCas9 or (**c**) eSpCas9 using WAS on- and off-target containing sequences. For all cleavage gels, the top band is full-length DNA substrate, while the two bottom bands are cleavage products. The molar ratio of Cas9 RNP complex to target DNA was 3:1 for these experiments. Quantification of cleavage products was determined by densitometry

(ImageJ), and are shown below each lane. Lanes in which no cleavage products were observed are marked as undetected (UD).

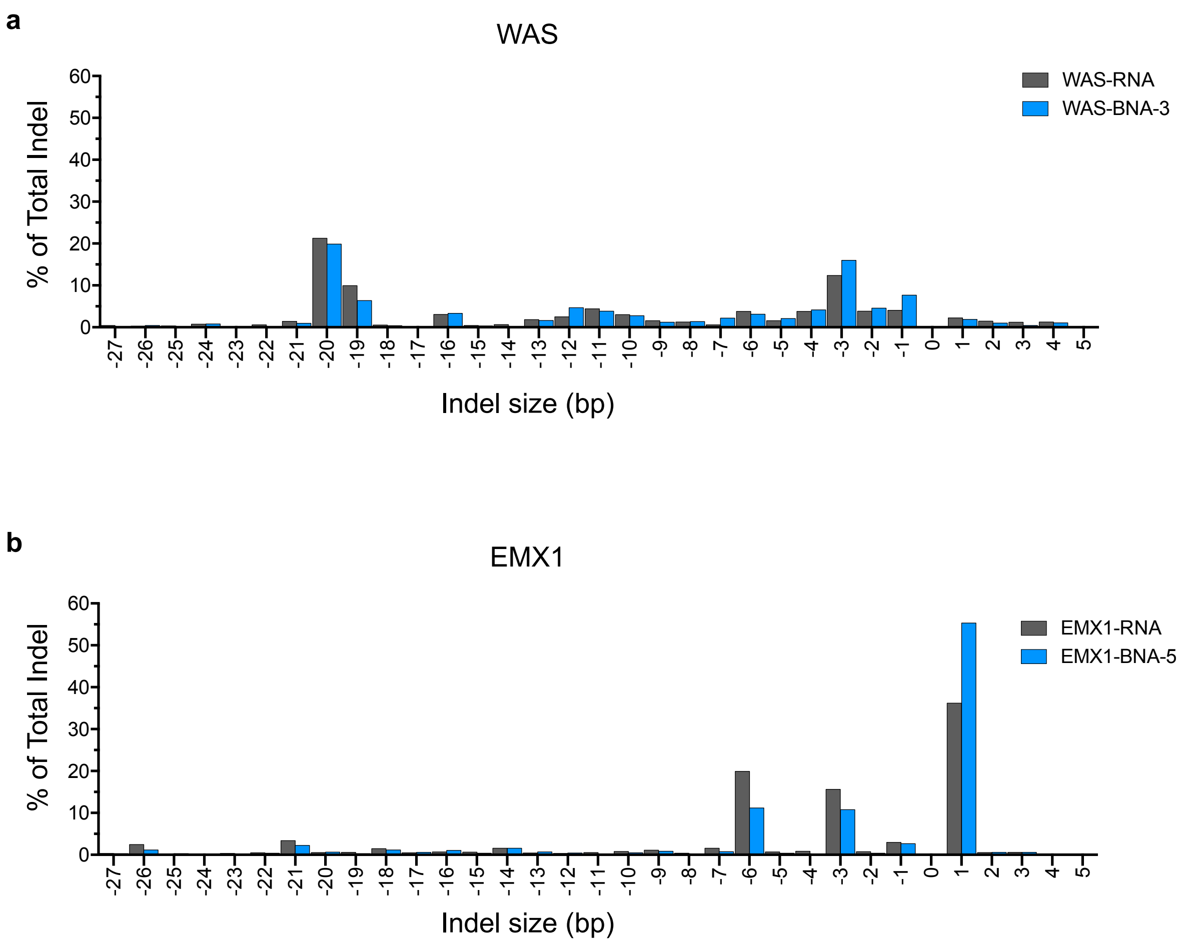

Supplementary Figure 6. Comparison of indel size resulting from Cas9 DNA cleavage with unmodified or BNA^{NC}-modified crRNAs. Distributions of indel sizes are shown for either (**a**) WAS or (**b**) EMX1 using unmodified (grey) or BNA^{NC}-modified (blue) crRNAs in U2OS-Cas9 cells. Distribution of indel sizes is based on sequences obtained following high-throughput sequencing of transfected cells. The number of reads for each indel size was normalized to the total number of indel reads. Only indels ranging from -27 bp (27 bp deletion) to +5 bp (5 bp insertion) are shown.

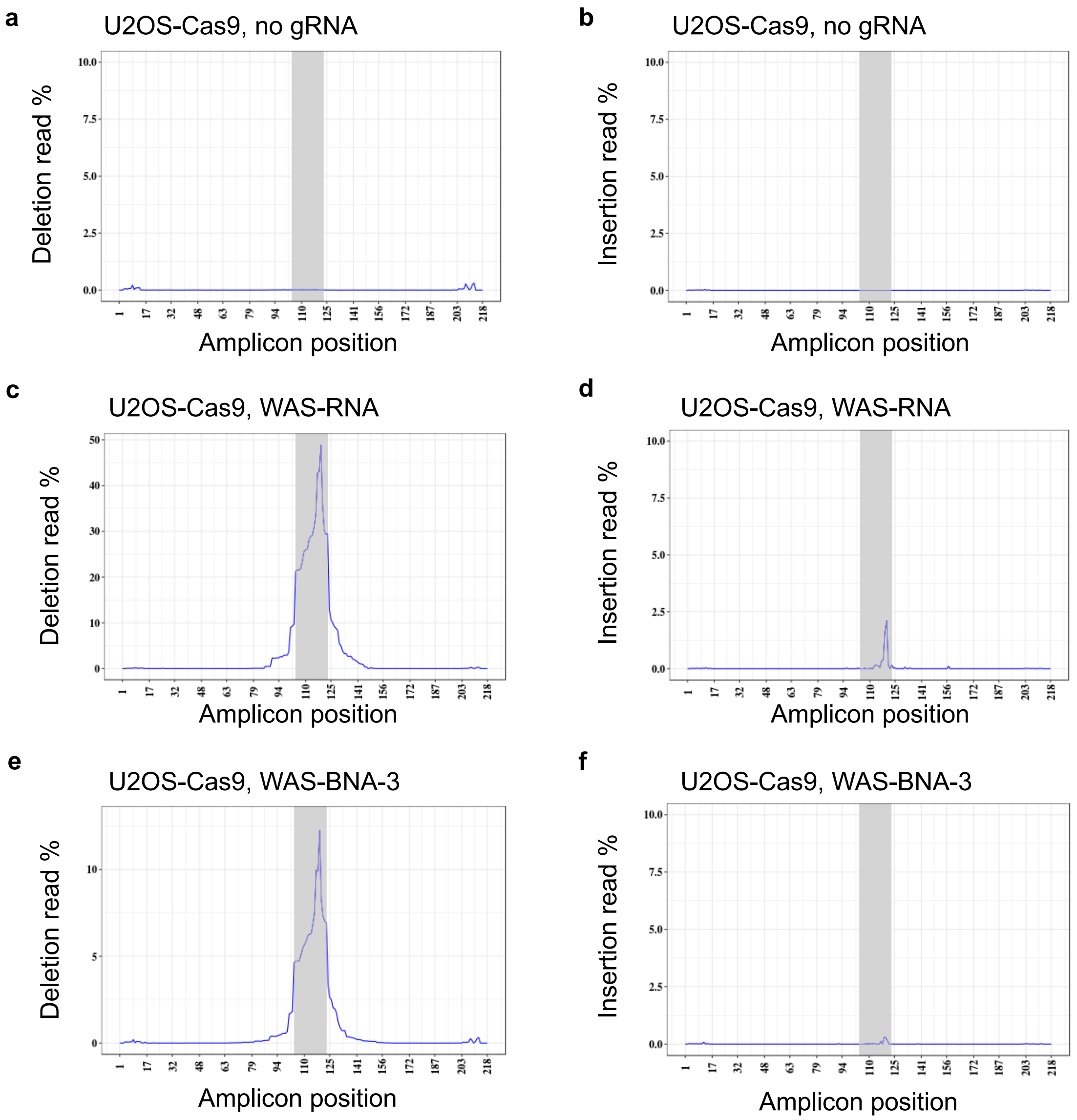

Supplementary Figure 7. Distribution of indel location using unmodified or BNA^{NC}-modified crRNAs on WAS target. Distribution frequencies of insertions (right) and deletions (left) across the *WAS* target site in U2OS-Cas9 cells transfected with no gRNA (**a**, **b**), WAS-RNA (**c**, **d**) or WAS-BNA-3 (**e**, **f**) are shown. Grey coloring indicates the location of the genomic CRISPR target site along the amplicon. Distribution of indel positions is based on sequences obtained following high-throughput sequencing of transfected cells.

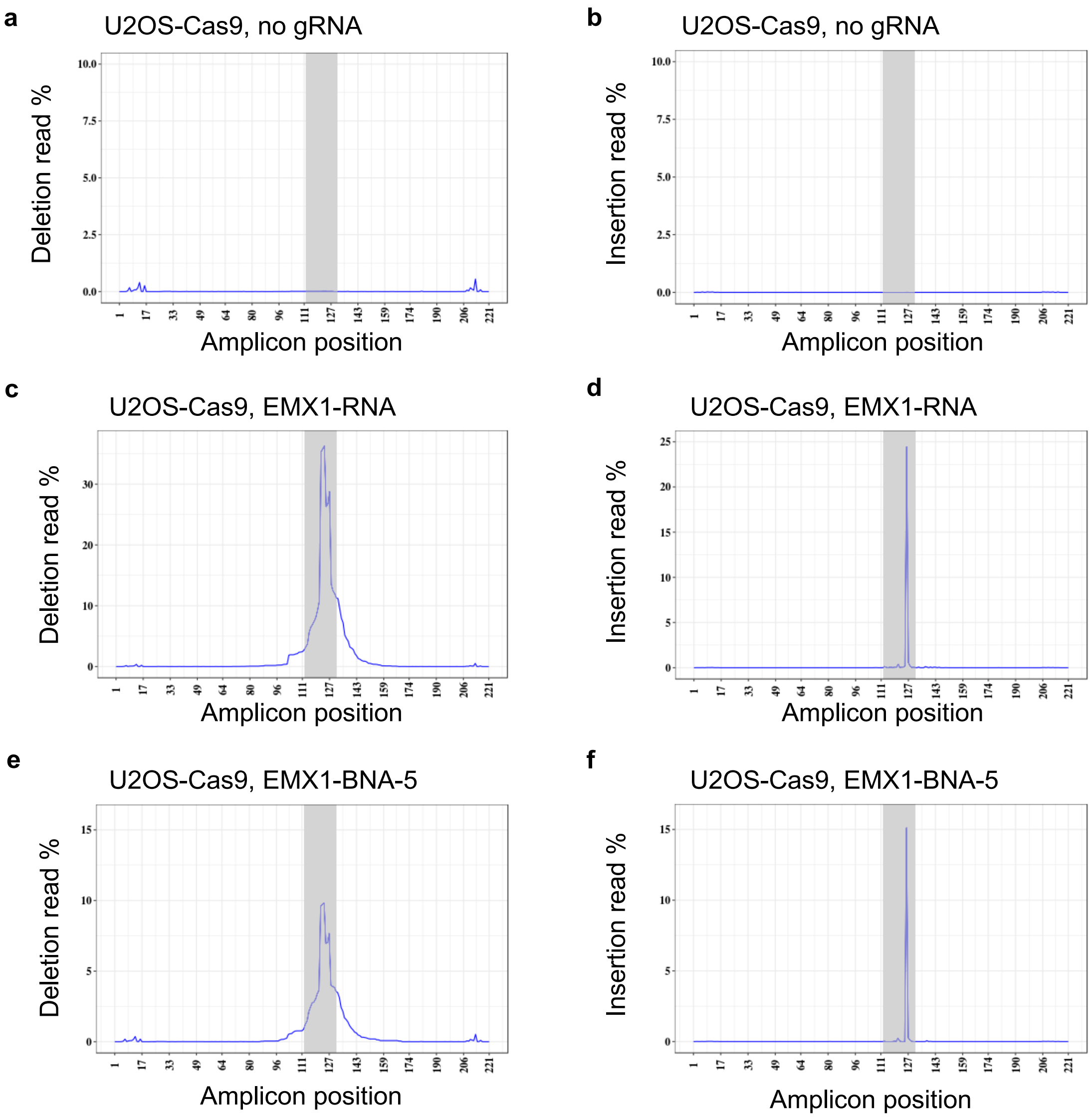

Supplementary Figure 8. Distribution of indel location using unmodified or BNA^{NC}-modified crRNAs on EMX1 target. Distribution frequencies of insertions (right) and deletions (left) across the *EMX1* target site in U2OS-Cas9 cells transfected with no gRNA (**a**, **b**), EMX1-RNA (**c**, **d**) or EMX1-BNA-5 (**e**, **f**) are shown. Grey coloring indicates the location of the genomic CRISPR target site along the amplicon. Distribution of indel positions is based on sequences obtained following high-throughput sequencing of transfected cells.

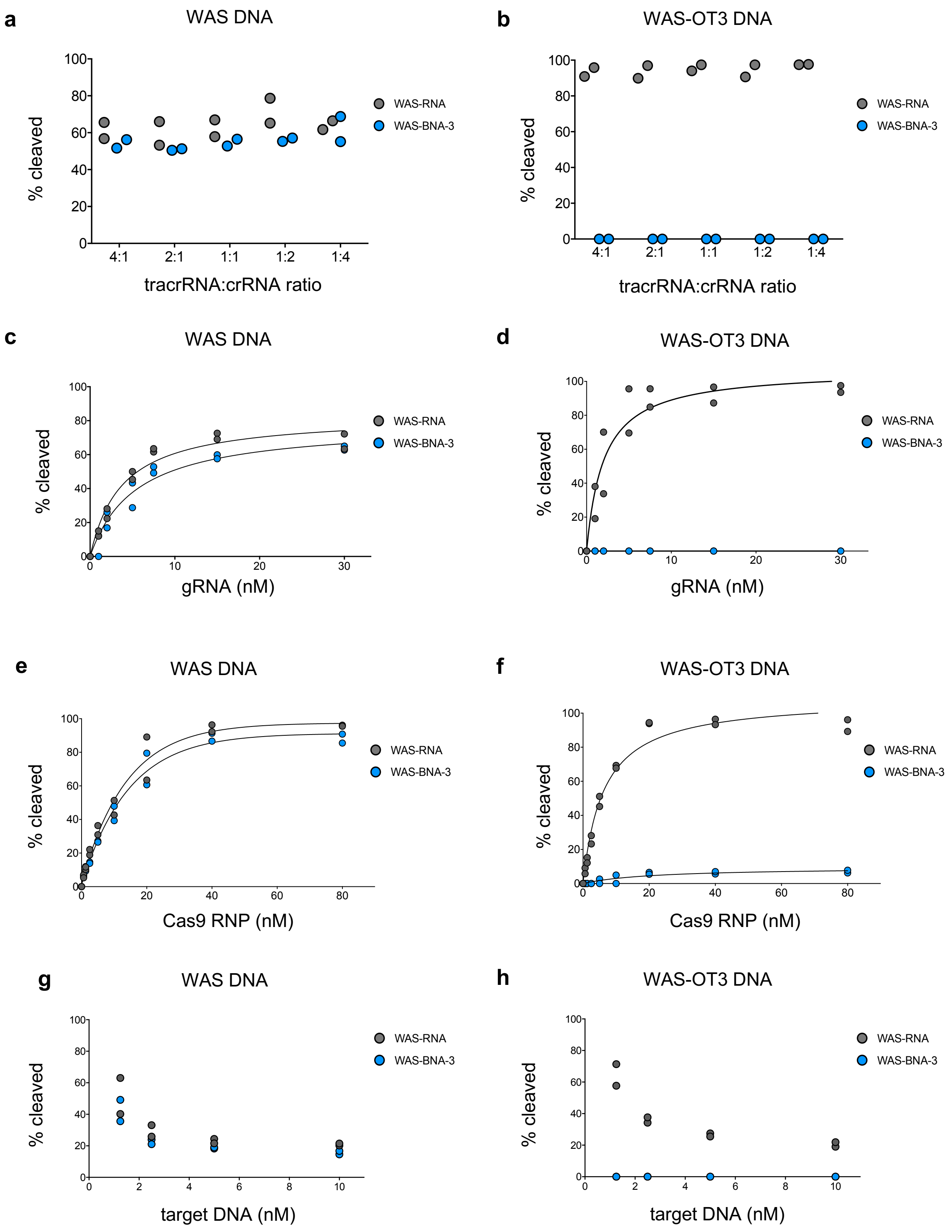
a
WAS DNA
% cleaved
tracrRNA:crRNA ratio
4:1
2:1
1:1
1:2
1:4
WAS-RNA
WAS-BNA-3
b
WAS-OT3 DNA
% cleaved
tracrRNA:crRNA ratio
WAS-RNA
WAS-BNA-3
c
WAS DNA
% cleaved
gRNA (nM)
WAS-RNA
WAS-BNA-3
d
WAS-OT3 DNA
% cleaved
gRNA (nM)
WAS-RNA
WAS-BNA-3
e
WAS DNA
% cleaved
Cas9 RNP (nM)
WAS-RNA
WAS-BNA-3
f
WAS-OT3 DNA
% cleaved
Cas9 RNP (nM)
WAS-RNA
WAS-BNA-3
g
WAS DNA
% cleaved
target DNA (nM)
WAS-RNA
WAS-BNA-3
h
WAS-OT3 DNA
% cleaved
target DNA (nM)
WAS-RNA
WAS-BNA-3

Supplementary Figure 9: Effect of BNA^{NC}-modified crRNAs on affinity, activity, and kinetics of Cas9 *in vitro*. Graph showing the effect of differential tracrRNA/crRNA annealing ratios on (**a**) WAS or (**b**) WAS-OT3 DNA cleavage *in vitro* using WAS-RNA or WAS-BNA-3 crRNA and fixed Cas9 concentrations (15 nM); Individual data points shown (n = 2). gRNAs were annealed to a final concentration of 100 nM using excess tracrRNA or crRNA prior to Cas9 RNP complex formation. Graph showing the effect of increasing gRNA concentrations on (**c**) WAS or (**d**) WAS-OT3 DNA cleavage *in vitro* using WAS-RNA or WAS-BNA-3 crRNA and fixed Cas9 concentrations (15 nM); Individual data points shown (n = 2). Graph showing the effect of increasing Cas9 RNP complex concentration on (**e**) WAS or (**f**) WAS-OT3 DNA cleavage *in vitro* using WAS-RNA or WAS-BNA-3 crRNA; Individual data points shown (n = 2). Graph showing effect of increasing (**g**) WAS or (**h**) WAS-OT3 DNA target on cleavage *in vitro* using WAS-RNA or WAS-BNA-3 crRNA and Cas9 at fixed concentrations; Individual data points shown (n = 2).

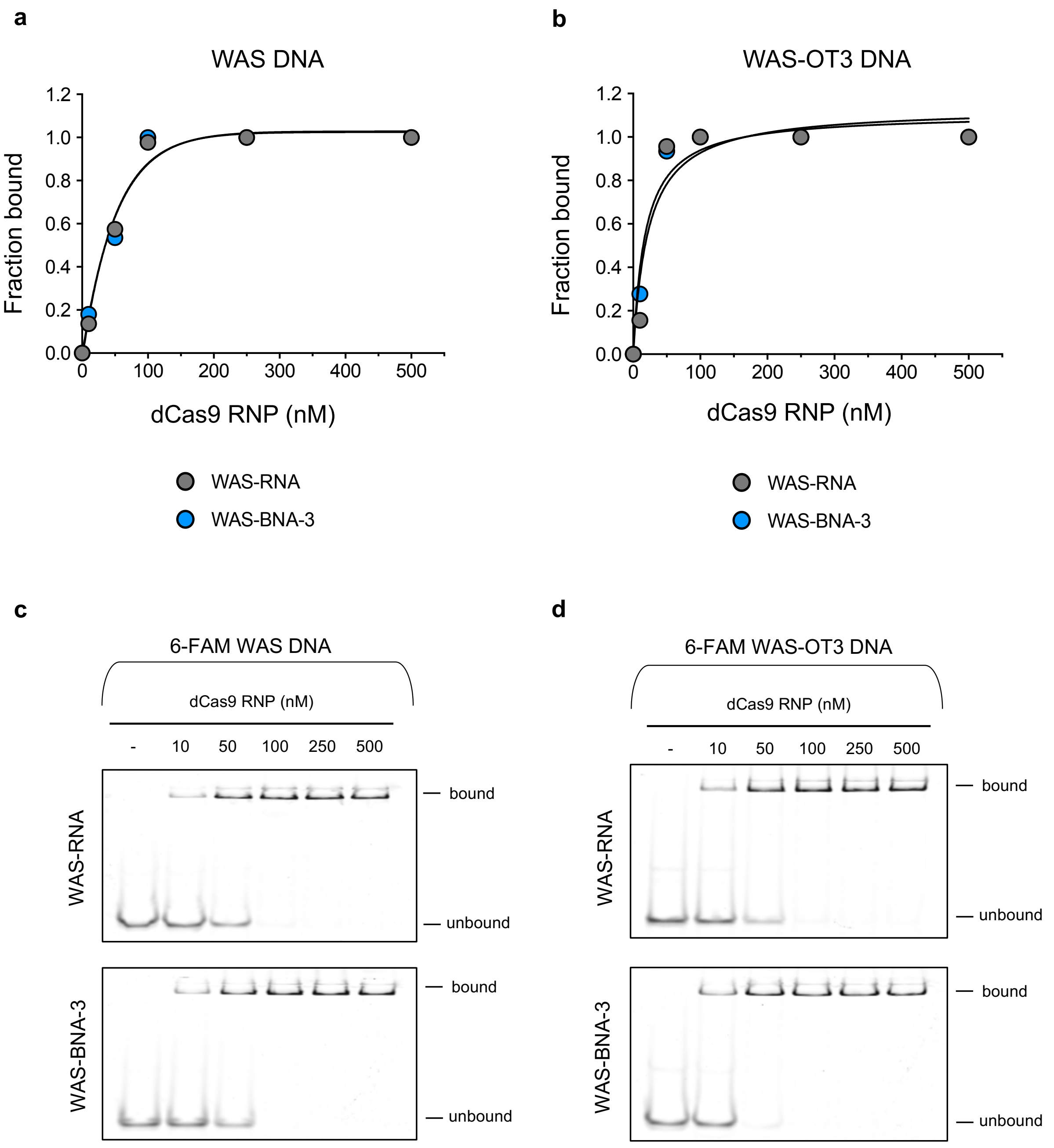

Supplementary Figure 10: Effect of BNA^{NC}-modified crRNAs on the ability of nuclease-deficient Cas9 (dCas9) to bind DNA target sequences. Graph showing the effect of increasing dCas9 RNP concentration on the ability to bind (**a**) WAS or (**b**) WAS-OT3 DNA containing sequences using WAS-RNA (grey) or WAS-BNA-3 (blue) crRNA (n

= 1). Corresponding gel showing the ability of dCas9 to bind (**c**) WAS or (**d**) WAS-OT3 DNA containing sequences using WAS-RNA (top) or WAS-BNA-3 (bottom) crRNAs at increasing concentrations. Substrate DNA concentration remained constant at 50 nM. For all binding experiments, the top band is dCas9-bound DNA, while the bottom band is unbound DNA. Quantification of bound percentages was determined using densitometry (ImageJ).

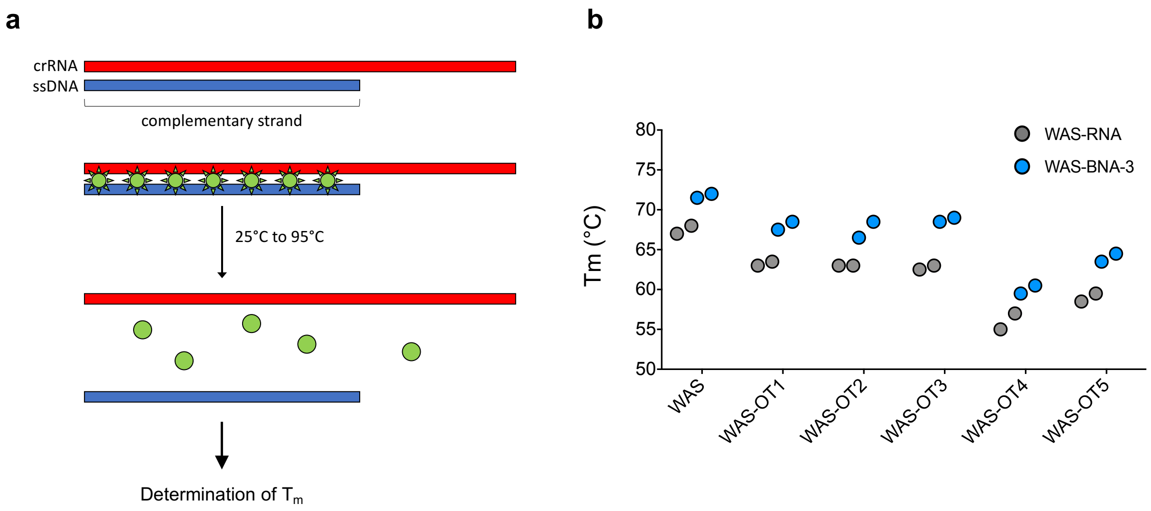

Supplementary Figure 11: Effect of BNA^{NC} modifications on crRNA/DNA melting temperature. (**a**) Schematic diagram of assay used to determine melting temperature. crRNA (red) interacts with complementary ssDNA (blue) to form an RNA / DNA heteroduplex. SYBR Green I (green circle) intercalates and fluoresces during duplex formation. (**b**) Graph showing the experimentally determined melting temperatures for WAS on- and off-targets (listed in **Figure 1b)** using WAS-RNA (grey) or WAS-BNA-3 (blue) crRNA; Individual data points shown (n = 2).

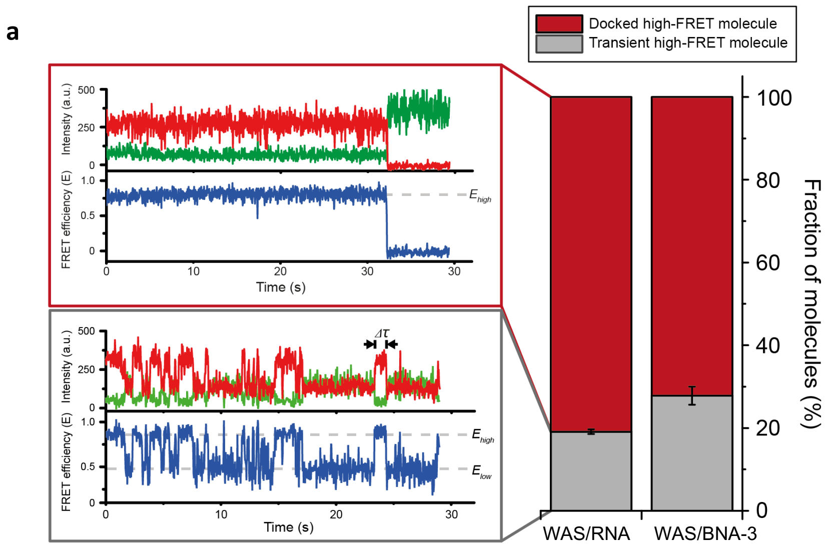

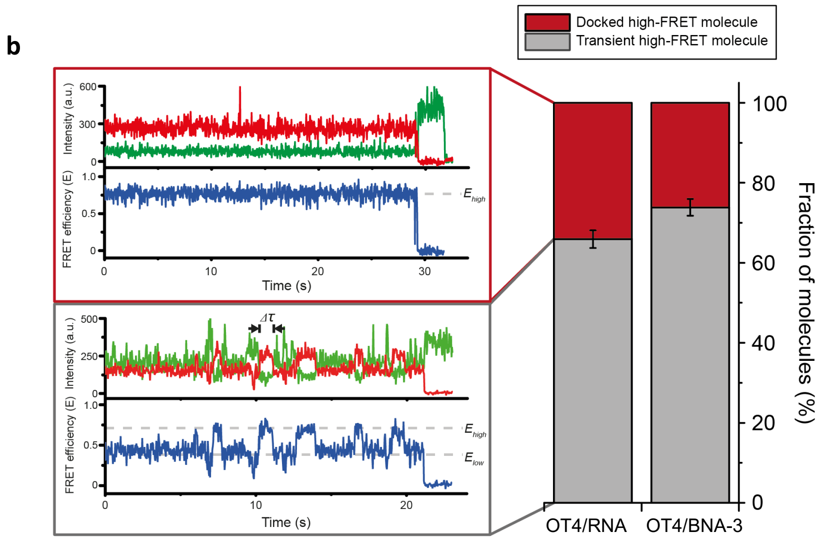

Supplementary Figure 12: Kinetic analysis of time spent in open to zipped transition and docked states using single-molecule FRET. Fraction of molecules observed in either docked high-, or transient high-FRET states for unmodified and BNA^{NC}-modified crRNAs using (**a**) WAS or (**b**) WAS-OT4 DNA containing sequences. Representative time-trajectories reveal two distinct modes within the high-FRET state; docked mode (red) and transient mode (grey).

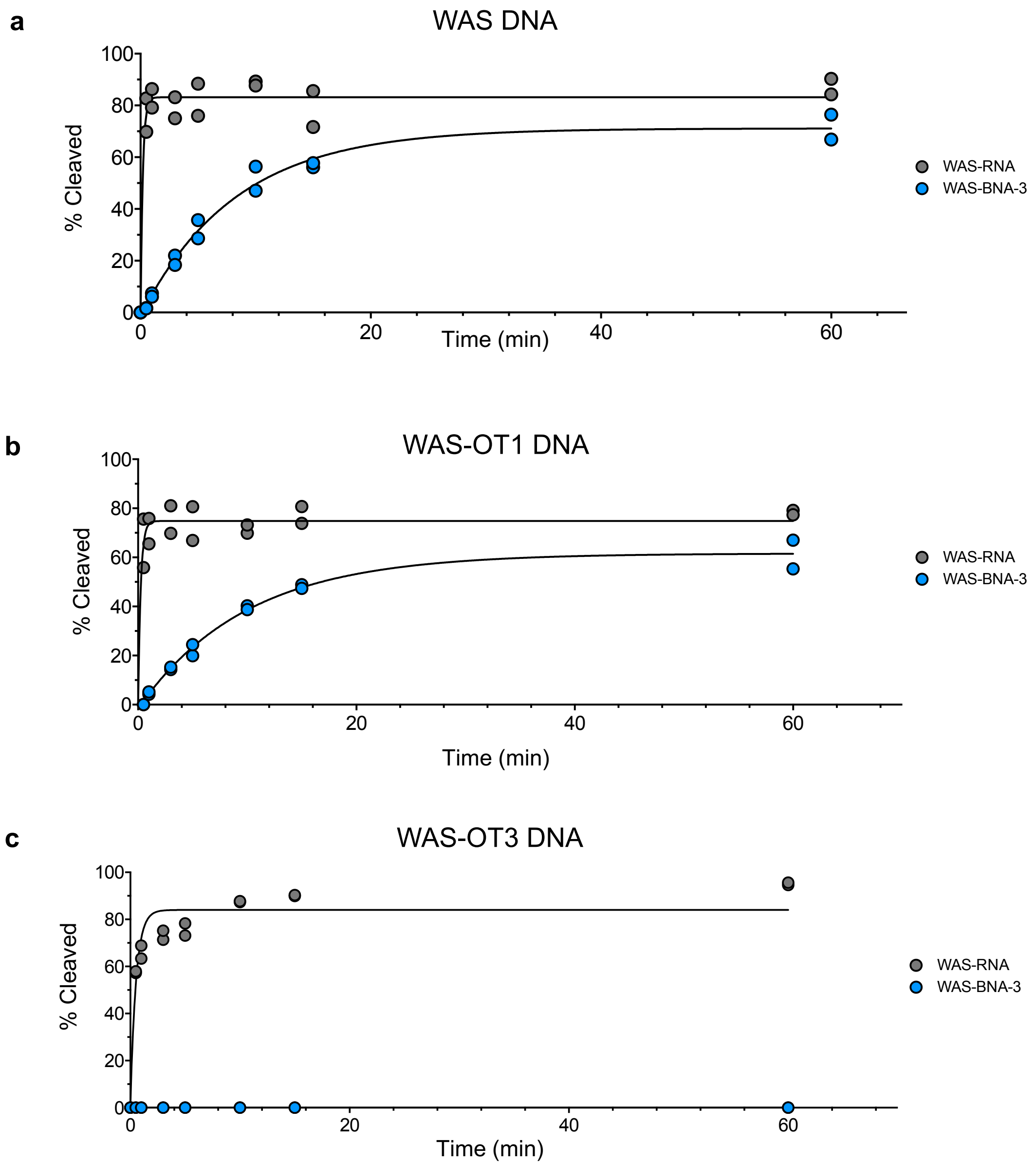

Supplementary Figure 13: Cas9 *in vitro* cleavage kinetics using either WAS-RNA or WAS-BNA-3 crRNAs on several target sequences. Graph showing the *in vitro* cleavage of (**a**) WAS, (**b**) WAS-OT1 or (**c**) WAS-OT3 DNA containing sequences using either WAS-RNA (grey) or WAS-BNA-3 (blue) crRNA over time; Individual data points shown (n = 2). The molar ratio of Cas9 RNP complex to target DNA was 3:1 for these experiments.

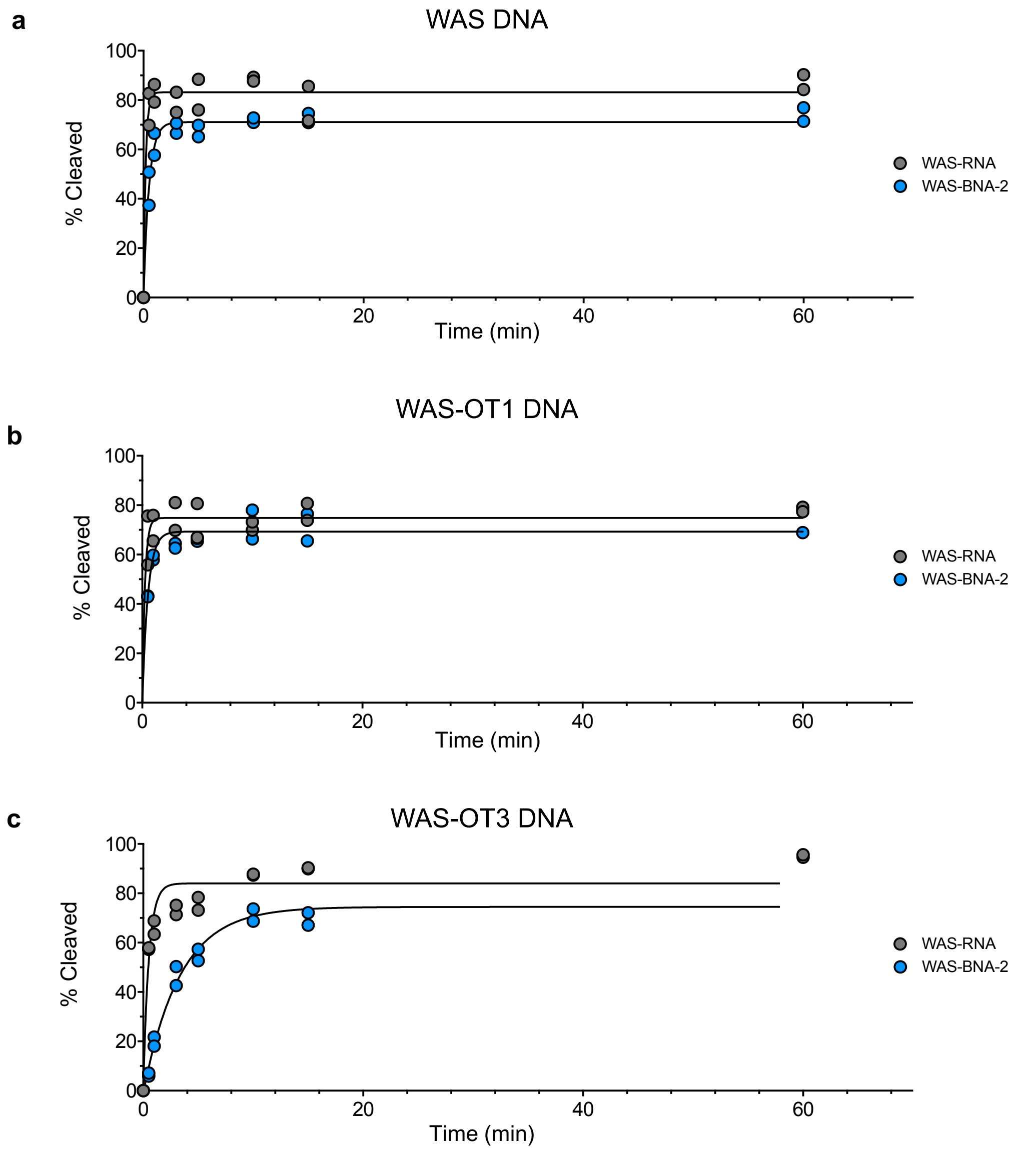

Supplementary Figure 14: Cas9 *in vitro* cleavage kinetics using either WAS-RNA or WAS-BNA-2 crRNAs on several target sequences. Graph showing the *in vitro* cleavage of (**a**) WAS, (**b**) WAS-OT1 or (**c**) WAS-OT3 DNA containing sequences using either WAS-RNA (grey) or WAS-BNA-2 (blue) crRNA over time; Individual data points shown (n = 2). The molar ratio of Cas9 RNP complex to target DNA was 3:1 for these experiments. WAS-RNA values from **Supplementary Fig. 13** are shown again as reference.

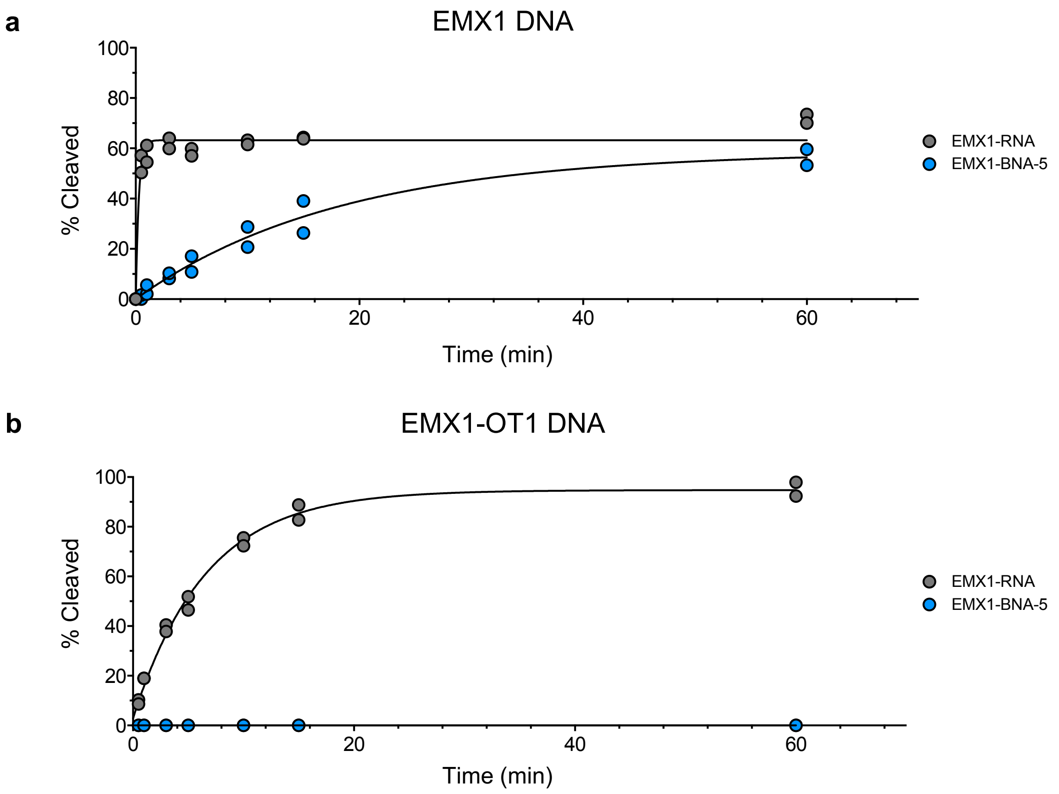

Supplementary Figure 15: Cas9 *in vitro* cleavage kinetics using either EMX1-RNA or EMX1-BNA-5 crRNAs on several target sequences. Graph showing the *in vitro* cleavage of (**a**) EMX1 or (**b**) EMX1-OT1 DNA containing sequences using either EMX1-RNA (grey) or EMX1-BNA-5 (blue) crRNA over time; Individual data points shown (n = 2). The molar ratio of Cas9 RNP complex to target DNA was 3:1 for these experiments.

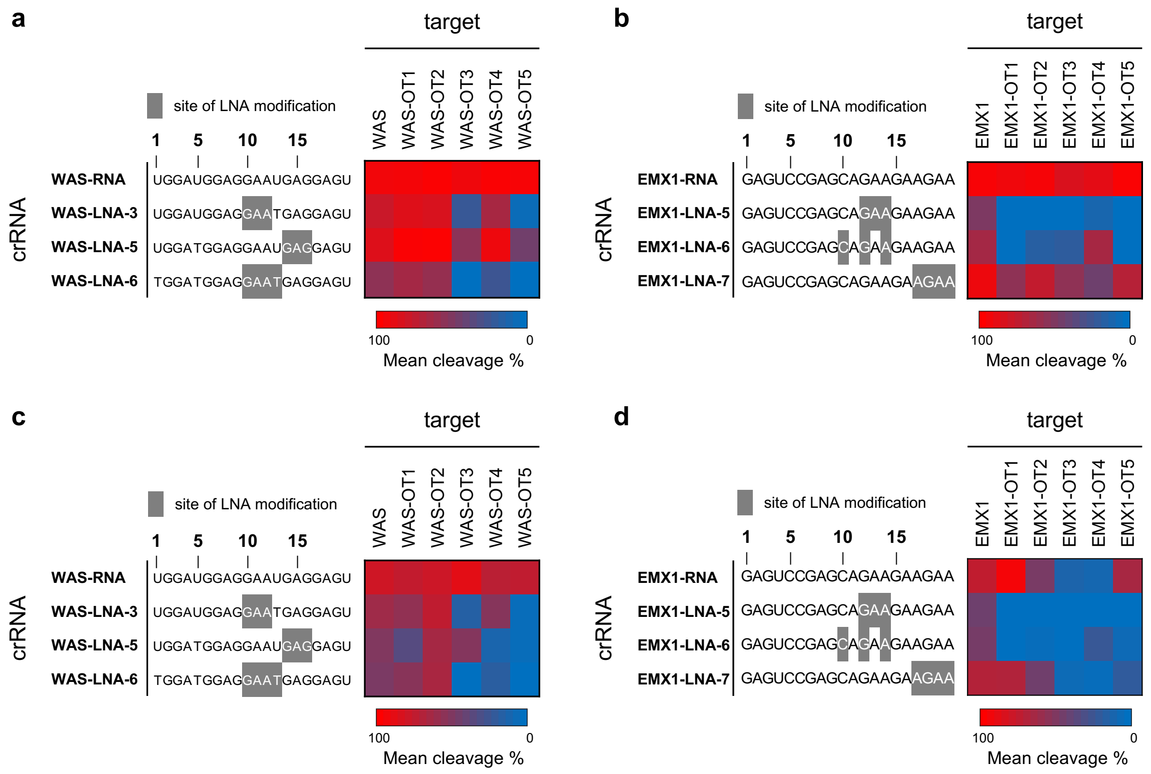

Supplementary Figure 16: Incorporation of LNA into crRNAs improves Cas9 cleavage specificity *in vitro.* Heat map showing cleavage specificity for unmodified and 3 LNA-modified crRNAs towards either (**a**) WAS or (**b**) EMX1 on- and off-target sequences (as listed in **Fig. 1b**, **c**); Mean shown (n = 2). LNA modifications are indicated in grey. Targets which were highly cleaved *in vitro* are indicated by red, while targets which were not cleaved are indicated by blue. The molar ratio of Cas9 RNP complex to target DNA was 30:1 for these experiments. Values used to generate heatmaps are shown in **Supplementary Table 6**. Heatmap showing cleavage specificity for unmodified and 3 LNA-modified crRNAs towards either (**c**) WAS or (**d**) EMX1 on- and off-target sequences; Mean shown (n = 2). The molar ratio of Cas9 RNP complex to target DNA was 3:1 for these experiments. Values used to generate

heatmaps are shown in **Supplementary Table 6**. WAS-RNA and EMX1-RNA values from **Fig. 1** and **Supplementary Fig. 1** are shown again as reference.

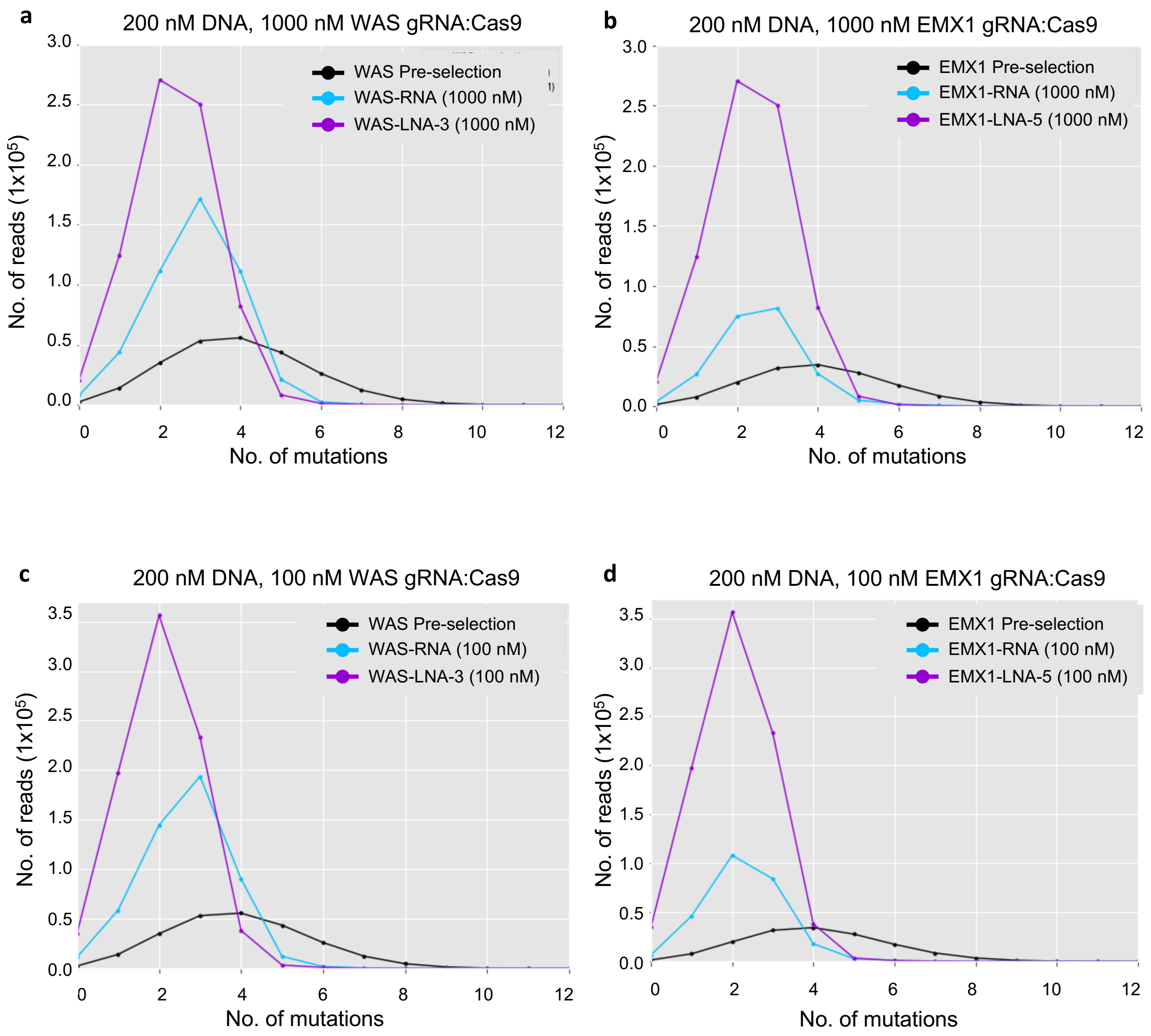

Supplementary Figure 17: Distribution of mutations in pre- and post-selection libraries following *in vitro* library selection with unmodified or LNA-modified crRNAs. *In vitro* selections were performed using 200 nM pre-selection library with 1000 nM Cas9 RNP complex targeting either (**a**) WAS or (**b**) EMX1. *In vitro* selections were performed using 200 nM pre-selection library with 100 nM Cas9 RNP complex targeting either (**c**) WAS or (**d**) EMX1. Cas9 RNP complexes were pre-assembled using unmodified or LNA-modified crRNAs prior to DNA digestion. Distributions of mutations within pre-selection (black) and post-selection (colored) libraries are shown. Mutations are counted

from the 20-base-pairs comprising the Cas9 target site, as well as the 2-base-pair PAM (N of NGG is excluded). Pre-selection, WAS-RNA and EMX1-RNA values from **Supplementary Fig. 3** are shown again as reference.

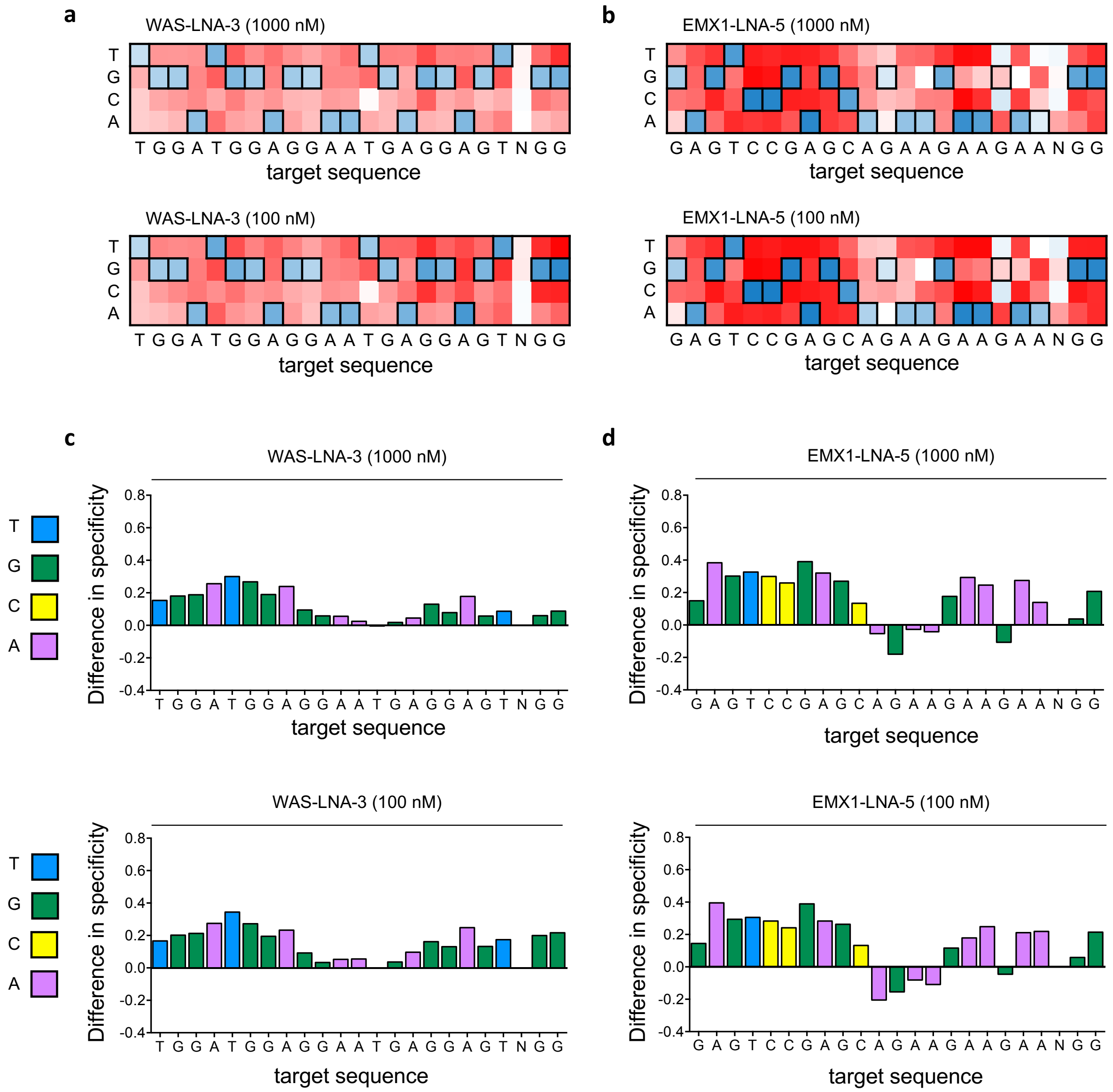

Supplementary Figure 18: *In vitro* specificity profiling results for LNA-modified crRNAs. Heat maps showing DNA cleavage specificity scores across >10^{12} off-target sequences for LNA-modified crRNAs targeting (**a**) WAS or (**b**) EMX1 using 1000 nM Cas9 RNP complex (top) or 100 nM Cas9 RNP complex (bottom). Specificity scores of 1.0 (dark blue) correspond to 100% enrichment for, while scores of -1.0 (dark red) correspond to 100% enrichment against a specific base-pair at a specific position. Black boxes denote the intended target nucleotides. Bar graph showing the quantitative difference in

specificity score at each position in the 20 base-pair target site and 2 base-pair PAM (N of NGG excluded), between the unmodified (shown in **Fig. 2** and **Supplementary Fig. 4**) and LNA-modified crRNA for (**c**) WAS or (**d**) EMX1 target sequences using 1000 and 100 nM Cas9 RNP complex. A score of zero indicates no change in specificity. Difference in specificity was calculated as, specificity $score_{BNA}^{NC}$ – specificity $score_{RNA}$.

a

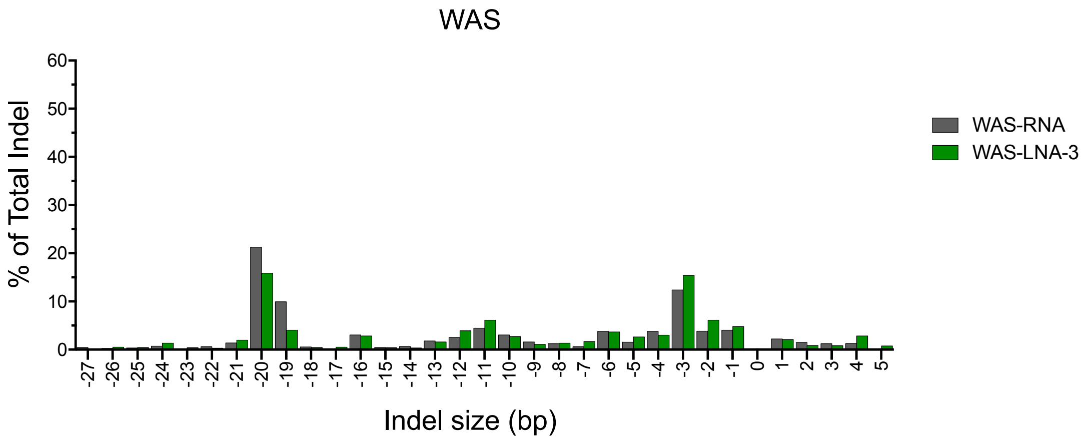

b

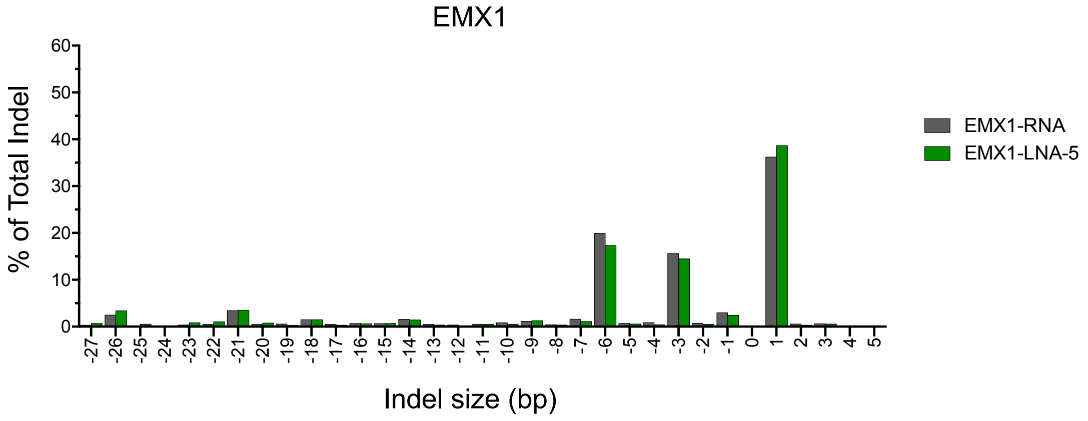

Supplementary Figure 19. Comparison of indel size resulting from Cas9 DNA cleavage with unmodified or LNA-modified crRNAs. Distributions of indel sizes are shown for either (**a**) WAS or (**b**) EMX1 using unmodified or LNA-modified crRNAs in U2OS-Cas9 cells. Distribution of indel sizes is based on sequences obtained following high-throughput sequencing of transfected cells. The number of reads for each indel size was normalized to the total number of indel reads. Only indels ranging from -27 bp (27 bp deletion) to +5 bp (5 bp insertion) are shown. WAS-RNA and EMX1-RNA values from **Supplementary Fig. 6** are shown again as reference.

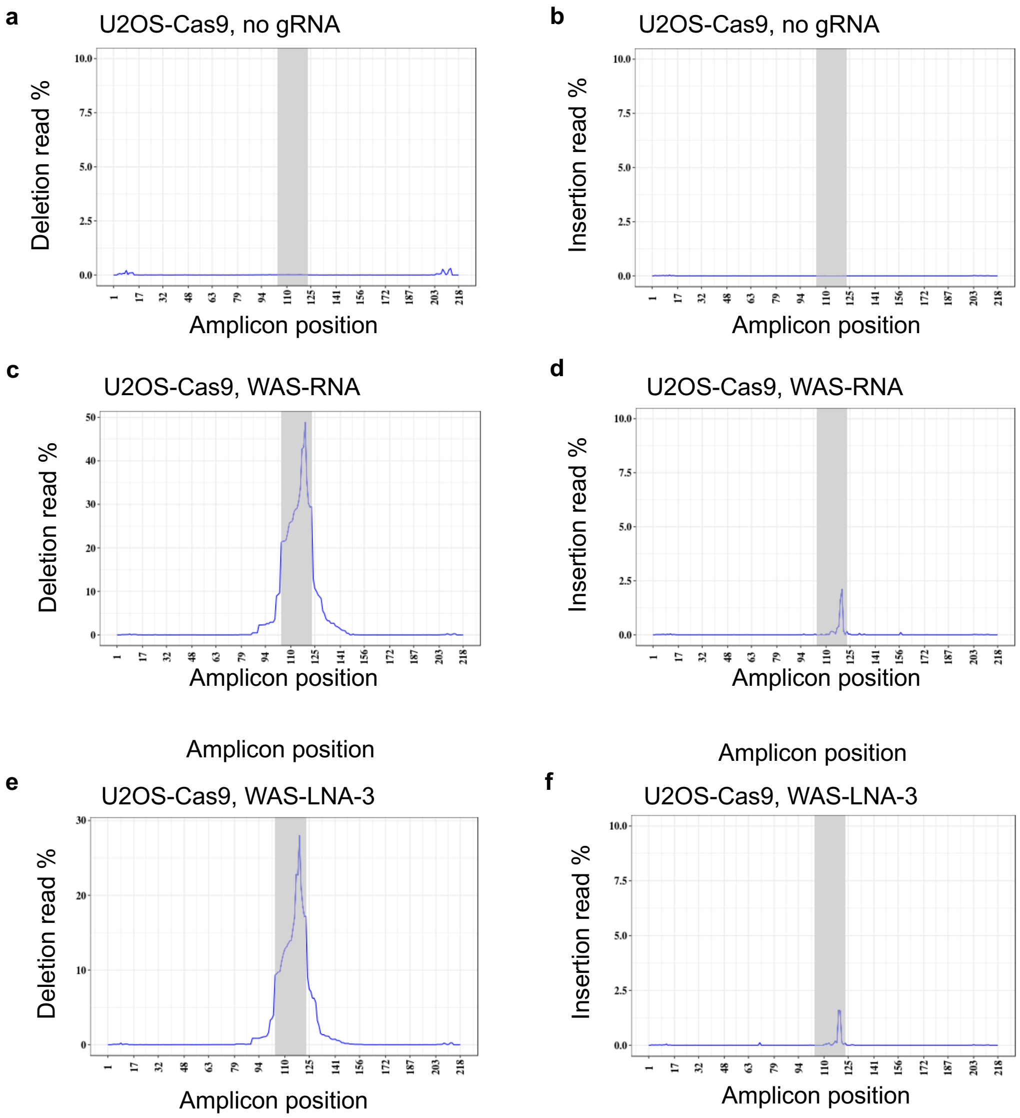

Supplementary Figure 20. Distribution of indel location using unmodified or LNA-modified crRNAs on WAS target. Distribution frequencies of insertions (right) and deletions (left) across the *WAS* target site in U2OS-Cas9 cells transfected with no gRNA (**a**, **b**), WAS-RNA (**c**, **d**) or WAS-LNA-3 (**e**, **f**) are shown. Grey coloring indicates the location of the genomic CRISPR target site along the amplicon. Distribution of indel positions is based on sequences obtained following high-throughput sequencing of transfected cells. No gRNA and WAS-RNA values from **Supplementary Fig. 7** are shown again as reference.

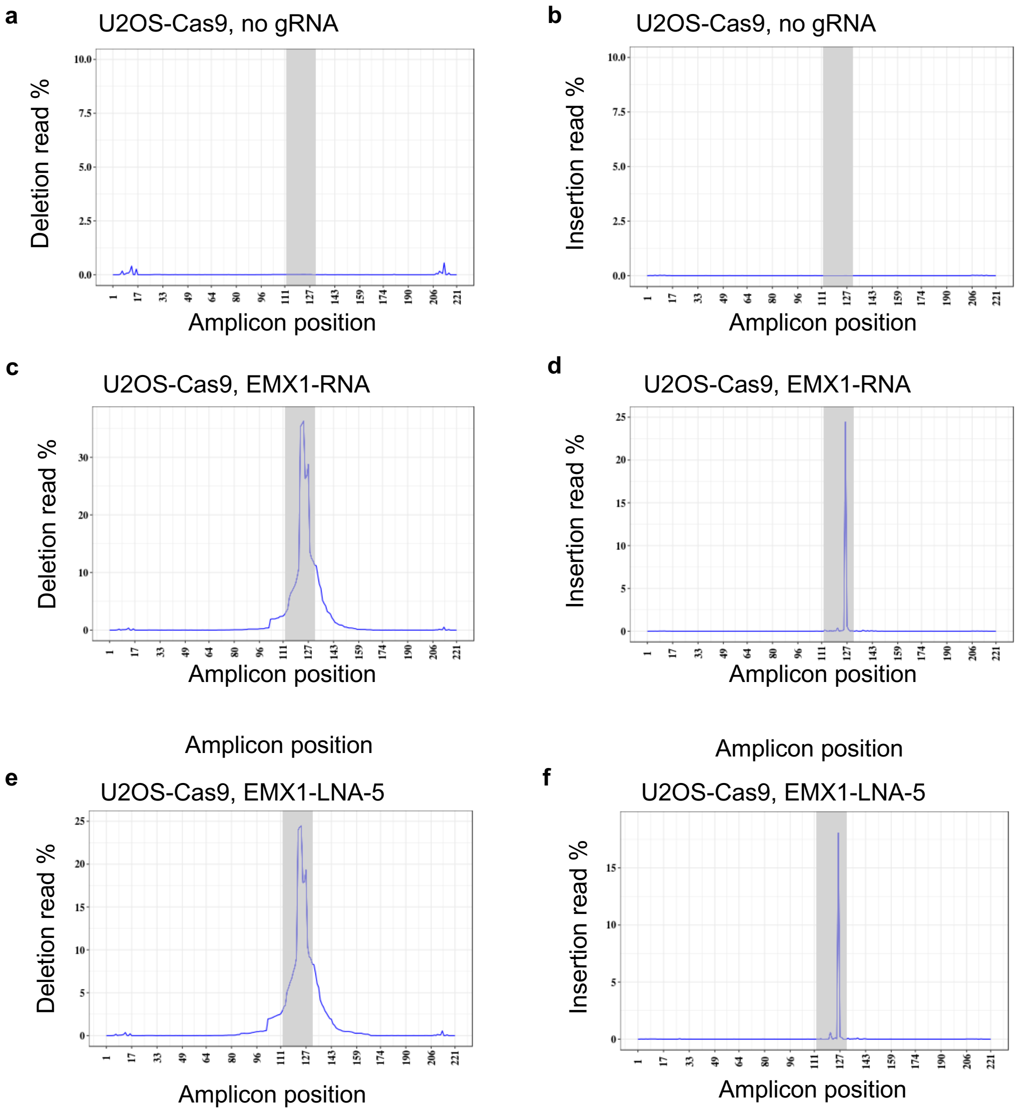

Supplementary Figure 21. Distribution of indel location using unmodified or LNA-modified crRNAs on EMX1 target. Distribution frequencies of insertions (right) and deletions (left) across the *EMX1* target site in U2OS-Cas9 cells transfected with no gRNA (**a**, **b**), EMX1-RNA (**c**, **d**) or EMX1-LNA-5 (**e**, **f**) are shown. Grey coloring indicates the location of the genomic CRISPR target site along the amplicon. Distribution of indel positions is based on sequences obtained following high-throughput sequencing of transfected cells. No gRNA and EMX1-RNA values from **Supplementary Fig. 8** are shown again as reference.

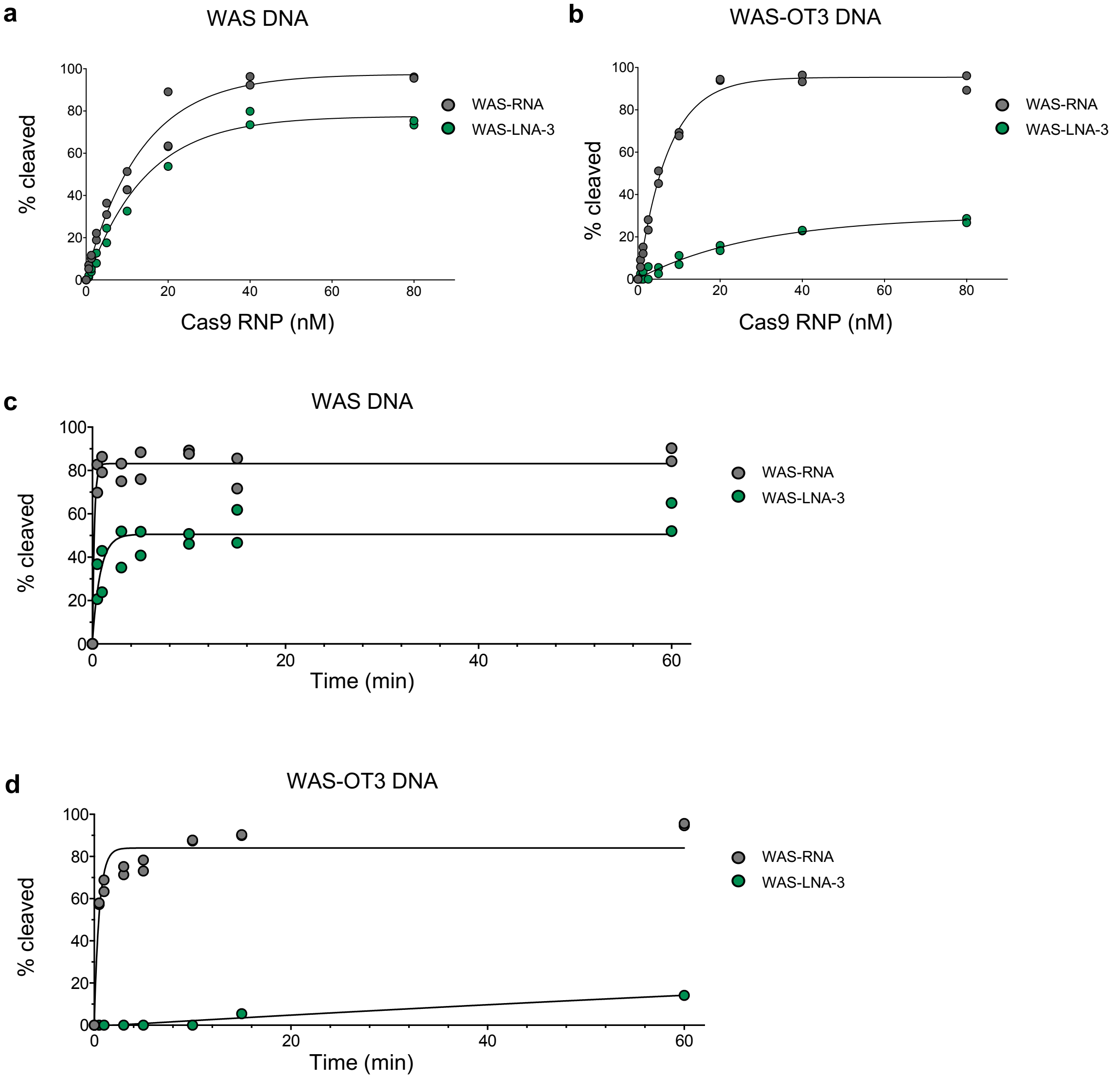

Supplementary Figure 22: Effect of LNA-modified crRNAs on activity and kinetics of Cas9 *in vitro*. Graph showing the effect of increasing Cas9 RNP complex concentration on WAS (**a**) or WAS-OT3 (**b**) DNA cleavage using WAS-RNA or WAS-LNA-3 crRNA; Individual data points are shown (n = 2). Graph showing the *in vitro* cleavage of (**c**) WAS or (**d**) WAS-OT3 DNA containing sequences using either WAS-RNA or WAS-LNA-3 crRNAs over time; Individual data points are shown (n =2). The molar ratio of Cas9 RNP complex to target DNA was 3:1 for **c** and **d**. WAS-RNA values from **Supplementary Figs. 9** and **13** are shown again as reference.

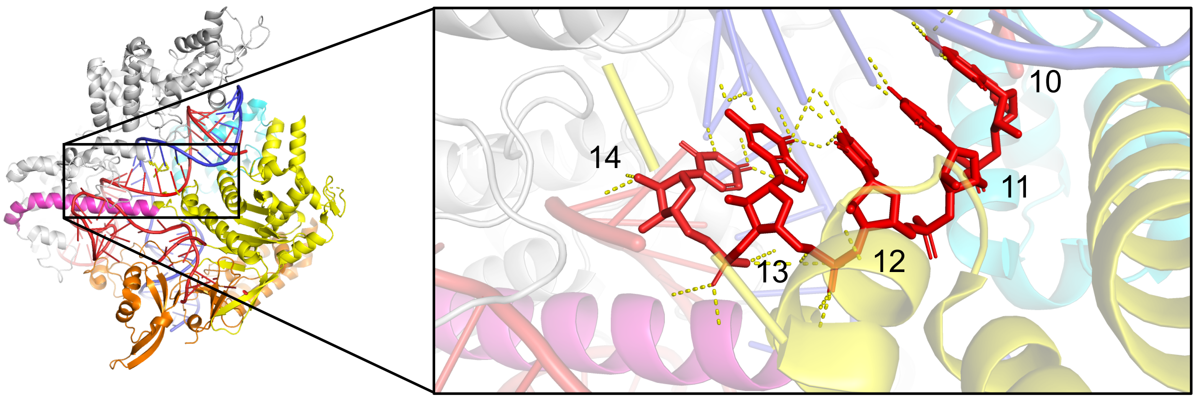

Supplementary Figure 23. Model of Cas9 structure highlighting interactions between crRNA nucleotides 10-14 and Cas9. Pymol model of Cas9 in complex with gRNA (red) and target DNA (blue) (PDB: 4UN3)[1] with domains colored (RuvC – yellow, Rec I and Rec II – grey, bridge helix – magenta, HNH – cyan, and PAM interacting – orange) (left). Contacts between nucleotides 10-14 of the crRNA and Cas9 and highlighted in the magnified image (right). Yellow dashed lines indicate predicted hydrogen bonds.

Supplementary Table 1. *In vitro* cleavage assay data used to generate Fig. 1.

crRNA	Target					
	**WAS**	**WAS-OT1**	**WAS-OT2**	**WAS-OT3**	**WAS-OT4**	**WAS-OT5**
**WAS-RNA**	95 ± 1.6	96 ± 2.3	99 ± 0.10	94 ± 0.10	98 ± 0.0	97 ± 0.13
**WAS-BNA-1**	93 ± 0.04	97 ± 0.28	97 ± 0.11	72 ± 2.2	88 ± 0.82	26 ± 0.50
**WAS-BNA-2**	94 ± 0.89	97 ± 0.06	96 ± 0.29	83 ± 1.2	91 ± 0.21	71 ± 1.9
**WAS-BNA-3**	67 ± 7.1	66 ± 6.3	9.3 ± 1.7	1.8 ± 1.8	1.9 ± 1.9	0.0 ± 0.0
**WAS-BNA-4**	88 ± 1.7	92 ± 1.5	92 ± 1.2	83 ± 0.42	85 ± 0.56	84 ± 0.80
**WAS-BNA-5**	99 ± 0.24	91 ± 2.7	98 ± 0.21	77 ± 0.62	19 ± 1.4	14 ± 0.66
**WAS-BNA-6**	71 ± 2.2	66 ± 6.2	4.1 ± 4.1	0.0 ± 0.0	0.0 ± 0.0	0.0 ± 0.0
**WAS-BNA-7**	89 ± 0.28	91 ± 1.3	61 ± 4.0	4.2 ± 4.2	42 ± 5.8	0.0 ± 0.0
**WAS-BNA-8**	93 ± 0.21	41 ± 1.4	76 ± 1.4	90 ± 0.88	7.7 ± 1.4	19 ± 5.3
**WAS-BNA-9**	46 ± 10	4.2 ± 4.2	21 ± 0.58	53 ± 11	0.0 ± 0.0	4.9 ± 4.9

crRNA	Target					
	**EMX1**	**EMX1-OT1**	**EMX1-OT2**	**EMX1-OT3**	**EMX1-OT4**	**EMX1-OT5**
**EMX1-RNA**	95 ± 2.5	83 ± 11	92 ± 4.4	66 ± 19	67 ± 23	95 ± 3.2
**EMX1-BNA-1**	97 ± 0.30	82 ± 2.5	89 ± 0.77	95 ± 1.7	91 ± 2.9	91 ± 2.2
**EMX1-BNA-2**	52 ± 2.4	0.0 ± 0.0	0.0 ± 0.0	0.0 ± 0.0	0.0 ± 0.0	0.0 ± 0.0
**EMX1-BNA-3**	94 ± 0.50	92 ± 0.45	91 ± 0.20	37 ± 0.61	90 ± 0.0	97 ± 0.05
**EMX1-BNA-4**	11 ± 2.6	0.0 ± 0.0	0.0 ± 0.0	0.0 ± 0.0	0.0 ± 0.0	0.0 ± 0.0
**EMX1-BNA-5**	75 ± 1.1	0.0 ± 0.0	0.0 ± 0.0	0.0 ± 0.0	0.0 ± 0.0	0.0 ± 0.0
**EMX1-BNA-6**	52 ± 2.0	0.0 ± 0.0	0.0 ± 0.0	0.0 ± 0.0	2.2 ± 2.2	0.0 ± 0.0
**EMX1-BNA-7**	97 ± 0.05	52 ± 2.6	42 ± 4.0	0.0 ± 0.0	0.0 ± 0.0	0.0 ± 0.0
**EMX1-BNA-8**	85 ± 0.04	17 ± 5.5	40 ± 1.0	0.0 ± 0.0	16 ± 6.3	45 ± 0.24
**EMX1-BNA-9**	0.0 ± 0.0	0.0 ± 0.0	0.0 ± 0.0	0.0 ± 0.0	0.0 ± 0.0	0.0 ± 0.0

Values showing *in vitro* cleavage specificity for unmodified crRNA and 9 BNA^{NC}-modified crRNAs towards either WAS or EMX1 on- and off-target sequences (as listed **Fig. 1b**, **c**); Mean ± SE (n = 2). Experiments were performed using 150 nM Cas9 RNP and 5 nM DNA.

Supplementary Table 2. ***In vitro*** **cleavage assay data used to generate Supplementary Fig. 1.**

crRNA	Target<br>WAS	WAS-OT1	WAS-OT2	WAS-OT3	WAS-OT4	WAS-OT5
**WAS-RNA**	81 ± 16	76 ± 2.8	80 ± 1.5	89 ± 0.72	73 ± 4.0	75 ± 8.2
**WAS-BNA-1**	69 ± 2.0	72 ± 2.4	95 ± 1.9	54 ± 3.1	66 ± 1.1	10 ± 1.2
**WAS-BNA-2**	67 ± 0.82	70 ± 0.17	77 ± 7.5	59 ± 0.70	58 ± 2.0	39 ± 1.7
**WAS-BNA-3**	63 ± 8.8	67 ± 10	9.3 ± 1.2	5.6 ± 0.08	7.5 ± 2.9	0.0 ± 0.0
**WAS-BNA-4**	68 ± 13	64 ± 4.0	85 ± 4.6	91 ± 0.15	59 ± 3.1	66 ± 0.48
**WAS-BNA-5**	65 ± 7.3	16 ± 4.1	34 ± 5.0	23 ± 4.1	0.0 ± 0.0	0.0 ± 0.0
**WAS-BNA-6**	55 ± 8.2	53 ± 8.9	2.9 ± 2.9	0.0 ± 0.0	0.0 ± 0.0	0.0 ± 0.0
**WAS-BNA-7**	66 ± 6.6	64 ± 1.4	43 ± 2.5	2.2 ± 2.2	26 ± 6.4	0.0 ± 0.0
**WAS-BNA-8**	47 ± 3.0	31 ± 1.9	25 ± 1.2	66 ± 0.05	9.7 ± 1.2	23 ± 1.3
**WAS-BNA-9**	25 ± 0.90	10 ± 4.4	22 ± 0.34	39 ± 3.0	5.7 ± 5.7	4.4 ± 4.4

crRNA	Target<br>EMX1	EMX1-OT1	EMX1-OT2	EMX1-OT3	EMX1-OT4	EMX1-OT5
**EMX1-RNA**	65 ± 1.8	92 ± 3.0	46 ± 2.6	9.6 ± 0.71	8.7 ± 0.13	48 ± 17
**EMX1-BNA-1**	63 ± 6.2	73 ± 9.6	50 ± 4.2	17 ± 4.1	16 ± 4.3	44 ± 13
**EMX1-BNA-2**	4.9 ± 2.4	0.0 ± 0.0	0.0 ± 0.0	0.0 ± 0.0	0.0 ± 0.0	0.0 ± 0.0
**EMX1-BNA-3**	63 ± 1.8	61 ± 0.65	47 ± 2.2	4.1 ± 4.1	18 ± 0.20	59 ± 1.2
**EMX1-BNA-4**	7.4 ± 1.2	0.0 ± 0.0	0.0 ± 0.0	0.0 ± 0.0	0.0 ± 0.0	0.0 ± 0.0
**EMX1-BNA-5**	55 ± 4.0	0.0 ± 0.0	0.0 ± 0.0	0.0 ± 0.0	0.0 ± 0.0	0.0 ± 0.0
**EMX1-BNA-6**	38 ± 1.1	0.0 ± 0.0	0.0 ± 0.0	0.0 ± 0.0	0.0 ± 0.0	0.0 ± 0.0
**EMX1-BNA-7**	69 ± 12	8.6 ± 0.32	3.2 ± 3.2	0.0 ± 0.0	0.0 ± 0.0	0.0 ± 0.0
**EMX1-BNA-8**	50 ± 2.1	6.0 ± 1.4	0.0 ± 0.0	0.0 ± 0.0	0.0 ± 0.0	0.0 ± 0.0
**EMX1-BNA-9**	0.0 ± 0.0	0.0 ± 0.0	0.0 ± 0.0	0.0 ± 0.0	0.0 ± 0.0	0.0 ± 0.0

Values showing *in vitro* cleavage specificity for unmodified crRNA and 9 BNA^{NC}-modified crRNAs towards either WAS or EMX1 on- and off-target sequences (as listed **Fig. 1b**, **c**); Mean ± SE (n = 2). Experiments were performed using 15 nM Cas9 RNP and 5 nM DNA.

Supplementary Table 3. ***In vitro*** **cleavage assay data used to generate Supplementary Fig. 2.**

	crRNA	
**Target**	**WAS-RNA**	**WAS-BNA-3**
**WAS**	86 ± 7.5	66 ± 9.3
**WAS-m1**	54 ± 2.7	32 ± 3.5
**WAS-m2**	61 ± 8.6	12 ± 2.1
**WAS-m3**	30 ± 1.0	0.0 ± 0.0
**WAS-m4**	66 ± 1.5	62 ± 4.7
**WAS-m5**	65 ± 5.8	57 ± 3.4
**WAS-m6**	79 ± 2.8	57 ± 1.1
**WAS-m7**	70 ± 5.3	3.2 ± 3.2
**WAS-m8**	85 ± 1.9	4.0 ± 0.70
**WAS-m9**	76 ± 3.1	50 ± 0.50
**WAS-m10**	74 ± 2.2	9.7 ± 1.5
**WAS-m11**	65 ± 0.22	11 ± 0.07

	crRNA	
**Target**	**EMX1-RNA**	**EMX1-BNA-5**
**EMX1**	71 ± 9.1	56 ± 7.3
**EMX1-m1**	52 ± 9.8	29 ± 1.2
**EMX1-m2**	57 ± 5.3	11 ± 0.26
**EMX1-m3**	53 ± 8.3	5.4 ± 1.1
**EMX1-m4**	55 ± 7.5	51 ± 0.23
**EMX1-m5**	68 ± 4.3	56 ± 1.5
**EMX1-m6**	75 ± 2.0	22 ± 3.3
**EMX1-m7**	72 ± 8.7	20 ± 0.93
**EMX1-m8**	84 ± 4.0	11 ± 0.14
**EMX1-m9**	72 ± 1.1	78 ± 6.3
**EMX1-m10**	70 ± 0.52	9.9 ± 1.4
**EMX1-m11**	69 ± 0.06	14 ± 0.31

Values showing *in vitro* cleavage specificity for unmodified crRNA and 9 BNA^{NC}-modified crRNAs towards either WAS (left) or EMX1 (right) on- and off-target sequences containing single-nucleotide mismatches (as listed in **Supplementary Figure 2)**; Mean ± SD (n = 2). Experiments were performed using 15 nM Cas9 RNP and 5 nM DNA.

Supplementary Table 4. Statistics of sequences from *in vitro* high-throughput library selection experiment.

Selection	Sequence count	Mean mutations	SD mutations	*P*-value
WAS-RNA (1000 nM)	470856	2.880	1.119	$< 2.2 \times 10^{-16}$
WAS-BNA-3 (1000 nM)	635675	1.999	0.948	$< 2.2 \times 10^{-16}$
WAS-LNA-3 (1000 nM)	758713	2.372	1.026	$< 2.2 \times 10^{-16}$
WAS-RNA (100 nM)	513723	2.660	1.076	$< 2.2 \times 10^{-16}$
WAS-BNA-3 (100 nM)	783753	1.747	0.924	$< 2.2 \times 10^{-16}$
WAS-LNA-3 (100 nM)	865363	2.070	0.949	$< 2.2 \times 10^{-16}$
WAS pre-selection	249602	3.945	1.735	NA
EMX1-RNA (1000 nM)	222094	2.585	1.097	$< 2.2 \times 10^{-16}$
EMX1-BNA-5 (1000 nM)	295408	1.646	0.928	$< 2.2 \times 10^{-16}$
EMX1-LNA-5 (1000 nM)	338344	1.893	0.922	$< 2.2 \times 10^{-16}$
EMX1-RNA (100 nM)	268000	2.289	1.003	$< 2.2 \times 10^{-16}$
EMX1-BNA-5 (100 nM)	304230	1.342	0.956	$< 2.2 \times 10^{-16}$
EMX1-LNA-5 (100 nM)	390684	1.652	0.873	$< 2.2 \times 10^{-16}$
EMX1 pre-selection	154510	4.063	1.763	NA

Statistics are shown for pre-selection libraries, as well as post-selection libraries following digestion with unmodified, BNA^{NC}- or LNA-modified crRNA. Total counts are shown for sequences which passed computational filtering, as well as had Phred scores >30 at each position along the Cas9 target site and PAM. Mean mutations were calculated using the 20 nucleotides of the target site, as well as 2 of the 3 comprising the PAM (N of NGG was excluded). *P*-values were determined through comparison of 150 000 randomly sampled sequences of post-selection and corresponding pre-selection libraries using a Mann-Whitney test.

Supplementary Table 5. Statistics of cellular modification frequencies, sample size and *P*-values for high-throughput sequencing of Cas9:gRNA cleavage in U2OS-Cas9 and HeLa-Cas9 cells using no guide RNA, unmodified, BNA^{NC}- or LNA-modified crRNAs.

Cell Line	Site	crRNA	Indel	Total Counts	*P* Value
U2OS-Cas9	WAS	Control	7	135063	
U2OS-Cas9	WAS	WAS-RNA	74404	127421	$< 2.2 \times 10^{-16}$
U2OS-Cas9	WAS	WAS-BNA-3	19041	130670	$< 2.2 \times 10^{-16}$
U2OS-Cas9	WAS	WAS-LNA-3	66896	186551	$< 2.2 \times 10^{-16}$
U2OS-Cas9	WAS-OT1	Control	6	265507	
U2OS-Cas9	WAS-OT1	WAS-RNA	36671	208973	$< 2.2 \times 10^{-16}$
U2OS-Cas9	WAS-OT1	WAS-BNA-3	27173	289132	$< 2.2 \times 10^{-16}$
U2OS-Cas9	WAS-OT1	WAS-LNA-3	55509	200033	$< 2.2 \times 10^{-16}$
U2OS-Cas9	WAS-OT2	Control	0	151841	
U2OS-Cas9	WAS-OT2	WAS-RNA	84656	142152	$< 2.2 \times 10^{-16}$
U2OS-Cas9	WAS-OT2	WAS-BNA-3	77	226039	$< 2.2 \times 10^{-16}$
U2OS-Cas9	WAS-OT2	WAS-LNA-3	79930	226778	$< 2.2 \times 10^{-16}$
U2OS-Cas9	WAS-OT3	Control	0	120982	
U2OS-Cas9	WAS-OT3	WAS-RNA	89731	122681	$< 2.2 \times 10^{-16}$
U2OS-Cas9	WAS-OT3	WAS-BNA-3	1	196946	1
U2OS-Cas9	WAS-OT3	WAS-LNA-3	281	313339	$< 2.2 \times 10^{-16}$
U2OS-Cas9	WAS-OT4	Control	1	244429	
U2OS-Cas9	WAS-OT4	WAS-RNA	66337	181716	$< 2.2 \times 10^{-16}$
U2OS-Cas9	WAS-OT4	WAS-BNA-3	0	189146	1
U2OS-Cas9	WAS-OT4	WAS-LNA-3	560	154888	$< 2.2 \times 10^{-16}$
U2OS-Cas9	WAS-OT5	Control	2	216660	
U2OS-Cas9	WAS-OT5	WAS-RNA	218854	290880	$< 2.2 \times 10^{-16}$
U2OS-Cas9	WAS-OT5	WAS-BNA-3	0	232344	0.2328
U2OS-Cas9	WAS-OT5	WAS-LNA-3	58	177406	$< 2.2 \times 10^{-16}$

Cell Line	Site	crRNA	Indel	Total Counts	*P* Value
HeLa-Cas9	WAS	Control	4	175815	
HeLa-Cas9	WAS	WAS-RNA	130684	233282	$< 2.2 \times 10^{-16}$
HeLa-Cas9	WAS	WAS-BNA-3	35276	181138	$< 2.2 \times 10^{-16}$
HeLa-Cas9	WAS	WAS-LNA-3	52499	150807	$< 2.2 \times 10^{-16}$
HeLa-Cas9	WAS-OT1	Control	6	162391	
HeLa-Cas9	WAS-OT1	WAS-RNA	25450	140379	$< 2.2 \times 10^{-16}$
HeLa-Cas9	WAS-OT1	WAS-BNA-3	12276	140675	$< 2.2 \times 10^{-16}$
HeLa-Cas9	WAS-OT1	WAS-LNA-3	66605	246307	$< 2.2 \times 10^{-16}$
HeLa-Cas9	WAS-OT2	Control	1	200567	
HeLa-Cas9	WAS-OT2	WAS-RNA	88936	166572	$< 2.2 \times 10^{-16}$
HeLa-Cas9	WAS-OT2	WAS-BNA-3	0	196263	1
HeLa-Cas9	WAS-OT2	WAS-LNA-3	61926	205026	$< 2.2 \times 10^{-16}$
HeLa-Cas9	WAS-OT3	Control	4	225024	
HeLa-Cas9	WAS-OT3	WAS-RNA	113162	159701	$< 2.2 \times 10^{-16}$
HeLa-Cas9	WAS-OT3	WAS-BNA-3	7	149701	0.1292
HeLa-Cas9	WAS-OT3	WAS-LNA-3	266	172325	$< 2.2 \times 10^{-16}$
HeLa-Cas9	WAS-OT4	Control	0	220588	
HeLa-Cas9	WAS-OT4	WAS-RNA	73969	223128	$< 2.2 \times 10^{-16}$
HeLa-Cas9	WAS-OT4	WAS-BNA-3	2	196905	0.2224
HeLa-Cas9	WAS-OT4	WAS-LNA-3	114	291185	$< 2.2 \times 10^{-16}$
HeLa-Cas9	WAS-OT5	Control	2	201738	
HeLa-Cas9	WAS-OT5	WAS-RNA	207868	292282	$< 2.2 \times 10^{-16}$
HeLa-Cas9	WAS-OT5	WAS-BNA-3	4	212485	0.6881
HeLa-Cas9	WAS-OT5	WAS-LNA-3	5	330680	0.7167
U2OS-Cas9	EMX1	Control	6	146532	
U2OS-Cas9	EMX1	EMX1-RNA	112844	163114	$< 2.2 \times 10^{-16}$
U2OS-Cas9	EMX1	EMX1-BNA-5	78223	285556	$< 2.2 \times 10^{-16}$
U2OS-Cas9	EMX1	EMX1-LNA-5	86812	185446	$< 2.2 \times 10^{-16}$

Cell Line	Site	crRNA	Indel	Total Counts	*P* Value
U2OS-Cas9	EMX1-OT1	Control	6	121079	
U2OS-Cas9	EMX1-OT1	EMX1-RNA	16929	138393	$< 2.2 \times 10^{-16}$
U2OS-Cas9	EMX1-OT1	EMX1-BNA-5	3	167545	0.1789
U2OS-Cas9	EMX1-OT1	EMX1-LNA-5	8	158349	1
U2OS-Cas9	EMX1-OT2	Control	41	133703	
U2OS-Cas9	EMX1-OT2	EMX1-RNA	494	119068	$< 2.2 \times 10^{-16}$
U2OS-Cas9	EMX1-OT2	EMX1-BNA-5	45	199083	0.1865
U2OS-Cas9	EMX1-OT2	EMX1-LNA-5	24	180737	0.00099
U2OS-Cas9	EMX1-OT3	Control	50	73931	
U2OS-Cas9	EMX1-OT3	EMX1-RNA	0	98752	$< 2.2 \times 10^{-16}$
U2OS-Cas9	EMX1-OT3	EMX1-BNA-5	71	151126	0.05264
U2OS-Cas9	EMX1-OT3	EMX1-LNA-5	20	133801	1.558×10^{-9}
U2OS-Cas9	EMX1-OT4	Control	92	126743	
U2OS-Cas9	EMX1-OT4	EMX1-RNA	2	126081	$< 2.2 \times 10^{-16}$
U2OS-Cas9	EMX1-OT4	EMX1-BNA-5	4	159035	$< 2.2 \times 10^{-16}$
U2OS-Cas9	EMX1-OT4	EMX1-LNA-5	0	194305	$< 2.2 \times 10^{-16}$
U2OS-Cas9	EMX1-OT5	Control	71	166087	
U2OS-Cas9	EMX1-OT5	EMX1-RNA	56	163340	$< 2.2 \times 10^{-16}$
U2OS-Cas9	EMX1-OT5	EMX1-BNA-5	40	173485	1
U2OS-Cas9	EMX1-OT5	EMX1-LNA-5	72	151924	$< 2.2 \times 10^{-16}$
HeLa-Cas9	EMX1	Control	64	203514	
HeLa-Cas9	EMX1	EMX1-RNA	126491	183008	$< 2.2 \times 10^{-16}$
HeLa-Cas9	EMX1	EMX1-BNA-5	46803	182476	$< 2.2 \times 10^{-16}$
HeLa-Cas9	EMX1	EMX1-LNA-5	96279	199447	$< 2.2 \times 10^{-16}$
HeLa-Cas9	EMX1-OT1	Control	12	135311	
HeLa-Cas9	EMX1-OT1	EMX1-RNA	16999	145571	$< 2.2 \times 10^{-16}$
HeLa-Cas9	EMX1-OT1	EMX1-BNA-5	7	148785	0.2503
HeLa-Cas9	EMX1-OT1	EMX1-LNA-5	2	106278	0.02969

Cell Line	Site	crRNA	Indel	Total Counts	*P* Value
HeLa-Cas9	EMX1-OT2	Control	42	167364	
HeLa-Cas9	EMX1-OT2	EMX1-RNA	328	144454	$< 2.2 \times 10^{-16}$
HeLa-Cas9	EMX1-OT2	EMX1-BNA-5	29	145830	0.3442
HeLa-Cas9	EMX1-OT2	EMX1-LNA-5	37	165871	0.6532
HeLa-Cas9	EMX1-OT3	Control	60	111185	
HeLa-Cas9	EMX1-OT3	EMX1-RNA	2	138824	0.1505
HeLa-Cas9	EMX1-OT3	EMX1-BNA-5	0	127723	0.01016
HeLa-Cas9	EMX1-OT3	EMX1-LNA-5	0	123049	0.01144
HeLa-Cas9	EMX1-OT4	Control	50	119940	
HeLa-Cas9	EMX1-OT4	EMX1-RNA	20	129032	0.0001025
HeLa-Cas9	EMX1-OT4	EMX1-BNA-5	82	160850	0.2911
HeLa-Cas9	EMX1-OT4	EMX1-LNA-5	55	153363	0.4914
HeLa-Cas9	EMX1-OT5	Control	90	151920	
HeLa-Cas9	EMX1-OT5	EMX1-RNA	26	137555	4.024×10^{-11}
HeLa-Cas9	EMX1-OT5	EMX1-BNA-5	21	138296	$< 2.2 \times 10^{-16}$
HeLa-Cas9	EMX1-OT5	EMX1-LNA-5	82	148466	$< 2.2 \times 10^{-16}$

Statistics are shown for cellular modification frequencies following transfection with unmodified, BNA^{NC}- or LNA-modified crRNAs into stably expressing Cas9 cells. Controls were performed using no guide RNA during transfection. Indels are defined as the number of observed sequences containing insertions or deletions which are consistent with Cas9-mediated cleavage. Total number of sequences for each library is reported as the number of reads following high-throughput sequencing which were successfully mapped to a reference amplicon and having a mean Phred score >30. *P*-values were calculated using a Fisher's exact test between each Cas9-treated sample and the corresponding untreated control. The sensitivity of high-throughput sequencing

for the detection of genomic off-target cleavage is limited by the amount of genomic DNA (gDNA) used as input[2]. Each sample was run with 100 ng of gDNA, which is equivalent to ~33 000 genomes. Therefore, the theoretical detection limit of this technique is ~1 in 33 000, which we have indicated as < 0.003%.

Supplementary Table 6. *In vitro* cleavage assay data used to generate Supplementary Fig. 16.

	crRNA	Target WAS	WAS-OT1	WAS-OT2	WAS-OT3	WAS-OT4	WAS-OT5
150 nM RNP	**WAS-LNA-3**	79 ± 1.5	87 ± 0.92	85 ± 0.04	22 ± 1.2	66 ± 1.4	4.5 ± 0.61
	**WAS-LNA-5**	87 ± 7.3	98 ± 0.08	99 ± 0.03	55 ± 9.3	93 ± 3.8	45 ± 7.3
	**WAS-LNA-6**	56 ± 6.4	65 ± 8.8	59 ± 8.0	0.0 ± 0.0	24 ± 4.6	0.0 ± 0.0
15 nM RNP	**WAS-LNA-3**	62 ± 0.17	57 ± 11	75 ± 0.62	15 ± 1.0	53 ± 3.3	2.5 ± 0.70
	**WAS-LNA-5**	51 ± 5.1	34 ± 7.4	65 ± 17	52 ± 23	10 ± 10	3.2 ± 3.2
	**WAS-LNA-6**	48 ± 7.9	54 ± 8.5	67 ± 5.3	0.0 ± 0.0	17 ± 7.6	0.0 ± 0.0

	crRNA	Target EMX1	EMX1-OT1	EMX1-OT2	EMX1-OT3	EMX1-OT4	EMX1-OT5
150 nM RNP	**EMX1-LNA-5**	49 ± 13	0.0 ± 0.0	0.0 ± 0.0	0.0 ± 0.0	9.7 ± 9.7	0.0 ± 0.0
	**EMX1-LNA-6**	64 ± 0.20	0.5 ± 0.5	16 ± 0.61	18 ± 1.1	65 ± 1.0	0.0 ± 0.0
	**EMX1-LNA-7**	93 ± 0.37	56 ± 4.7	76 ± 1.3	56 ± 0.91	43 ± 1.2	71 ± 3.7
15 nM RNP	**EMX1-LNA-5**	43 ± 6.7	0.0 ± 0.0	0.0 ± 0.0	0.0 ± 0.0	0.0 ± 0.0	0.0 ± 0.0
	**EMX1-LNA-6**	47 ± 1.5	0.0 ± 0.0	2.9 ± 2.9	0.0 ± 0.0	22 ± 0.44	5.7 ± 0.11
	**EMX1-LNA-7**	70 ± 15	70 ± 3.4	44 ± 1.1	5.3 ± 0.07	3.0 ± 0.71	19 ± 1.8

Values showing *in vitro* cleavage specificity for unmodified crRNA and 3 LNA-modified crRNAs towards either WAS (top) or EMX1 (bottom) on- and off-target sequences (as listed **Fig. 1b**, **c**); Mean ± SE (n = 2). Experiments were performed using 150 nM or 15 nM Cas9 RNP and 5 nM DNA.

Supplementary Table 7: Cellular modification rates induced by unmodified or LNA-modified crRNAs targeting *WAS* or *EMX1*.

Cell Type	U2OS-Cas9	U2OS-Cas9	U2OS-Cas9	HeLa-Cas9	HeLa-Cas9	HeLa-Cas9
Treatment	No gRNA	WAS-RNA	WAS-LNA-3	No gRNA	WAS-RNA	WAS-LNA-3
WAS	< 0.003	58.392	35.859	< 0.003	56.020	34.812
WAS-OT1	0.003	17.548	27.750	0.004	18.135	27.041
WAS-OT2	0.092	59.553	35.246	0.120	53.400	30.204
WAS-OT3	< 0.003	73.142	0.090	< 0.003	70.859	0.154
WAS-OT4	< 0.003	36.506	0.362	< 0.003	33.151	0.383
WAS-OT5	< 0.003	75.239	0.032	< 0.003	71.119	< 0.003

Cell Type	U2OS-Cas9	U2OS-Cas9	U2OS-Cas9	HeLa-Cas9	HeLa-Cas9	HeLa-Cas9
Treatment	No gRNA	EMX1-RNA	EMX1-LNA-5	No gRNA	EMX1-RNA	EMX1-LNA-5
EMX1	0.004	69.181	46.813	< 0.003	69.118	48.273
EMX1-OT1	0.005	12.233	< 0.003	0.009	11.677	< 0.003
EMX1-OT2	0.031	1.007	0.013	0.025	0.607	0.022
EMX1-OT3	0.068	< 0.003	0.015	0.005	< 0.003	< 0.003
EMX1-OT4	0.073	< 0.003	< 0.003	0.042	0.016	0.036
EMX1-OT5	< 0.003	0.113	0.059	0.060	0.141	< 0.003

Table summarizing modification frequencies of on- and off-target sequences in U2OS-Cas9 and HeLa-Cas9 cells using either unmodified or LNA-modified crRNAs targeting *WAS* or *EMX1*, as determined by high-throughput sequencing. Modification frequencies were calculated by dividing the number of sequences bearing insertions or deletions (indels) in the target site by the total number of sequences. Mock transfections lacking gRNA were used as controls (See **Supplementary Table 5** for additional data). No gRNA, WAS-RNA and EMX1-RNA values from **Tables 1** and **2** are shown again as reference.

Supplementary Table 8: Unmodified, BNA^{NC}- and LNA-modified crRNA and tracrRNA sequences used.

Name	Sequence (5′ -> 3′)
WAS-RNA crRNA	rUrGrGrArUrGrGrArGrGrArArUrGrArGrGrArGrUrGrUrUrUrUrArGrArGrCrUrArUrGrCrU
WAS-BNA-1 crRNA	rUrGrGrArUrGrGrArGrG+ArArUrGrArGrGrArGrUrGrUrUrUrUrArGrArGrCrUrArUrGrCrU
WAS-BNA-2 crRNA	rUrGrGrArUrGrGrArG+G+ArArUrGrArGrGrArGrUrGrUrUrUrUrArGrArGrCrUrArUrGrCrU
WAS-BNA-3 crRNA	rUrGrGrArUrGrGrArG+G+A+ArUrGrArGrGrArGrUrGrUrUrUrUrArGrArGrCrUrArUrGrCrU
WAS-BNA-4 crRNA	rUrGrGrArUrGrGrArGrGrArArUrGrArGrG+A+G+TrGrUrUrUrUrArGrArGrCrUrArUrGrCrU
WAS-BNA-5 crRNA	rUrGrGrArUrGrGrArGrGrArArU+G+A+GrGrArGrUrGrUrUrUrUrArGrArGrCrUrArUrGrCrU
WAS-BNA-6 crRNA	rUrGrGrArUrGrGrArG+G+A+A+TrGrArGrGrArGrUrGrUrUrUrUrArGrArGrCrUrArUrGrCrU
WAS-BNA-7 crRNA	rUrGrGrArUrGrGrA+G+GrA+A+TrGrArGrGrArGrUrGrUrUrUrUrArGrArGrCrUrArUrGrCrU
WAS-BNA-8 crRNA	rUrGrG+A+T+GrGrArGrGrArArU+G+A+GrGrArGrUrGrUrUrUrUrArGrArGrCrUrArUrGrCrU
WAS-BNA-9 crRNA	+T+GrG+A+T+GrGrArGrGrArArU+G+A+GrGrArGrUrGrUrUrUrUrArGrArGrCrUrArUrGrCrU
WAS-LNA-3 crRNA	rUrGrGrArUrGrGrArG*G*A*ArUrGrArGrGrArGrUrGrUrUrUrUrArGrArGrCrUrArUrGrCrU
WAS-LNA-5 crRNA	rUrGrGrArUrGrGrArGrGrArArU*G*A*GrGrArGrUrGrUrUrUrUrArGrArGrCrUrArUrGrCrU
WAS-LNA-6 crRNA	rUrGrGrArUrGrGrArG*G*A*A*TrGrArGrGrArGrUrGrUrUrUrUrArGrArGrCrUrArUrGrCrU
EMX1-RNA crRNA	rGrArGrUrCrCrGrArGrCrArGrArArGrArArGrArArGrUrUrUrUrArGrArGrCrUrArUrGrCrU
EMX1-BNA-1 crRNA	rGrArGrUrCrCrGrArGrCrArGrArArGrArArG+A+ArGrUrUrUrUrArGrArGrCrUrArUrGrCrU
EMX1-BNA-2 crRNA	rGrArGrUrCrCrGrArGrCrArGrArA+G+A+ArGrArArGrUrUrUrUrArGrArGrCrUrArUrGrCrU
EMX1-BNA-3 crRNA	+G+A+GrUrCrCrGrArGrCrArGrArArGrArArGrArArGrUrUrUrUrArGrArGrCrUrArUrGrCrU
EMX1-BNA-4 crRNA	rGrArGrUrCrCrGrArG+C+A+GrArArGrArArGrArArGrUrUrUrUrArGrArGrCrUrArUrGrCrU
EMX1-BNA-5 crRNA	rGrArGrUrCrCrGrArGrCrA+G+A+ArGrArArGrArArGrUrUrUrUrArGrArGrCrUrArUrGrCrU
EMX1-BNA-6 crRNA	rGrArGrUrCrCrGrArG+CrA+GrA+ArGrArArGrArArGrUrUrUrUrArGrArGrCrUrArUrGrCrU
EMX1-BNA-7 crRNA	rGrArGrUrCrCrGrArGrCrArGrArArGrA+A+G+A+ArGrUrUrUrUrArGrArGrCrUrArUrGrCrU

Name	Sequence (5′ -> 3′)
EMX1-BNA-8 crRNA	rGrArGrU+C+C+G+ArGrCrArGrArArGrArArGrArArGrUrUrUrArGrArGrCrUrArUrGrCrU
EMX1-BNA-9 crRNA	rGrArGrUrCrCrGrArGrC+A+GrArArGrArA+G+A+ArGrUrUrUrArGrArGrCrUrArUrGrCrU
EMX1-LNA-5 crRNA	rGrArGrUrCrCrGrArGrCrA*G*A*ArGrArArGrArArGrUrUrUrArGrArGrCrUrArUrGrCrU
EMX1-LNA-6 crRNA	rGrArGrUrCrCrGrArG*CrA*GrA*ArGrArArGrArArGrUrUrUrArGrArGrCrUrArUrGrCrU
EMX1-LNA-7 crRNA	rGrArGrUrCrCrGrArGrCrArGrArArGrA*A*G*A*ArGrUrUrUrArGrArGrCrUrArUrGrCrU
WAS-RNA Cy5 crRNA	[Cy5]rUrGrGrArUrGrGrArGrGrArArUrGrArGrGrArGrUrGrUrUrUrArGrArGrCrUrArUrGrCrU
WAS-BNA-3 Cy5 crRNA	[Cy5]rUrGrGrArUrGrGrArG+G+A+ArUrGrArGrGrArGrUrGrUrUrUrArGrArGrCrUrArUrGrCrU
tracrRNA	rArGrCrArUrArGrCrArArGrUrUrArArArArUrArArGrGrCrUrArGrUrCrCrGrUrUrArUrCrArArCrUrUrGrArArArArArGrUrGrGrCrArCrCrGrArGrUrCrGrGrUrGrCrUrUrU

A plus sign (+) indicates that the following nucleotide is a BNA^{NC}, while an asterisks (*) indicates the following nucleotide is a LNA. Nucleotides with a preceding (r) were ordered as RNA.

Supplementary Table 9: Oligonucleotide sequences used in this study.

Name	Sequence (5′ -> 3′)
	The following were sequences used to generate in vitro cleavage assay constructs
WAS_target#	GGCCGGCCGAAGCTTAGGAGGTGCGTGCTGATTCTTCCCTGTGTCTCTGGATGGATGGGTAAGAGTGGATGGAGGAATGAGGAGTTGGATGGGTGCGTAAGTGGGTGAATGGATAGGTAGATTGATAGGTATGTGGATTCTAGAGGCCGGCCG
WAS-OT1_target#	GGCCGGCCGAAGCTTCCATCCTTAGCACAGAGTTTGGCACATAGAGGAGCCTGATCAATACCTGCTGGATGGAGGGATGAGGAGTGGGGCTGGGCCGTATCAGTCAGGATGGTGAAGTGCATAACAAACAGCCCCCACTCTAGAGGCCGGCCG
WAS-OT2_target#	GGCCGGCCGAAGCTTACATTATTTATGCTAGGAAACCCTGTTGCTGTATGATTTGTGTGTGTGAGGGGATGGAGGGATGAGGAGTGGGAAGCTGTTGACTCATGCACATACCTGTCTCCATGGTGTCTCTGCTGTCGATCTAGAGGCCGGCCG
WAS-OT3_target#	GGCCGGCCGAAGCTTTGTAGAGTAGTCAAATTCACAGGGACAGAAAGTAGAATAGTGGTTGCTGGGGGAGGGAGGAATGAGGAGTGGGTACAGAGTTTCATCTGGGGAAGATGAAAAAGTTCTGTAGACGGATGGTGGTCTAGAGGCCGGCCG
WAS-OT4_target#	GGCCGGCCGAAGCTTAGTAGGGTTAGGCCACGACACTCAGGCTTTCAGGACAAACAAGGAGAGGGAGGAGGGAGGAATGGGGAGTTGGTAGTTAATGGGGATGGAGTTTCAGTTTGGGATGACAAAAAAGTTCTGGAGTCTAGAGGCCGGCCG
WAS-OT5_target#	GGCCGGCCGAAGCTTGCACTGGTCACCTGTTTGAGGAAAATGGACTGTCTCCAAATTGCCCCACTCGGACGGAGGAATGGGGAGTGGGGGCTGGTTTGTGAAGGCTGTTCTCTTTCTAAAGTCTCAGTCTCTCTTTTCTCTAGAGGCCGGCCG
EMX1_target_fwd	GCCGAAGCTTCTGAGTCCGAGCAGAAGAAGAAGGGCTTCTAGAGGCC
EMX1_target_rev	GGCCTCTAGAAGCCCTTCTTCTTCTGCTCGGACTCAGAAGCTTCGGC
EMX1-OT1_target_fwd	GCCGAAGCTTCTGAGTTAGAGCAGAAGAAGAAAGGCTTCTAGAGGCC
EMX1-OT1_target_rev	GGCCTCTAGAAGCCTTTCTTCTTCTGCTCTAACTCAGAAGCTTCGGC
EMX1-OT2_target_fwd	GCCGAAGCTTCTGAGTCTAAGCAGAAGAAGAAGAGCTTCTAGAGGCC
EMX1-OT2_target_rev	GGCCTCTAGAAGCTCTTCTTCTTCTGCTTAGACTCAGAAGCTTCGGC
EMX1-OT3_target_fwd	GCCGAAGCTTCTGAGGCCGAGCAGAAGAAAGACGGCTTCTAGAGGCC
EMX1-OT3_target_rev	GGCCTCTAGAAGCCGTCTTTCTTCTGCTCGGCCTCAGAAGCTTCGGC
EMX1-OT4_target_fwd	GCCGAAGCTTCTGAGTCCTAGCAGGAGAAGAAGAGCTTCTAGAGGCC
EMX1-OT4_target_rev	GGCCTCTAGAAGCTCTTCTTCTCCTGCTAGGACTCAGAAGCTTCGGC

**Name**	**Sequence (5′ -> 3′)**
EMX1-OT5_target_fwd	GCCGAAGCTTCTAAGTCTGAGCACAAGAAGAATGGCTTCTAGAGGCC
EMX1-OT5_target_rev	GGCCTCTAGAAGCCATTCTTCTTGTGCTCAGACTTAGAAGCTTCGGC
WAS-m1 target_fwd	GCCGAAGCTTCTTGGATGGAGGAATGAGGAGAGGGCTTCTAGAGGCC
WAS-m1 target_rev	GGCCTCTAGAAGCCCTCTCCTCATTCCTCCATCCAAGAAGCTTCGGC
WAS-m2 target_fwd	GCCGAAGCTTCTTGGATGGAGGAATGAGGGGTGGGCTTCTAGAGGCC
WAS-m2 target_rev	GGCCTCTAGAAGCCCACCCCTCATTCCTCCATCCAAGAAGCTTCGGC
WAS-m3 target_fwd	GCCGAAGCTTCTTGGATGGAGGAATGACGAGTGGGCTTCTAGAGGCC
WAS-m3 target_rev	GGCCTCTAGAAGCCCACTCGTCATTCCTCCATCCAAGAAGCTTCGGC
WAS-m4 target_fwd	GCCGAAGCTTCTTGGATGGAGGAAAGAGGAGTGGGCTTCTAGAGGCC
WAS-m4 target_rev	GGCCTCTAGAAGCCCACTCCTCTTTCCTCCATCCAAGAAGCTTCGGC
WAS-m5 target_fwd	GCCGAAGCTTCTTGGATGGAGGACTGAGGAGTGGGCTTCTAGAGGCC
WAS-m5 target_rev	GGCCTCTAGAAGCCCACTCCTCAGTCCTCCATCCAAGAAGCTTCGGC
WAS-m6 target_fwd	GCCGAAGCTTCTTGGATGGAGAAATGAGGAGTGGGCTTCTAGAGGCC
WAS-m7 target_rev	GGCCTCTAGAAGCCCACTCCTCATTTCTCCATCCAAGAAGCTTCGGC
WAS-m7 target_fwd	GCCGAAGCTTCTTGGATGGTGGAATGAGGAGTGGGCTTCTAGAGGCC
WAS-m7 target_rev	GGCCTCTAGAAGCCCACTCCTCATTCCACCATCCAAGAAGCTTCGGC
WAS-m8 target_fwd	GCCGAAGCTTCTTGGATTGAGGAATGAGGAGTGGGCTTCTAGAGGCC
WAS-m8 target_rev	GGCCTCTAGAAGCCCACTCCTCATTCCTCAATCCAAGAAGCTTCGGC
WAS-m9 target_fwd	GCCGAAGCTTCTTGGGTGGAGGAATGAGGAGTGGGCTTCTAGAGGCC
WAS-m9 target_rev	GGCCTCTAGAAGCCCACTCCTCATTCCTCCACCCAAGAAGCTTCGGC
WAS-m10 target_fwd	GCCGAAGCTTCTTCGATGGAGGAATGAGGAGTGGGCTTCTAGAGGCC
WAS-m10 target_rev	GGCCTCTAGAAGCCCACTCCTCATTCCTCCATCGAAGAAGCTTCGGC
WAS-m11 target_fwd	GCCGAAGCTTCTAGGATGGAGGAATGAGGAGTGGGCTTCTAGAGGCC
WAS-m11 target_rev	GGCCTCTAGAAGCCCACTCCTCATTCCTCCATCCTAGAAGCTTCGGC
EMX1-m1 target_fwd	GCCGAAGCTTCTGAGTCCGAGCAGAAGAAGAGGGGCTTCTAGAGGCC

Name	Sequence (5′ -> 3′)
EMX1-m1 target_rev	GGCCTCTAGAAGCCCCTCTTCTTCTGCTCGGACTCAGAAGCTTCGGC
EMX1-m2 target_fwd	GCCGAAGCTTCTGAGTCCGAGCAGAAGAATAAGGGCTTCTAGAGGCC
EMX1-m2 target_rev	GGCCTCTAGAAGCCCTTATTCTTCTGCTCGGACTCAGAAGCTTCGGC
EMX1-m3 target_fwd	GCCGAAGCTTCTGAGTCCGAGCAGAAGGAGAAGGGCTTCTAGAGGCC
EMX1-m3 target_rev	GGCCTCTAGAAGCCCTTCTCCTTCTGCTCGGACTCAGAAGCTTCGGC
EMX1-m4 target_fwd	GCCGAAGCTTCTGAGTCCGAGCAGAGGAAGAAGGGCTTCTAGAGGCC
EMX1-m4 target_rev	GGCCTCTAGAAGCCCTTCTTCCTCTGCTCGGACTCAGAAGCTTCGGC
EMX1-m5 target_fwd	GCCGAAGCTTCTGAGTCCGGGCAGAAGAAGAAGGGCTTCTAGAGGCC
EMX1-m5 target_rev	GGCCTCTAGAAGCCCTTCTTCTTCTGCCCGGACTCAGAAGCTTCGGC
EMX1-m6 target_fwd	GCCGAAGCTTCTGAGTCCGAGTAGAAGAAGAAGGGCTTCTAGAGGCC
EMX1-m6 target_rev	GGCCTCTAGAAGCCCTTCTTCTTCTACTCGGACTCAGAAGCTTCGGC
EMX1-m7 target_fwd	GCCGAAGCTTCTGAGTCCGGGCAGAAGAAGAAGGGCTTCTAGAGGCC
EMX1-m7 target_rev	GGCCTCTAGAAGCCCTTCTTCTTCTGCCCGGACTCAGAAGCTTCGGC
EMX1-m8 target_fwd	GCCGAAGCTTCTGAGTCTGAGCAGAAGAAGAAGGGCTTCTAGAGGCC
EMX1-m8 target_rev	GGCCTCTAGAAGCCCTTCTTCTTCTGCTCAGACTCAGAAGCTTCGGC
EMX1-m9 target_fwd	GCCGAAGCTTCTGAGGCCGAGCAGAAGAAGAAGGGCTTCTAGAGGCC
EMX1-m9 target_rev	GGCCTCTAGAAGCCCTTCTTCTTCTGCTCGGCCTCAGAAGCTTCGGC
EMX1-m10 target_fwd	GCCGAAGCTTCTGCGTCCGAGCAGAAGAAGAAGGGCTTCTAGAGGCC
EMX1-m10 target_rev	GGCCTCTAGAAGCCCTTCTTCTTCTGCTCGGACGCAGAAGCTTCGGC
EMX1-m11 target_fwd	GCCGAAGCTTCTTAGTCCGAGCAGAAGAAGAAGGGCTTCTAGAGGCC
EMX1-m11 target_rev	GGCCTCTAGAAGCCCTTCTTCTTCTGCTCGGACTAAGAAGCTTCGGC
pUC19_fwd	GCGACACGGAAATGTTGAATACTCAT
pUC19_rev	CAGCGAGTCAGTGAGCGA
	The following sequences were used for in vitro library selection experiments
WAS_lib	/5Phos/AACACANNNNC*C*NA*C*T*C*C*T*C*A*T*T*C*C*T*C*C*A*T*C*C*A*NNNNACCTG CCGAGAACACA

Name	Sequence (5′ -> 3′)
EMX1_lib	/5Phos/TCTTCTNNNNC*C*NT*T*C*T*T*C*T*T*C*T*G*C*T*C*G*G*A*C*T*C*NN NNACCTGCCGAGTCTTCT
adaptor1(1)	AAT GAT ACG GCG ACC ACC GAG ATC TAC ACT CTT TCC CTA CAC GAC GCT CTT CCG ATC TAC TGT
adaptor1(2)	AAT GAT ACG GCG ACC ACC GAG ATC TAC ACT CTT TCC CTA CAC GAC GCT CTT CCG ATC TCT GAA
adaptor1(3)	AAT GAT ACG GCG ACC ACC GAG ATC TAC ACT CTT TCC CTA CAC GAC GCT CTT CCG ATC TTG ACT
adaptor1(4)	AAT GAT ACG GCG ACC ACC GAG ATC TAC ACT CTT TCC CTA CAC GAC GCT CTT CCG ATC TTG CAA
adaptor1(5)	AAT GAT ACG GCG ACC ACC GAG ATC TAC ACT CTT TCC CTA CAC GAC GCT CTT CCG ATC TGC ATT
adaptor1(6)	AAT GAT ACG GCG ACC ACC GAG ATC TAC ACT CTT TCC CTA CAC GAC GCT CTT CCG ATC TCA TGA
adaptor1(7)	AAT GAT ACG GCG ACC ACC GAG ATC TAC ACT CTT TCC CTA CAC GAC GCT CTT CCG ATC TAT GCT
adaptor1(8)	AAT GAT ACG GCG ACC ACC GAG ATC TAC ACT CTT TCC CTA CAC GAC GCT CTT CCG ATC TCT AGT
adaptor1(9)	AAT GAT ACG GCG ACC ACC GAG ATC TAC ACT CTT TCC CTA CAC GAC GCT CTT CCG ATC TGC TAA
adaptor1(10)	AAT GAT ACG GCG ACC ACC GAG ATC TAC ACT CTT TCC CTA CAC GAC GCT CTT CCG ATC TCA GTT
adaptor1(11)	AAT GAT ACG GCG ACC ACC GAG ATC TAC ACT CTT TCC CTA CAC GAC GCT CTT CCG ATC TGT CAT
adaptor1(12)	AAT GAT ACG GCG ACC ACC GAG ATC TAC ACT CTT TCC CTA CAC GAC GCT CTT CCG ATC TAC GTA
adaptor2(1)	ACA GTA GAT CGG AAG AGC GTC GTG TAG GGA AAG AGT GTA GAT CTC GGT GG
adaptor2(2)	TTC AGA GAT CGG AAG AGC GTC GTG TAG GGA AAG AGT GTA GAT CTC GGT GG
adaptor2(3)	AGT CAA GAT CGG AAG AGC GTC GTG TAG GGA AAG AGT GTA GAT CTC GGT GG
adaptor2(4)	TTG CAA GAT CGG AAG AGC GTC GTG TAG GGA AAG AGT GTA GAT CTC GGT GG
adaptor2(5)	AAT GCA GAT CGG AAG AGC GTC GTG TAG GGA AAG AGT GTA GAT CTC GGT GG
adaptor2(6)	TCA TGA GAT CGG AAG AGC GTC GTG TAG GGA AAG AGT GTA GAT CTC GGT GG
adaptor2(7)	AGC ATA GAT CGG AAG AGC GTC GTG TAG GGA AAG AGT GTA GAT CTC GGT GG
adaptor2(8)	ACT AGA GAT CGG AAG AGC GTC GTG TAG GGA AAG AGT GTA GAT CTC GGT GG
adaptor2(9)	TTA GCA GAT CGG AAG AGC GTC GTG TAG GGA AAG AGT GTA GAT CTC GGT GG
adaptor2(10)	AAC TGA GAT CGG AAG AGC GTC GTG TAG GGA AAG AGT GTA GAT CTC GGT GG
adaptor2(11)	ATG ACA GAT CGG AAG AGC GTC GTG TAG GGA AAG AGT GTA GAT CTC GGT GG
adaptor2(12)	TAC GTA GAT CGG AAG AGC GTC GTG TAG GGA AAG AGT GTA GAT CTC GGT GG

Name	Sequence (5′ -> 3′)
PE2_short	AAT GAT ACG GCG ACC ACC GA
WAS_sel PCR	CAA GCA GAA GAC GGC ATA CGA GAT ACC TGC CGA GAA CAC A
EMX1_sel PCR	CAA GCA GAA GAC GGC ATA CGA GAT ACC TGC CGA GTC TTC T
lib adaptor 1	GAC GGC ATA CGA GAT
WAS lib adaptor 2	AAC AAT CTC GTA TGC CGT CTT CTG CTT G
EMX1 lib adaptor 2	TCT TAT CTC GTA TGC CGT CTT CTG CTT G
WAS lib seq PCR	AAT GAT ACG GCG ACC ACC GAG ATC TAC ACT CTT TCC CTA CAC GAC GCT CTT CCG ATC TNN NNA CCT ACC TGC CGA GAA CAC A
EMX1 lib seq PCR	AAT GAT ACG GCG ACC ACC GAG ATC TAC ACT CTT TCC CTA CAC GAC GCT CTT CCG ATC TNN NNA CCT ACC TGC CGA GTC TTC T
lib fwd PCR	CAA GCA GAA GAC GGC ATA CGA GAT
	The following sequences were used for EMSA experiments
WAS_EMSA fwd	CCCATCCAACTCCTCATTCCTCCATCCA
WAS_EMSA rev	TGGATGGAGGAATGAGGAGTTGGATGGGCGC[6-FAM]
WAS-OT3_EMSA fwd	CTGTACCCACTCCTCATTCCTCCCTCCC
WAS-OT3_EMSA rev	GGGAGGGAGGAATGAGGAGTGGGTACAGCGC[6-FAM]
EMX1_EMSA fwd	GGGAGCCCTTCTTCTTCTGCTCGGACTC
EMX1_EMSA rev	GAGTCCGAGCAGAAGAAGAAGGGCTCCCCGC[6-FAM]
EMX1-OT1_EMSA fwd	CCATGCCTTTCTTCTTCTGCTCTAACTC
EMX1-OT1_EMSA rev	GAGTTAGAGCAGAAGAAGAAAGGCATGGCGC[6-FAM]
	The following sequences were used for Tm determination experiments
WAS_Tm	TCCAACTCCTCATTCCTCCATCCAC
WAS-OT1_Tm	TCCAACTCCTCATCCCTCCATCCAC
WAS-OT2_Tm	TCCCACTCCTCATCCCTCCATCCCC
WAS-OT3_Tm	ACCCACTCCTCATTCCTCCCTCCCC
WAS-OT4_Tm	ACCAACTCCCCATTCCTCCCTCCTC
WAS-OT5_Tm	CCCCACTCCCCATTCCTCCGTCCGA
	The following sequences were used for cellular cleavage experiments

Name	Sequence (5′ -> 3′)
WAS_T7E1 fwd	GTGGCAGGGCTGTGATAACT
WAS_T7E1 rev	TGCTTTATCATTCACTGGCTCA
WAS-OT3_T7E1 fwd	GCTACAACATGGATGAACCTGGA
WAS-OT3_T7E1 rev	GCCTCCCAAAGTGCTGAGAT
EMX1_T7E1 fwd	CCTCAGTCTTCCCATCAGC
EMX1_T7E1 rev	GTGGGGCCATGACTCCAG
EMX1-OT1_T7E1 fwd	TGCCTTTCATCTGATGCTGT
EMX1-OT1_T7E1 rev	GGTGCCTTTTGTGGGGAGAT
WAS PCR1_fwd	TCGTCGGCAGCGTCAGATGTGTATAAGAGACAGCCAGAGAGAAGAGGAGGGCT
WAS PCR1_rev	GTCTCGTGGGCTCGGAGATGTGTATAAGAGACAGCCACCCATCCATCAGTCCAC
WAS-OT1 PCR1_fwd	TCGTCGGCAGCGTCAGATGTGTATAAGAGACAGTTCTGCAGTCAAGAGCCTCG
WAS-OT1 PCR1_rev	GTCTCGTGGGCTCGGAGATGTGTATAAGAGACAGGGCTTTGCCTGGGAGAGAAA
WAS-OT2 PCR1_fwd	TCGTCGGCAGCGTCAGATGTGTATAAGAGACAGGCCAGCCTAAATCCCACCTT
WAS-OT2 PCR1_rev	GTCTCGTGGGCTCGGAGATGTGTATAAGAGACAGTGAGCCCCCTCCATTGGATA
WAS-OT3 PCR1_fwd	TCGTCGGCAGCGTCAGATGTGTATAAGAGACAGGCTACAACATGGATGAACCTGGA
WAS-OT3 PCR1_rev	GTCTCGTGGGCTCGGAGATGTGTATAAGAGACAGAACCATCACCACCATCCGTC
WAS-OT4 PCR1_fwd	TCGTCGGCAGCGTCAGATGTGTATAAGAGACAGAGCATGAAGGGAAGAGGTGC
WAS-OT4 PCR1_rev	GTCTCGTGGGCTCGGAGATGTGTATAAGAGACAGTCCTATGCAACCATCACCACC
WAS-OT5 PCR1_fwd	TCGTCGGCAGCGTCAGATGTGTATAAGAGACAGGTCTCAACTGCCTGCTCTGG
WAS-OT5 PCR1_rev	GTCTCGTGGGCTCGGAGATGTGTATAAGAGACAGGAGGGGCAGGAGACACAGA
EMX1 PCR1_fwd	TCGTCGGCAGCGTCAGATGTGTATAAGAGACAGGGGCCTCCTGAGTTTCTCATC
EMX1 PCR1_rev	GTCTCGTGGGCTCGGAGATGTGTATAAGAGACAGTGCCCACCCTAGTCATTGGA
EMX1-OT1 PCR1_fwd	TCGTCGGCAGCGTCAGATGTGTATAAGAGACAGGGGCTTATGGCATGGCAAGA
EMX1-OT1 PCR1_rev	GTCTCGTGGGCTCGGAGATGTGTATAAGAGACAGGCAAGCTTTTCCTGACGCC

Name	Sequence (5′ -> 3′)
EMX1-OT2 PCR1_fwd	TCGTCGGCAGCGTCAGATGTGTATAAGAGACAGTCACCTGGGCGAGAAAGG TA
EMX1-OT2 PCR1_rev	GTCTCGTGGGCTCGGAGATGTGTATAAGAGACAGTGGCTTTCACAAGGATG CAGT
EMX1-OT3 PCR1_fwd	TCGTCGGCAGCGTCAGATGTGTATAAGAGACAG GTGGGAGAGAGACCCCTTCT
EMX1-OT3 PCR1_rev	GTCTCGTGGGCTCGGAGATGTGTATAAGAGACAG TCATCCCGACCTTCATCCCT
EMX1-OT4 PCR1_fwd	TCGTCGGCAGCGTCAGATGTGTATAAGAGACAGCTTCTTTGGGCCTCGGCTT
EMX1-OT4 PCR1_rev	GTCTCGTGGGCTCGGAGATGTGTATAAGAGACAGGGCCCAGCTGTGCATTC TAT
EMX1-OT5 PCR1_fwd	TCGTCGGCAGCGTCAGATGTGTATAAGAGACAGCCAGAAGCACCCGGATGT AG
EMX1-OT5 PCR1_rev	GTCTCGTGGGCTCGGAGATGTGTATAAGAGACAGTCAAACAAGGTGCAGAT ACAGCA
	The following sequences were used for single-molecule FRET experiments
WAS smFRET	/5Biosg/ttt ttt GAG GAA G/iAmMC6T/G CCT TGG ATG GAG GAA TGA GGA GTT GGC TCC CAT CAC ATC
WAS-OT4 smFRET	/5Biosg/ttt ttt GAG GAA G/iAmMC6T/G CCT AGG AGG GAG GAA TGG GGA GTT GGC TCC CAT CAC ATC

Sequence names ending with a number sign (#) were ordered from IDT as dsDNA gBlocks. An asterisks (*) indicates that the preceding nucleotide was incorporated as a hand mix of bases consisting of 79 mol % of the intended base, and 7 mol % of each of the other three bases. "/5Phos/" denotes a 5′ phosphate group added during synthesis.

Supplementary References

1. Anders, C., Niewoehner, O., Duerst, A. & Jinek, M. Structural basis of PAM-dependent target DNA recognition by the Cas9 endonuclease. *Nature* **513**, 569-73 (2014).
2. Zuris, J.A. et al. Cationic lipid-mediated delivery of proteins enables efficient protein-based genome editing in vitro and in vivo. *Nat Biotechnol* **33**, 73-80 (2015).
